# The efflux pumps Rv1877 and Rv0191 play differential roles in the protection of *Mycobacterium tuberculosis* against chemical stress

**DOI:** 10.3389/fmicb.2024.1359188

**Published:** 2024-03-04

**Authors:** Carine Sao Emani, Norbert Reiling

**Affiliations:** ^1^Microbial Interface Biology, Research Center Borstel, Leibniz Lung Center, Borstel, Germany; ^2^German Center for Infection Research (DZIF), Partner Site Hamburg-Lübeck-Borstel-Riems, Borstel, Germany

**Keywords:** *Mycobacterium tuberculosis*, Rv1877, Rv1878, Rv0191, spermine stress, MFS-type pump, iron homeostasis, cell wall stress

## Abstract

**Background:**

It was previously shown that GlnA3_sc_ enabled *Streptomyces coelicolor* to survive in excess polyamines. However, subsequent studies revealed that Rv1878, the corresponding *Mycobacterium tuberculosis* (M.tb) ortholog, was not essential for the detoxification of spermine (Spm), in M.tb. On the other hand, the multi-drug efflux pump Rv1877 was previously shown to enable export of a wide range of compounds, while Rv0191 was shown to be more specific to chloramphenicol.

**Rationale:**

Therefore, we first wanted to determine if detoxification of Spm by efflux can be achieved by any efflux pump, or if that was dependent upon the function of the pump. Next, since Rv1878 was found not to be essential for the detoxification of Spm, we sought to follow-up on the investigation of the physiological role of Rv1878 along with Rv1877 and Rv0191.

**Approach:**

To evaluate the specificity of efflux pumps in the mycobacterial tolerance to Spm, we generated unmarked ∆*rv1877* and ∆*rv0191* M.tb mutants and evaluated their susceptibility to Spm. To follow up on the investigation of any other physiological roles they may have, we characterized them along with the ∆*rv1878* M.tb mutant.

**Results:**

The ∆*rv1877* mutant was sensitive to Spm stress, while the ∆*rv0191* mutant was not. On the other hand, the ∆*rv1878* mutant grew better than the wild-type during iron starvation yet was sensitive to cell wall stress. The proteins Rv1877 and Rv1878 seemed to play physiological roles during hypoxia and acidic stress. Lastly, the ∆*rv0191* mutant was the only mutant that was sensitive to oxidative stress.

**Conclusion:**

The multidrug MFS-type efflux pump Rv1877 is required for Spm detoxification, as opposed to Rv0191 which seems to play a more specific role. Moreover, Rv1878 seems to play a role in the regulation of iron homeostasis and the reconstitution of the cell wall of M.tb. On the other hand, the sensitivity of the ∆*rv0191* mutant to oxidative stress, suggests that Rv0191 may be responsible for the transport of low molecular weight thiols.

## Introduction

Macrophages and monocytes belong to the first line of immune defense. During infection by microorganisms, they can undergo physiological changes in order to better eradicate the invading pathogen. One of these changes is known as polarization. Macrophages are able to adapt either an M1 or M2 polarization state (Anderson and Mosser, 2002; Mosser, 2003; Mosser and Edwards, 2008; Rodríguez-Prados et al., 2010; Galván-Peña and O’Neill, 2014; Jha et al., 2015; Shi et al., 2019; Seto et al., 2022). *Mycobacterium tuberculosis* (M.tb) the causative agent of tuberculosis (TB) is able to induce the polarization state of macrophages. In an *in vitro* granuloma model, macrophages were able to switch from the M1 to the M2 physiological state upon M.tb infection (Le et al., 2020). Furthermore, M2 macrophages were found to be predominant in granulomas of TB patients, while both M1 and M2 macrophages were found in non-granulomatous lung tissues (Huang et al., 2015). Therefore, the physiological state of macrophages plays a critical role in the pathophysiology of TB (Huang et al., 2019). Polyamines (PAs), such as spermine (Spm) are also able to alter the polarization state of macrophages (Latour et al., 2020). They can be obtained either directly from food and/or synthesized in humans and some bacteria. *De novo* synthesis of PAs occurs in humans when they absorb arginine from food. The absorbed arginine is converted by arginase to ornithine which is decarboxylated (catalyzed by ornithine decarboxylase, ODC) to the diamine putrescine (Ptc), which is in turn converted by spermidine synthetase [in the presence of decarboxylated-S-adenosyl-L-methionine (Dc-SAM)] to spermidine (Spd). Then, Spm is generated through the carbonylation of Spd by Spd-synthetase in the presence of Dc-SAM (Weaver and Herbst, 1958a,b; Stewart et al., 2018; Sagar et al., 2021). The produced Spm can also undergo an oxidative decarboxylation catalyzed by Spm-oxidase (SPO) to produce again Spd (in addition to hydrogen peroxide and aminopropyl; Weaver and Herbst, 1958a,b; Stewart et al., 2018; Sagar et al., 2021). It is not known how much Spm is produced by infected and uninfected macrophages. However, it was shown that treatment of murine embryonic fibroblasts (MEFs) with 2-difluoromethylornithine, an inhibitor of ODC and diethylnorspermine, an inductor of SPO, led to a decrease in the production of Spm (Puleston et al., 2019). Since Spm has been previously demonstrated to be active against M.tb (Hirsch and Dubos, 1952; Sao Emani and Reiling, 2023), (in addition to its ability to alter the polarization state of macrophages during infection; Latour et al., 2020), it is possible that, upon phagocytosis, M.tb produces enzymes that are able to detoxify Spm, in order to better facilitate its survival in the host cell. Previous studies showed that *Streptomyces coelicolor* required GlnA3*_sc_* (SCO6962) for the detoxification of PAs (Krysenko et al., 2017). Furthermore, it was shown that GlnA3*_sc_* was able to catalyze the glutamylation of putrescine as a possible mechanism of polyamines detoxification (Krysenko et al., 2017). However, we found that the corresponding M.tb ortholog GlnA3*_Mt_* (Rv1878) was not essential for Spm detoxification (Krysenko et al., 2023). Moreover, GlnA3_sc_ shares only 40.09% similarities with GlnA3_Mt_ (by NCBI proteins-proteins amino acids alignment). In addition, the gene encoding the multi-drug efflux pump Rv3065 (de Rossi et al., 1998, 2002) was significantly upregulated during Spm stress in our previous study (Krysenko et al., 2023), while the gene encoding another multi-drug efflux pump Rv1877 (de Rossi et al., 2002; Adhikary et al., 2022) was marginally upregulated (Krysenko et al., 2023). However, since we aimed to follow-up on the investigation of the physiological role of Rv1878 while assessing if Spm is detoxified by multi-drug efflux pumps, we chose to investigate the role of Rv1877 in the context of Spm detoxification and in the context of any overlapping physiological role it may have with Rv1878 since they are encoded by neighborhood genes that are co-transcribed (Harth et al., 2005). In order to determine if tolerance of Spm by M.tb is supported by other efflux pumps, we also investigated the role of Rv0191 which encodes a more specific MFS-type efflux pump (de Rossi et al., 2002; Li et al., 2019).

Our results demonstrate that Rv1877 is able to detoxify Spm while Rv1878 and Rv1877 are involved in the regulation of iron homeostasis, the reconstitution of the cell wall, survival of M.tb in hypoxia and acidic stress. On the other hand, we found that Rv0191 contributes to the survival of M.tb during oxidative stress (OS).

## Methods

### Generation of ∆*rv1877* and ∆*rv0191 Mycobacterium tuberculosis* mutants

The generation of the ∆*rv1878* M.tb mutant has been described (Krysenko et al., 2023; 10.6084/m9.figshare.24920109), (10.6084/m9.figshare.24920151), (10.6084/m9.figshare.24920172), (10.6084/m9.figshare.24920217), and (10.6084/m9.figshare.24920277). The other mutants (∆*rv1877*, ∆*rv0191*) and respective complements were generated similarly. Generation of the mutants were according to previously published methods (Parish et al., 1999; Parish and Stoker, 2000; Muttucumaru and Parish, 2004; Goude et al., 2015; Sao Emani et al., 2018a), with minor modifications. The process was as follows: using primers 1877-USF and 1877-UFR (sequence and details found in Supplementary Table S1), a 2,416 bp fragment was amplified upstream (US) of *rv1877*. While using 1877-DSF and 1877-DSR, a 2,459 bp fragment was amplified downstream (DS) of *rv1877*, using the high fidelity Pfu GC rich target polymerase (Agilent). Similarly, the US (2,452 bp) and DS (2,470 bp) fragments of *rv0191*, were amplified using 0191-USF/0191USR, 0191DSF/0191DSR, respectively. Primers were designed to leave 50–150 bp nucleotides US and DS of each deleted gene (in the genome) to avoid polar effects on surrounding genes, and/or avoid cropping any overlapping genes. The resulting fragments were each cloned into the pJET sub-cloning vector (using the CloneJET PCR Cloning Kit, Thermo Fisher). Plasmids of potential positive colonies were extracted, screened by restriction enzyme digestion (RED), and sequenced to check and confirm integrity (Supplementary Tables S1, S2; 10.6084/m9.figshare.24920097, 10.6084/m9.figshare.24920124). Since each primer was designed with specific restriction sites not found within the inserts, yet found in the multiple cloning site (MCS) of the subsequent vector (p2NIL; Parish and Stoker, 2000), the cloned US and DS were excised using *BsrgI* and *SpeI* for *rv1877*-US, *SpeI* and *HindIII* for *rv1877*-DS, to clone both fragments using 2-way ligation into a previously digested (by *BsrgI* and *HindIII*) p2NIL vector. Similarly, *KpnI* and *SpeI* were used to excise *rv0191*-US from pJET-0191US, *SpeI* and *HindIII* to excise *rv0191*-DS from pJET-0191DS, in order to clone both fragments using 2-way ligation into a previously digested p2NIL (by *KpnI* and *HindIII*). Plasmids of potential positive colonies were extracted, screened by RED and sequenced to check and confirm integrity (Supplementary Tables S1, S2; 10.6084/m9.figshare.24920160, 10.6084/m9.figshare.24920187). On the other hand, the vector pGOAL17 (Parish and Stoker, 2000) was digested with *PacI*, and the fragment containing the *lacZ* (enables bacteria to become blue on X-galactosidase), and *sacB* genes (enables the bacteria to be sucrose sensitive) was cloned into the *PacI* restriction site of the constructs generated from p2NIL (p2NIL-1877, p2NIL-0191) to yield the final deletion constructs (p2NIL-1877US/DS-G17, p2NIL-0191US/DS-G17). Plasmids of potential positive colonies were extracted, screened again by RED, and sequenced to check and confirm integrity (Supplementary Tables S1, S2; 10.6084/m9.figshare.24920211, 10.6084/m9.figshare.24920223). The final deletion constructs p2NIL-1877US/DS-G17, p2NIL-0191US/DS-G17 were used to delete a 2004 bp fragment of *rv1877*, and a 1,098 bp fragment of *rv0191* (respectively), using a two-step homologous recombination-based method as previously described (Muttucumaru and Parish, 2004; Sao Emani et al., 2018a). The resulting M.tb mutants were screened by PCR, and validated by southern blotting as previously described (Sao Emani et al., 2018c) with the only difference in the last step, where a colorimetry based method was used for the detection of the bands using the DIG High Prime DNA labelling and detection kit I (Roche), instead of the chemiluminescence method using DIG High Prime DNA labelling and detection II (Roche).

The complemented strains were generated by amplifying the *rv0191* or *rv1877* using primers 0191-F/0191-R or 1877-F/1877-R, respectively, (Supplementary Table S1) with added *HindIII*/*HpaI* restriction sites on the forward and reverse primers, respectively, and an optimized ribosomal binding sequence (Highlighted in red in Supplementary Table S1) on each forward primer (1877-F and 0191-F) using the pfu high fidelity GC rich target polymerase. Then the resulting fragments were sub-cloned into pJET. Plasmids of potential positive colonies were extracted, screened by RED, and sequenced to check and confirm integrity (Supplementary Tables S1, S2; 10.6084/m9.figshare.24920133, 10.6084/m9.figshare.24920157). Then each fragment was excised from pJET (using *HindIII* and *HpaI*,) and cloned downstream of the *hsp*60 promoter of pMVhsp (Andreu et al., 2010) kan^r^ mycobacterial integrating vector (pre-digested with *HindIII* and *HpaI*). Then, plasmids of potential positive colonies were extracted, screened again by RED, and sequenced to check and confirm integrity (Supplementary Tables S1, S2; 10.6084/m9.figshare.24920259, 10.6084/m9.figshare.24920280). Plasmids extracted from the positive colonies were used to transform the corresponding M.tb mutants in order to generate the complemented strains which were screened by amplifying a 151 bp fragment of the kanamycin cassette of pMVhsp60 (Supplementary Table S1) and by confirming their resistance to kanamycin. The phage and endonuclease resistant DH10β *E.coli* strain (able to carry large plasmids; New England Biolabs) was used for all cloning experiments described in this study.

### General characterization of the various strains

#### Growth curves generation

Growth curves were obtained by sub-culturing a starter cultures (initiated from frozen stocks) in 7H9-ADS-Tyl [supplemented with 1X albumin-dextrose-sodium chloride (ADS) and 0.06% tyloxapol as previously described (Sao Emani et al., 2018d, 2022)]. The preparation of the 10X ADS supplement consisted to add 25 g Bovine Albumin Fraction V (Sigma Aldrich), 10 g Dextrose, (Sigma Aldrich) and 4.25 g Sodium Chloride (Sigma Aldrich) to a final volume of 490 mL distilled water. After mixing the suspension to homogeneity, the supplement was sterilized by filtration and stored at 4°C for all experiments that required the ADS-supplement. The growth curves were also performed in Sauton’s media-Tyl (HiMedia Laboratories Pvt. Ltd), prepared by dissolving 3.19 g of the dehydrated media in 980 mL distilled water +20 mL glycerol. A volume of 3 mL of tyloxapol (Tyl) from a 20% filter-sterilized stock, was added to the Sauton’s media after it was sterilized by autoclaving. Before, mycobacteria were sub-cultured for growth curves evaluation in Sauton’s-Tyl media, residual 7H9 media was washed off. Mycobacteria were either treated with Spm (80 μM for Sauton’s and 3 mM for 7H9) or with the respective DMSO controls and the OD_600_ was measured every second day. Mycobacteria were incubated without agitation as previously described (Sao Emani et al., 2018a). A maximum volume of culture of 15 mL was used in T25 tissue culture flasks, while a maximum volume of 35 mL was used in T75 tissue culture flasks.

#### Culture conditions-dependent susceptibility tests

Susceptibility tests were performed as follows. Logarithmic phase cultures in Sauton’s media were adjusted to an OD_600_ of 0.2 (cultured without agitation in vented tissue culture flasks). This was washed several times in sterile 1X PBS (phosphate buffer saline, prepared from 10X Gibco™ DPBS, without calcium and magnesium; +0.6% tyloxapol). Then a 100-fold dilution was performed, either in PBS, or in 0.5% SDS, or in acidified 7H9, or Sauton’s (pH ~5) or in the iron-deprived (IS) media prepared as previously described (Kurthkoti et al., 2017) with slight modifications as follows. A volume of 425 mL of distilled water was added to a mixture of 2.5 g Asparagine +2.5 g potassium phosphate dehydrate +20 mL glycerol. Then the pH was adjusted with sodium hydroxide to 6.8. Next, 20 g of Chelex^®^ 100 (Sigma Adrich, chelates metal) was added to the mixture which was incubated overnight at 4°C. The following day, the media was filtered and 5 μL of sterile (previously prepared and stored at −20°C) 50 mg/mL ZnCl2, 50 mg/mL MnSO4 and 50 mg/mL MgSO4 each, were added to the media. The final IS media was supplemented by adding 50 mL of sterile 10X ADS and 1.3 mL of 20% filtered sterilized tyloxapol. The mycobacteria resuspended to desired bacterial density in various media, were aliquoted (2 mL) in 12-well plates, and incubated at 37°C. Aliquots were collected at various time points and plated accordingly for CFUs determination. Percentage survivals were derived relative to the CFUs obtained from day-1 of the experiment. Similarly, for anaerobic experiments, cultures resuspended in Sauton’s media-Tyl and aliquoted in 96-well plates were incubated in hypoxic conditions (without agitation) using the Anaerogen Gas Pack System (Thermo Fisher) as previously described (Tan et al., 2010). Shortly, after the mycobacterial plates were sealed with micropore tapes, they were placed in the Thermo Scientific™ Oxoid Anaerobic 2.5 L Rectangular Jar, then the pouch from the Thermo Scientific™ Oxoid AnaeroGen 2.5 L anaerobic gas generating sachet was quickly placed in the reserved chamber of the jar/container, which was rapidly sealed and incubated at 37°C. In addition, the Thermo Scientific™ Oxoid™ Resazurin Anaerobic Indicator was removed from its sachet and quickly stuck to the internal wall of the container (before it was sealed) to visually monitor depletion of oxygen by its color change from pink to white. Moreover, before the actual experiment, an oxygen measuring device (portable oxygen meter, Brand: Rrunzfon, Tyle: Industrial), was used to measure the level of oxygen in the system over time to ensure that the system was depleting oxygen to hypoxia levels. The CFUs after 48 h of incubation in hypoxia were normalized to the initial CFUs (day-1) to obtain the percentage survival.

#### Stressors-dependent susceptibility tests

Cultures diluted in Sauton’s media-Tyl, were also exposed for 3 h to various stress conditions, such as Spm stress (2 mM) and oxidative stress (OS), [generated by cumene hydroperoxide (CuOOH; Weiss and Estabrook, 1986a), 2 mM] and nitrosative stress generated by tert-butyl nitrite (TBN, 10 mM; Liu, 2011). For each condition, a DMSO/untreated control was included. This was used to derive the percentage survival under each condition, by dividing the CFUs count of the treated samples to the corresponding untreated/DMSO controls. To further study the susceptibility of the mutants to an extended exposure to Spm stress during nutrient starvation (NS), cultures were washed several times in PBS, resuspended to an OD_600_, of 0.2 and diluted 100X for an exposure to NS for up to 7 days, with and without Spm (using lower concentrations, 100-500 μM). Initial experiments were performed at least twice only in PBS, to examine/evaluate the survival of each strain in PBS for an extended period. Then the experiment was repeated at least twice again in PBS treated with Spm. Percentage survivals were determined relative to the CFUs obtained from day-1.

#### Quantification of ferric and ferrous iron

Logarithmic phase Sauton’s cultures were used to quantify the levels of ferric and ferrous iron in the wild-type and the ∆*rv1878* mutant. As a control, a plain Sauton’s media (contains iron), and a plain IS media (did not contain iron) were used. A volume of 100 μL of the mycobacteria were aliquoted in a transparent 96-well plate and quantification was achieved using the iron assay kit (Sigma-Aldrich Co. LLC) following the manufacturer’s instructions. Briefly, two conditions were measured per sample. A well to measure the total level of iron, and the other well to measure the level of ferrous iron. While a blank/buffer was added to the total iron well, an equal volume of a reducer (provided by the kit) was added to the ferrous iron well. This was incubated at room temperature for ≥30 min. Then a range of concentrations of the iron standard (provided by the kit) was prepared and added in the same plate. Finally, the detection probe (also provided by the kit) was added to each well, including the wells of the standard. It was then mixed properly while avoiding air bubbles (that could interfere with the read). And the plate was incubated for another hour. Using a plate reader, the absorbance at 593 nm was measured. After data collection, the value of the blank value was first subtracted from all samples, then a standard curve was plotted on Excel. The equation of the standard curve was used to derive the concentration of each sample. Then to obtain the concentration of ferric iron, the calculated value from the ferrous iron well was subtracted from the value of the total iron well. Finally, the values obtained from the mutant were normalized to the wild-type’s to obtain the fold difference.

#### Samples preparation for RNA sequencing and RT-PCR

This protocol is a slight modification to our previous description (Krysenko et al., 2023). A volume of 10 mL of logarithmic phase cultures was centrifuged and the pellet obtained was resuspended in the buffer provided in the RNA Pro Blue kit (MP Bio). The resuspended cells were homogenized by the Fast Prep Homogenizer (time: 30 s, speed: 6 m/s, 5 min intermittent on ice, frequency: 4 times). After centrifugation to pellet cellular debris, the cell lysate was filtered twice using PTFE syringe filters (13 mm diameter, 0.2 μM pores size) and taken out of the BSL3 laboratory for further purifications. The first purification was performed using the Direct-zol RNA Miniprep Plus (R2070, 100 μg binding capacity) including an in-column DNA digestion step, according to the manufacturer’s instructions. The purified samples were quantified using a spectrophotometer, and diluted if the concentration exceeded 200 ng/μl. Then they were further digested using the Turbo DNA-free kit (Thermo Fisher) according to manufacturer’s instructions, however in two consecutive rounds, to ensure complete DNA digestion. The digested samples were further purified and concentrated using the RNA clean and concentrator kit-25 (R1017, 50 μg binding capacity) according to manufacturer’s instruction, and another in-column DNA digestion step was included. The resulting samples were checked for integrity and purity by running an agarose gel (to check for the sharpness of the 16S and 23S ribosomal RNA bands) and/or by spectrophotometry (looking at the A260/280 and A260/230 ratios). In order to perform the reverse-transcriptase quantitative PCR, (RT-PCR), 300–500 ng of RNA of each sample was converted to cDNA using the Maxima First Strand cDNA KIT (Thermo Fisher). During method optimization, controls containing all reagents except the reverse transcriptase were included (non-reverse transcriptase control), and were run along with the converted samples, to ensure that genomic DNA was completely removed or negligible. The reverse transcribed samples were run in a 10 μL reaction on a LightCycler 480 using the LightCycler 480 master mix. Quantification was made by the integrated software of the LightCycler according to a probe-based assay (labelled at the 5′-end with FAM and at the 3′-end with a quencher), using M.tb genomic DNA to generate a standard curve (10–1,000 pg./μl). We optimized the probes and primers designed by TIB MOLBIOL Syntheselabor GmbH (Supplementary Table S2) for each assay.

For the determination of the expression levels of specific genes during hypoxia, logarithmic phase cultures were aliquoted in 6-well plates and placed in either the hypoxic system (Anaerogen Gas Pack System), or in an agitated and aerated container. After incubation at 37°C for 24 h, RNA extraction and subsequent RT-PCR analyses, were performed as described above. The result presented in this study (fold change of each gene), was obtained by normalizing the expression of each gene in the hypoxic condition to the corresponding expression in the agitated aerated condition.

For the determination of the expression levels during oxidative stress or nitrosative stress, mycobacteria were treated with a range of concentration of CuOOH (0.5–5 mM) for 3 h or with 20 mM TBN for 1 h. RNA was extracted from both the treated samples and the untreated controls. Extraction of RNA, conversion to cDNA and quantification of expression levels were performed as described above. The fold change was derived relative to the untreated control.

To confirm absence of polar effect, late logarithmic-stationary phase cultures of each strain were processed as described above to investigate the expression level of specific genes in each strain.

To evaluate the expression profile of each mutant relative to the wild-type’s, logarithmic phase cultures (Sauton’s media) were used to extract RNA as described above. Pure RNA samples from 4 independent experiments were sent for sequencing by Eurofins Genomics GmbH who performed further quality checks, RNA sequencing and data analyses as they have previously described (Dobin et al., 2013; Chen et al., 2018; Raw data found here: 10.6084/m9.figshare.24920028). The depth of sequencing was 30 to 60 million reads (reads length ˃30 bp). This allowed for detection of low abundant transcripts. However, since we left 50–150 bp nucleotides upstream and downstream of each gene deleted, the residual transcripts of fragments of *rv1877*, *rv1878* and *rv0191* that were left in the genome during the generation of the mutants, were also detected in the corresponding mutant due to the very high depth of sequencing. Therefore, the analyzed data obtained from Eurofins, which was the entire expression profile of each strain (relative to the wild-type, along with their calculated *p*-values) was further refined by removing the expression data of the residual fragment of *rv1877* in the ∆*rv1877* mutant, the residual fragment of *rv1878* in the ∆*rv1878* mutant and the residual fragment of *rv0191* in the ∆*rv0191* mutant since these genes are deleted in the respective strains. Two set of data were obtained: all data including the non-statistically significant data and the statistically significant data. We focused our analyses on the statistically significant data.

### Statistical analysis

For the RNA sequencing data analysis, statistical analyses were performed by Eurofins as follows. First, the abundance counts of each gene were used to perform differential gene expression (DGE). DGE was performed using R/Bioconductor package edgeR (Robinson et al., 2010), the calcNormFactors function was normalized for RNA composition by finding a set of scaling factors for the library sizes that minimized the log-fold changes between the samples for most genes. Statistical tests were performed for each gene to compare the distributions between conditions (treatment vs. control) generating p-values for each gene. The final p-values were corrected by determining false discovery rates (FDR) using the Benjamin–Hochberg method.

For other data presented in this study, two types of analyses were performed. For experiments where only one time point was investigated each mutant was compared to the wild-type by computing an unpaired *t*-test (assuming both strains have the same standard deviation), using a parametric test, with a two-tailed comparison.

For experiments where two time points or more were investigated, a multiple *t*-test was used to also compare each strain to the wild-type. The false discovery rate (FDR) approach was the Two-stage step-up (Benjamini, Krieger, and Yekutieli). For all comparisons, FDR or alpha was set to 0.05, ^*^p < 0.05, ^**^p < 0.01, ^***^p < 0.001, ^****^p < 0.0001.

## Results

### Transcriptomics reveal a signature for *Mycobacterium tuberculosis* mutants

**Figure 1:**
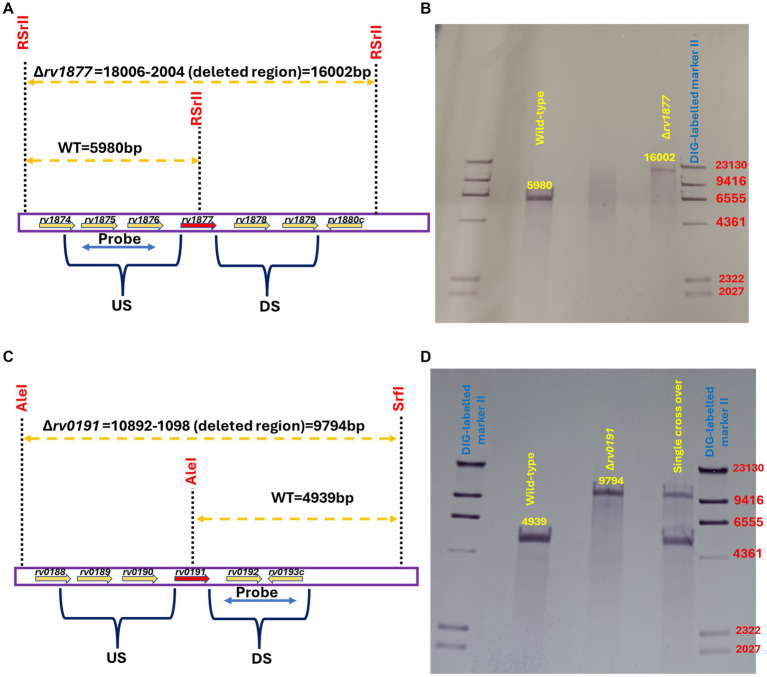
Genotyping of mutants. **(A)** Southern blot design for the identification of the *∆rv1877* mutant. The restriction enzyme RSrII was used to digest M.tb genomic DNA, and the PCR fragment flanking the upstream region (US) was used as the probe. **(B)** Genotyping reveals a 5,980 bp band for the wild-type (WT) and a 16,002 bp band for the mutant. **(C)** Southern blot design for the identification of the *∆rv0191* mutant. The restriction enzymes AleI and SrfI were used to digest M.tb genomic DNA, and the PCR fragment flanking the downstream region (DS) was used as the probe. **(D)** Genotyping reveals a 4,939 bp band for the wild-type (WT) and a 9,794 bp band for the mutant.

In our previous study, we showed that the M.tb gene encoding Rv1877, [a PMF (proton motive force)-dependent multi-drug transporter (Adhikary et al., 2022)], and other multi-drug transporters were upregulated during Spm stress (Krysenko et al., 2023). Therefore, we wanted to determine if tolerance of Spm by M.tb was supported by general efflux activities or by specific efflux pumps. To achieve that, we generated an in-frame unmarked deletion of *rv1877* (Figures 1A,B; Supplementary Figures S1A,B) and in-frame unmarked deletion of *rv0191* in M.tb (Figures 1C,D; Supplementary Figures S1A,D) since Rv0191 is an efflux pump that seems to be able to extrude only chloramphenicol (Li et al., 2019). Moreover, since Rv1878 was found not to be essential for Spm detoxification (Krysenko et al., 2023), we sought to investigate its physiological role. Therefore, we aimed to characterize our previously generated ∆*rv1878* mutant in other physiological conditions along with the ∆*rv1877* and the ∆*rv0191* mutants. To facilitate interpretation of the phenotypes of these mutants, we studied their transcriptomic profile by RNA sequencing. Many genes were differentially regulated in the mutants relatively to the wild-type (Supplementary Tables S3–S5). When we narrowed our analyses to genes that displayed a ≥ 5-fold upregulation (Table 1), with few exceptions of lower fold changes in cases where there was an overlap in the regulations (Supplementary Tables S3–S5), we found ~4 genes that were upregulated in all strains (Supplementary Tables S3–S5), suggesting a transcriptomic signature for mutants. They were *rv0067c*, *rv0096*, *rv2780*, *rv3503*, *rv2628*, *rv3746*, *rv3574*, *rv0280*-*rv0291*, *rv1405*, *rv2057*, *rv1057*, *rv0106* and *rv1854c* (Table 1; Supplementary Tables S3–S5). The gene *rv0067c* encodes a TetR family transcriptional factor (OxiR) that was shown to regulate the resistance of M.tb to isoniazid (INH; Yang et al., 2019). Moreover, it was shown to negatively regulate the oxidoreductase Rv0068 in order to alter the sensitivity of M.tb to INH (Yang et al., 2019). The gene *rv0096* encodes a putative member of the PPE (proline-glutamate and proline-proline-glutamate) family and is part of the operon *rv0096*–*rv0101* that was shown to produce a virulence-related lipopeptide (Hotter et al., 2005; Wang et al., 2007; Liu et al., 2013; Harris et al., 2017). In addition, it was shown that a loss of function mutation of *rv0096* in multi-drug resistant M.tb strains conferred resistance to D-cycloserine (Desjardins et al., 2016). The gene *rv2780* encodes l-alanine dehydrogenase that catalyzes the oxidative deamination of l-alanine to pyruvate that is channeled towards the production of peptidoglycan (Giffin et al., 2012). The protein Rv2780 was also shown to catalyze the reductive amination of glyoxylate to glycine and to be upregulated during hypoxia, nutrient starvation and in media containing alanine as sole nitrogen source. It seems to play many physiological roles mainly during C/N metabolism (Betts et al., 2002; Starck et al., 2004; Giffin et al., 2012). The gene *rv3503c* encodes a ferredoxin FdxD which is an iron–sulfur cluster protein that supports the function of cytochrome p450 enzymes (Ortega Ugalde et al., 2018). The gene *rv2628* encodes an immunogenic protein (Goletti et al., 2010; Bhatt et al., 2022) belonging to the group of DosR-dependent proteins, which are activated during dormancy to maintain M.tb in a dormant state (Voskuil et al., 2003). The gene *rv3746c* is part of the PE_PPE immunomodulatory genes suggested to play crucial roles in the host-pathogen interaction (Choi and Shin, 2015). The gene *rv3574* (kstR) has been shown to control the expression of genes involved in lipid metabolism (Kendall et al., 2007). The Esx-3 gene *cluster rv0280*-*rv0291* belonging to type VII secretion systems were found to be up regulation in a ∆*zur* mutant (where Zur/Rv2359 is a zinc uptake regulator; Maciąg et al., 2007). Moreover, *rv0282* is involved in iron metabolism and is able to secrete iron-dependent effectors that modulate the virulence of M.tb (Tufariello et al., 2016). In addition, this operon has been implicated in copper homeostasis as well (Festa et al., 2011; Limón et al., 2023). The gene *rv1405c* codes for a virulence associated methyltransferase that plays a role during the adaptation of M.tb to acidic stress (Healy et al., 2016). The gene *rv2057c* encodes a ribosomal protein which is mainly involved in protein synthesis but have been suggested to play other physiological roles (Fan et al., 2014). The gene *rv1057* codes for a protein that belongs to a structurally distinct group of proteins known as β-propellers (Haydel and Clark-Curtiss, 2006) and plays a role in the secretion of the virulence factor ESAT-6 (Fu et al., 2018). The gene *rv0106* encodes a hypothetical protein that was found to be upregulated in an M.tb ∆*zur* mutant (Maciąg et al., 2007). The gene *rv1854c* encodes NADH dehydrogenase (Ndh) which is an enzyme of the electron transport chain and has been suggested to be the main NADH dehydrogenase of M.tb, since an ∆*ndh* mutant has a growth defect *in vitro* and *in vivo* and is sensitive to alterations in the cell redox state and to oxidative stress (Vilchèze et al., 2018).

**Table 1:** Genes that are significantly upregulated in the mutants relative to the wild-type.

∆*rv*1877 relative to the wild-type			
*p* value	Gene	Gene product	FC ≥ 5
2.89E-33	*rv1878*	Probable glutamine synthetase GlnA3 (glutamine synthase; GS-I)	21.13
0.0006	*rv1057*	Conserved hypothetical protein	8.41
0.0012	*rv0096*	PPE family protein PPE1	8.29
0.0165	*rv1405c*	Putative methyltransferase	8.08
0.0237	*rv2057c*	50S ribosomal protein L33 RpmG1	6.92
0.0114	*rv2780*	Secreted L-alanine dehydrogenase Ald (40 kDa antigen; TB43)	6.78
0.0432	*rv2058c*	50S ribosomal protein L28 RpmB2	6.20
0.0014	*rv3503c*	Probable ferredoxin FdxD	6.16
0.0068	*rv0282*	ESX conserved component EccA3. ESX-3 type VII secretion system protein.	5.89
0.0245	*rv2628*	Hypothetical protein	5.74
0.0186	*rv1738*	Conserved protein	5.67
0.0357	*rv3746c*	Probable PE family protein PE34 (PE family-related protein)	5.62
0.0048	*rv3574*	Transcriptional regulatory protein KstR (probably TetR-family)	5.54
0.0082	*rv3065*	Multi-drug transport integral membrane protein Mmr	5.28
0.0238	*rv0106*	Conserved hypothetical protein	5.02
1E-02	*rv0285*	PE family protein PE5	4.9
1E-03	*rv0067c*	Possible transcriptional regulatory protein (possibly TetR-family)	4.9
7E-03	*rv1854c*	Probable NADH dehydrogenase Ndh	4.7
*∆rv1878 relative to the wild-type*			
0.01787	*rv1405c*	Putative methyltransferase	7.85
0.0010	*rv1057*	Conserved hypothetical protein	7.51
0.0006	*rv3503c*	Probable ferredoxin FdxD	7.14
0.0250	*rv3746c*	Probable PE family protein PE34 (PE family-related protein)	6.37
0.0048	*rv0096*	PPE family protein PPE1	6.15
0.0168	*rv2780*	Secreted L-alanine dehydrogenase Ald (40 kDa antigen; TB43)	6.03
0.0222	*rv2628*	Hypothetical protein	5.93
0.0161	*rv1738*	Conserved protein	5.92
0.0366	*rv2057c*	50S ribosomal protein L33 RpmG1	5.86
0.0054	*rv3574*	Transcriptional regulatory protein KstR (probably TetR-family)	5.40
0.0100	*rv0282*	ESX conserved component EccA3. ESX-3 type VII secretion system protein.	5.37
0.0098	*rv3065*	Multi-drug transport integral membrane protein Mmr	5.08
1.1E-03	*rv0067c*	Possible transcriptional regulatory protein (possibly TetR-family)	4.9
1.3E-02	*rv0285*	PE family protein PE5	4.7
*∆rv0191 relative to the wild-type*			
0.0283	*rv2058c*	50S ribosomal protein L28 RpmB2	7.35
0.0281	*rv2057c*	50S ribosomal protein L33 RpmG1	6.49
0.0461	*rv2056c*	30S ribosomal protein S14 RpsN2	5.59
0.0291	*rv0280*	PPE family protein PPE3	5.54
0.0058	*rv1057*	Conserved hypothetical protein	5.25
0.0559	*rv1405c*	Putative methyltransferase	5.07
4.2E-02	rv2628	Hypothetical protein	4.8

*FC, fold change.

On the other hand, we also observed that many genes were found to be downregulated in all three mutants when we set our threshold to −5-fold (Supplementary Tables S3–S5), therefore due to space constrains we will not discuss all of them but narrow our analyses to genes that fell within a threshold of ≤ −10-fold. In this case, ~10 genes were downregulated in all three mutants (Table 2; Supplementary Tables S3–S5), namely the gene cluster *rv0847*-*rv0848*-*rv0849*-*rv0850*, the genes *rv1519*, *rv0448c*, *rv0186A*, *rv3054c*, *rv0186A* and *rv0150c*. The gene *rv0847* codes for the putative lipoprotein lpqS, and it is co-transcribed with *rv0848*, *rv0849* and *rv0850* (Sakthi and Narayanan, 2013). The ∆*rv0847* mutant has been generated and characterized. It has a growth defect in Sauton’s media, in THP1 macrophages and is sensitive to SDS and copper (Sakthi and Narayanan, 2013). Similarly, the ∆*rv0848* (∆*cysK*_2_) mutant was shown to have an altered cell wall lipid profile, to be sensitive to oxidative stress, vancomycin, rifampicin and have a growth defect in Raw264.7 macrophages (Sao Emani et al., 2022). The gene *rv0849* codes for an MFS-type efflux pump. The ∆*rv0849* mutant displays a slight sensitivity to pyrrole, pyrazolone and rifampicin (Balganesh et al., 2012). Very little is known about the last gene in the cluster *rv0850*, besides its putative transposase role. This gene cluster has been termed copper toxicity response genes in another study where it was shown that induction of SigC led to their up regulation (Grosse-Siestrup et al., 2021). Since genes in this cluster are co-transcribed (Sakthi and Narayanan, 2013), and the entire operon is downregulated in the mutants, this suggests that it is a general stress sensing operon, the stress here being the loss of a gene/loss of fitness of M.tb. The gene *rv1519* encodes a hypothetical protein that was suggested to be associated with the persistence and transmission rate of an East African-Indian lineage of M.tb (Newton et al., 2006). The gene *rv0448c* encodes a hypothetical protein that was shown to be upregulated in *M. bovis* relatively to M.tb when cultured in 7H9-ADS-tween 80 (Rehren et al., 2007). The gene *rv0186A* encodes a copper-binding metallothionein MymT (Gold et al., 2008) that is upregulated upon induction of SigC (Grosse-Siestrup et al., 2021). The gene *rv3054c* is over-expressed upon induction of the VapBC4 toxin–antitoxin system. It encodes a protein of unknown function. In summary, genes that display the same dysregulation pattern in all three mutants code for proteins involved in the general detoxification of M.tb and/or in the fitness of M.tb as a pathogenic strain. This is perhaps a general loss of fitness expression signature, since it occurred in all mutants, irrespective of the function of the gene lost.

**Table 2:** Genes that are significantly downregulated in the mutants relative to the wild-type.

∆*rv*1877 relative to the wild-type			
*p*-value	Gene	Gene product	FC ≤ 10
6E-04	*rv1519*	Conserved hypothetical protein	−9.9
6E-03	*rv0850*	Putative transposase (fragment)	−11.4
2E-04	*rv3054c*	Conserved hypothetical protein	−13.8
3E-02	*rv0620*	Probable galactokinase GalK (galactose kinase)	−17.4
3E-05	*rv0848*	Possible cysteine synthase a CysK2 (O-acetylserine sulfhydrylase; O-acetylserine (thiol)-lyase; CSASE)	−17.8
6E-04	*rv0448c*	Conserved hypothetical protein	−30.1
7E-03	*rv0745*	Conserved hypothetical protein	−33.2
5E-05	*rv0186A*	Metallothionein%2C MymT	−35.6
7E-03	*rv2653c*	Possible PhiRv2 prophage protein	−36.8
2E-05	*rv0847*	Probable lipoprotein LpqS	−45.0
4E-05	*rv0150c*	Conserved hypothetical protein	−95.9
***∆**rv1878 relative to the wild-type*			
8.5E-05	*rv0849*	Probable conserved integral membrane transport protein	−10.2
5.4E-03	*rv0850*	Putative transposase (fragment)	−12.1
8.2E-05	*rv0848*	Possible cysteine synthase a CysK2 (O-acetylserine sulfhydrylase; O-acetylserine (thiol)-lyase; CSASE)	−14.2
8.0E-03	*rv2123*	PPE family protein PPE37	−15.5
3.4E-02	*rv2660c*	Hypothetical protein	−16.7
2.7E-02	*rv1119c*	Hypothetical protein	−17.1
2.3E-03	*rv0448c*	Conserved hypothetical protein	−17.9
5.2E-04	*rv0186A*	Metallothionein%2C MymT	−18.5
1.1E-02	*rv1755c*	Probable phospholipase C 4 (fragment) PlcD	−22.7
1.4E-04	*rv0847*	Probable lipoprotein LpqS	−25.5
1.3E-02	*rv1037c*	Putative ESAT-6 like protein EsxI (ESAT-6 like protein 1)	−26.0
1.5E-02	*rv1041c*	Probable is like-2 transposase	−34.4
*∆rv0191 relative to the wild-type*			
8.6E-04	*rv3054c*	Conserved hypothetical protein	−9.7
9.1E-05	*rv0848*	Possible cysteine synthase a CysK2 (O-acetylserine sulfhydrylase; O-acetylserine (thiol)-lyase; CSASE)	−13.9
9.6E-04	*rv0186A*	Metallothionein%2C MymT	−15.5
7.7E-04	*rv0847*	Probable lipoprotein LpqS	−15.7
6.7E-03	*rv1755c*	Probable phospholipase C 4 (fragment) PlcD	−22.9
4.3E-03	*rv0962c*	Possible lipoprotein LprP	−36.9

On the other hand, with the threshold of the analysis set to ±5-fold, the genes that were dysregulated only in a mutant or two were: *rv2546* (−6-fold) *and rv0962* (−40-fold) that were found to be downregulated only in the ∆*rv0191* mutant. The gene *rv2546* encodes VapC18 which is part of toxin-antitoxin modules belonging to the VapBC family. It is believed that proteins in these modules can be toxic to M.tb when over-expressed (the VapCs), and this toxicity can be neutralized by other proteins of the same modules (the VapBs; Ahidjo et al., 2011). The gene *rv0962* encoding a putative lipoprotein, was marginally upregulated (3-fold) in mouse macrophages (Schnappinger et al., 2003). On the other hand, *rv0620* (−17-fold), found to be upregulated during Spm stress in our previous studies (Krysenko et al., 2023) encoding a putative galactose kinase (GalK) and *rv0335c* (−5-fold) encoding the PE family protein PE6 which is able to suppress the innate immune defense (Sharma et al., 2021) were downregulated only in the ∆*rv1877* mutant. Moreover, though *rv0150c* was downregulated in all mutants, it displayed a significantly high downregulation in the ∆*rv1877* mutant (−100-fold as compared to 4 to 8-fold in other strains). However, there is no information about the role of this protein. The gene *rv2058c* was found to be upregulated in both the ∆*rv1877* (6-fold) and the ∆*rv0191* (7-fold) mutants (Table 1). It is part of the operon *rv2055c*-*rv2058c*, which was highly expressed in response to the Zn^2+^and Mn^2+^-binding protein calprotectin, which is an important feature of necrotic granulomas (Dow et al., 2021). The genes *rv1738* (6-fold) and *rv3065* (5-fold) were found to be upregulated in both the ∆rv*1877* and ∆*rv1878* mutants. The gene *rv1738* is one of the most upregulated genes during hypoxia and dormancy (Sherman et al., 2001; Voskuil et al., 2003). And *rv3065* codes for a multi-drug SMR-type efflux pump that confers resistance to various antibiotics when over-expressed in *M. smegmatis* (de Rossi et al., 1998; Balganesh et al., 2012; Rodrigues et al., 2013). The genes that were downregulated in both the ∆*rv1877* and ∆*rv1878* mutants are *rv3108* (−4-5-fold), *rv0327c* (−4-6-fold), *rv2653c* (−37-fold for *∆rv1877*, and − 8-fold for *∆rv1878*). and *rv0745* (−33-fold for *∆rv1877*, and − 7-fold for *∆rv1878*). The gene *rv3108* is part of a 15-kb genomic island *rv3108-rv3126c* which encodes enzymes involved in the biosynthesis of molybdenum cofactor that is able to sustain M.tb during nitrate respiration and enable persistence during hypoxia (Levillain et al., 2017). The gene *rv0327c* encodes an isoform of cytochrome P450 (135A1 Cyp135A1) that is induced during diamide stress (Mehra and Kaushal, 2009). The gene *rv2653* encodes also a hypothetical protein with unknown function, but seems to interact with proteins involved in the detoxification of M.tb according to the string database.^1^ The gene *rv0745* is located upstream a PG_PGRS gene and could therefore play similar roles (Delogu et al., 2006).

The genes *rv0661c* encoding the toxin VapC7 (−6-fold), *rv0662c* encoding the antitoxin VapB7 (−7 fold), *rv1734c* (−6-fold) encoding a hypothetical protein [which seems to be implicated in dormancy (Florczyk et al., 2003)] and *rv2270* (−5-fold) encoding lipoprotein LppN which is able to bind to macrophages to prevent entry of M.tb (Ocampo et al., 2014), were found to be downregulated only in the ∆*rv1878* mutant. Moreover, the following genes were also downregulated only in the ∆*rv1878* mutant: *rv1037c* (−26-fold), *rv1041c* (−34-fold), *rv2123* (−15-fold), *rv2660c* (−17-fold), and *rv1119c* (−17-fold). The gene *rv1037c* encodes an ESAT-6-like protein (EsxL) predicted to be an adhesin (Kumar et al., 2013) and has been suggested to play a role during active TB infection of the lungs (Bukka et al., 2011). The gene *rv1041c* encodes an unknown protein that seems to interact with transposase proteins according to the string database.^2^ The gene *rv2123* encodes PPE37 that is required for iron acquisition from heme in the Erdman strain of M.tb (Tullius et al., 2019). The gene *rv2660c* is a latency associated gene, that was found to play a role in the modulation of the immune response during infection (Yihao et al., 2015). The gene *rv1119c* is a pseudogene (Shenoy et al., 2004), and was most likely picked up during the RNA sequencing because of the high sequencing depth that was applied to cover as many genomic regions as possible. Moreover, *rv1755c* is downregulated in both the ∆*rv1878* and ∆*rv0191* mutants. It encodes an inactive phospholipase (PlcD) because this gene is truncated by the *IS6110* insertion element (le Chevalier et al., 2015). In brief, genes that are differentially regulated only in ∆*rv1878* mutant and/or the ∆*rv1877* mutant are mostly involved in the survival of M.tb during hypoxia, nutrient starvation, dormancy, during iron homeostasis dysregulation and nitrogen metabolism (Supplementary Tables S3–S5).

### Rv1877 protects *Mycobacterium tuberculosis* against spermine stress

**Figure 2:**
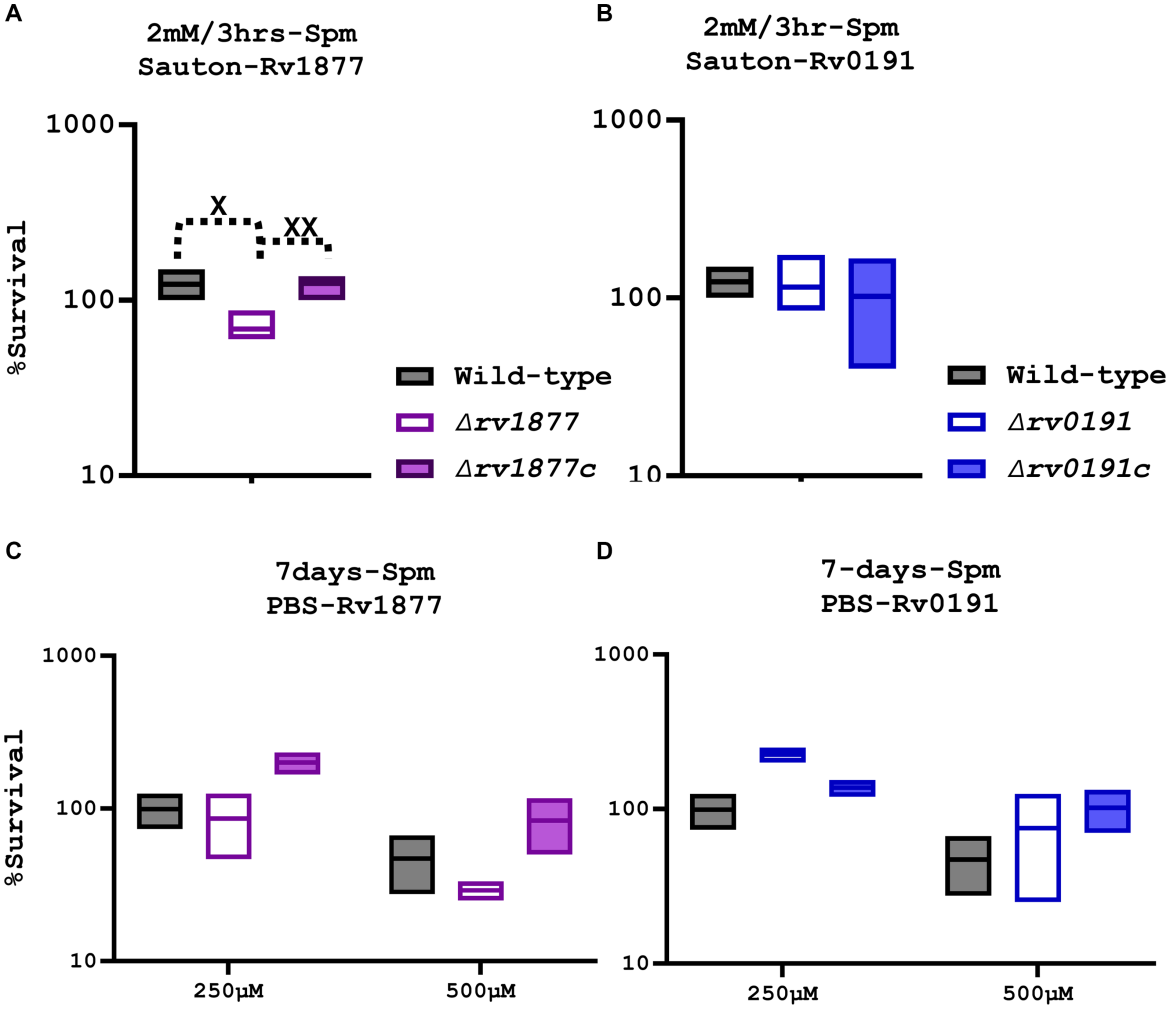
Susceptibility of the mutants to Spm stress. **(A)** The *∆rv1877* mutant was exposed for 3 h to 2 mM Spm in Sauton’s media. Survival percentage was evaluated relative to the untreated DMSO control. A *t*-test between the wild-type and the mutant was performed using Prism 10 to determine statistical significance resulting to *p* = 0.015, and between the complement and the mutant to yield a *p* value of 0.0071. **(B)** The *∆rv0191* mutant was treated similarly. There was no difference observed. **(C)** The *∆rv1877* mutant was exposed for 7 days to 250 μM and 500 μM Spm in PBS. The survival percentage was derived relative to the CFUs obtained from day-1. A marginal sensitivity relative to the wild-type and an almost (*p* = 0.06, 2-way Anova) statistically significant sensitivity relative its complement was found. **(D)** The *∆rv0191* mutant was treated similarly, and it survived better than the wild-type under this condition. Alpha was set to 0.05, ^*^p < 0.05, ^**^p < 0.01, ^***^p < 0.001, ^****^p < 0.0001 during the *t*-test.

The ability of the mutants to survive during Spm stress was initially investigated by analyzing their growth curves in both the Sauton’s-Tyl media and 7H9-ADS-Tyl media with and without Spm (added at half the MICs that were previously determined (Krysenko et al., 2023)). The OD_600_ measurement did not reveal a significant growth defect of the mutants in all tested culture conditions (Supplementary Figures S2A–D), instead the ∆*rv1878* mutant seemed to grow better in Sauton’s media treated with Spm (Supplementary Figure 2D) as previously reported (Krysenko et al., 2023). Therefore, to further investigate the sensitivity of these strains using a more accurate method, we exposed the mutants and their respective complements to a higher concentration of Spm (2 mM instead of 80 μM) for a shorter period (3 h) in Sauton’s media, and evaluated their survival by more reliable CFUs counts, which were normalized to the CFUs count of the untreated control of each strain. Experiments were repeated at least 4 times this time, to make sure that the phenotype we observed was not an artefact. In our previous studies, we demonstrated the anti-mycobacterial activity of Spm and determined its MIC after extended exposure (≥7 days, MIC~320 μM; Sao Emani and Reiling, 2023). In this study, exposure of M.tb to 2 mM Spm for just 3 h is not enough to elicit killing of the wild-type. However, we found that as opposed to the ∆*rv0191* mutant and the previously reported ∆*rv1878* mutant (Krysenko et al., 2023), the ∆*rv1877* mutant was significantly sensitive (*p* < 0.05) to Spm stress in Sauton’s media (Figures 2A,B). Furthermore, this phenotype was completely and significantly reversed in the complemented strain of ∆*rv1877* mutant (Figure 2A, compared to the mutant, *p* < 0.01). The deletion of each gene in the mutants shown in this study was unmarked, and in-frame, because we wanted to avoid any polar effect caused by the replacement of a gene with an antibiotic cassette in case of marked mutants (Borgers et al., 2020). However, it is still possible that unmarked deletions can also cause polar effects, if the deletion construct was not properly designed to ensure an in-frame deletion or if the gene of interest carries regulatory elements required for the expression of downstream genes. Nevertheless, we also made sure that this scenario was avoided during our design (Supplementary Table S1) by allowing 50–150 bp upstream and downstream each deleted region, to avoid cropping any overlapping gene. We also made sure that the reading frame during translation of the cropped genomic region is not altered, by deleting an exact number of bases that is divisible by 3, since each amino acid is translated from 3 bases (ATDBio, 2024; Supplementary Table S1). To further confirm that this phenotype was not related to any polar effect on downstream genes caused by the deletion of *rv1877*, we evaluated the expression level (by RT-PCR) of each gene of the genomic region of *rv1876-rv1879*, and the expression of *rv0191* in the mutants and the wild-type (Supplementary Figures S3A–E). We observed that expressions of downstream genes *rv1878* and *rv1879* were not downregulated in the ∆*rv1877* mutant (Supplementary Figures S3A–E), instead, they appeared to be upregulated, as confirmed by our RNA sequencing results (Table 1), where *rv1878* displays a higher expression in the ∆*rv1877* mutant (compared to the RT-PCR results, Supplementary Figure 3C). The fold change difference between our RT-PCR data and RNA sequencing could be due to the difference between the growth stages of samples used to derive the expression by RNA sequencing (logarithmic phase) versus the growth stages of samples we used to derive the expression by RT-PCR (late logarithmic-stationary phase) or simply because of the difference in the methods used to quantify the expression. Having confirmed that Rv1877 is able to provide minimal protection against Spm stress in Sauton’s minimal media, we further investigated if that was true, during a prolonged exposure (7 days) to lower concentrations of Spm, in a completely nutrient deprived buffer such PBS. As opposed to our previous observation with the ∆*rv1878* mutant that grew better than the wild-type under this condition (Krysenko et al., 2023), and the ∆*rv019*1 mutant in this study (Figures 2B,C), the ∆*rv1877* mutant remained sensitive to Spm stress, though only marginally in this case when compared to the wild-type, *p* ˃ 0.05, but its complement seemed to grow even better than the wild-type (Figure 2C) thereby showing an enhanced reversal of the mutant’s phenotype under this condition. This further links the phenotype of the ∆*rv1877* mutant during Spm stress to the function of the missing gene (*rv1877*). All mutants displayed a marginal (*p* ˃ 0.05) growth defect when incubated for 7 days during nutrient starvation (NS), in plain PBS (Supplementary Figure S3F), yet when a low concentration (≤250 μM) of Spm was added to the same condition, the ∆*rv1878* mutant and ∆*rv0191* mutant seem to grow better than the wild-type (Figure 2D; Krysenko et al., 2023), a phenotype that was reversed in the complement of the ∆*rv0191* mutant. This was not observed at 500 μM because that was already a lethal concentration, affecting the viability of the wild-type as well (Figures 2C,D). This suggests that at sub-lethal concentrations, these strains are able to metabolize Spm as C/N (carbon/nitrogen) source during NS while the ∆*rv1877* mutant is unable to. This further supports the role of Rv1877 in the possible export of Spm.

### Rv0191 protects *Mycobacterium tuberculosis* against oxidative stress

**Figure 3:**
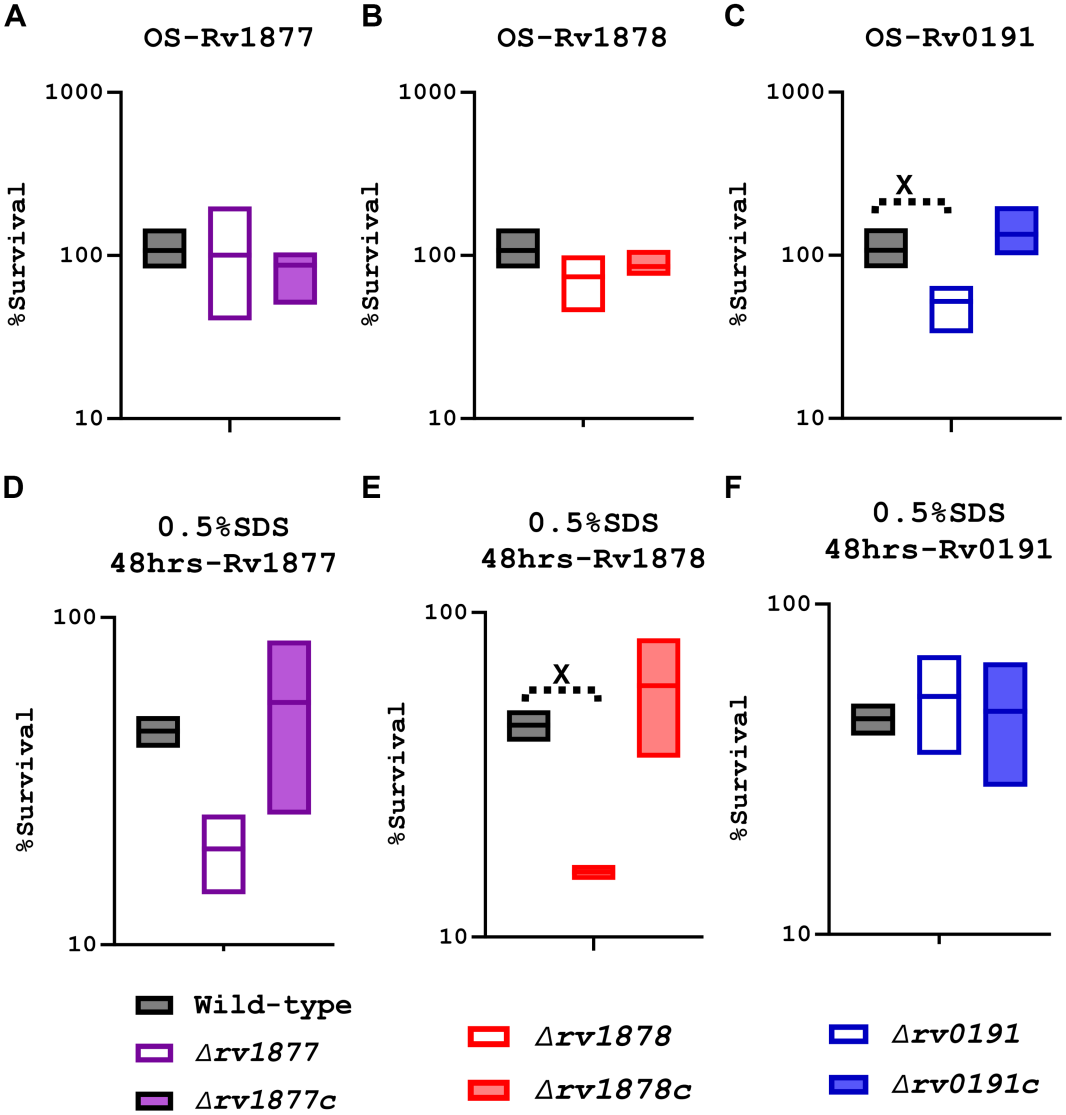
Characterization of the mutants during oxidative stress (OS) and cell wall stress (0.5% SDS). **(A)** The *∆rv1877* mutant was exposed for 3 h to 2 mM CuOOH (OS). Survival percentage was evaluated relative to the untreated DMSO control since reagent was diluted in DMSO before each experiment. The mutant displayed no sensitivity. **(B)** The *∆rv1878* mutant was exposed similarly. It displayed only a marginal sensitivity. **(C)** The *∆rv0191* mutant was exposed similarly. It displayed a statistically significant sensitivity with *p* = 0.011. **(D)** The *∆rv1877* mutant was exposed for 2 days to 0.5%SDS. The survival percentage was derived relative to the CFUs obtained from day-1. It displayed a sensitivity that was not statistically significant. **(E)** The *∆rv1878* mutant was exposed similarly. It displayed a sensitivity that was statistically significant with a *p* value of 0.028. **(F)** The *∆rv0191* mutant was exposed similarly. It displayed no sensitivity to the stress. Alpha was set to 0.05, ^*^p < 0.05, ^**^p < 0.01, ^***^p < 0.001, ^****^p < 0.0001 during the *t*-test.

M.tb encounters various hostile conditions inside macrophages during infection, such as nitrosative stress, oxidative stress (OS), cell wall stress, acidic stress, NS, iron starvation (IS) and hypoxia (Weiss and Schaible, 2015; Piacenza et al., 2019; Rankine-Wilson et al., 2021). Therefore, we aimed to study the response of the mutants in similar conditions replicated *in vitro* in order to determine the physiological role of the missing gene in each mutant. When exposed to OS [generated by cumene hydroperoxide (CuOOH), a reactive oxygen species (ROS) donor (Weiss and Estabrook, 1986b; Balvers et al., 1992)], the ∆*rv0191* mutant was the only strain that displayed a significant sensitivity (Figures 3A–C), indicating a role of Rv0191 during OS. We sought to know if *rv0191* was upregulated during OS. By using a range of concentrations of CuOOH, we investigated the expression profile of *rv0191* along with that of *rv1877*, *rv1878*, and *rv0848* (*cysK_2_*). The gene *rv0848* was previously shown to be upregulated during oxidative stress (Voskuil et al., 2011) and to be required for the protection of M.tb against OS (Sao Emani et al., 2022). As opposed to the expression of *cysK_2_* that remained high in all tested concentrations of CuOOH, the expression of *rv0191*, *rv1877* and *rv1878* was not altered (Supplementary Figure S4), indicating that the ROS detoxification role of Rv0191 is distinct to that of Rv0848. Though they both enable M.tb to survive under OS, they may use different mechanisms to achieve the same goal. Nitrosative stress also consists of exposure to free radicals, but in this case, reactive nitrogen species (RNS) instead of ROS. Some enzymes enable M.tb to survive in both nitrosative stress and OS, [EgtA and mshA for example (Sao Emani et al., 2018c)] while others are specific for only either OS (EgtD for example; Sao Emani et al., 2018c), or only nitrosative stress (Acr for example; Garbe et al., 1999; Ohno et al., 2003; Voskuil et al., 2011). Therefore, we sought to know, if the mutants generated in this study were sensitive to nitrosative stress. We exposed the mycobacteria for 3 h to the RNS-donor, tert-butyl nitrite (TBN; Liu, 2011), and found no significant difference in survival compared to the wild-type (Supplementary Figures S5A–C). To further confirm the validity of our results, we measured the expression level of the related genes during nitrosative stress, and found that it was un-altered, however, the expression of *acr* (α’-crystalline), used as our control was upregulated as previously shown (Garbe et al., 1999; Ohno et al., 2003; Voskuil et al., 2011; Supplementary Figure 5D).

### Rv1878 protects *Mycobacterium tuberculosis* against cell wall stress

The cell wall of M.tb is also key to its survival and even to its virulence within the host (Brennan, 2003). Therefore, we sought to know, if the proteins under investigation played a role in the reconstitution of the cell wall of M.tb. Since SDS is a detergent known to denature lipids and proteins (Shafa and Salton, 1960), we investigated this aspect, by exposing the mycobacteria to 0.5% SDS. The ∆*rv0191* mutant was not sensitive to cell wall stress generated by SDS, while the ∆*rv1878* was significantly sensitive (*p* < 0.05) and the ∆*rv1877* mutant was marginally sensitive (*p* ˃ 0.05; Figures 3D–F). All described phenotypes were reversed in their complements (Figure 3).

### Rv1878 and Rv1877 enable *Mycobacterium tuberculosis* to survive during hypoxia

**Figure 4:**
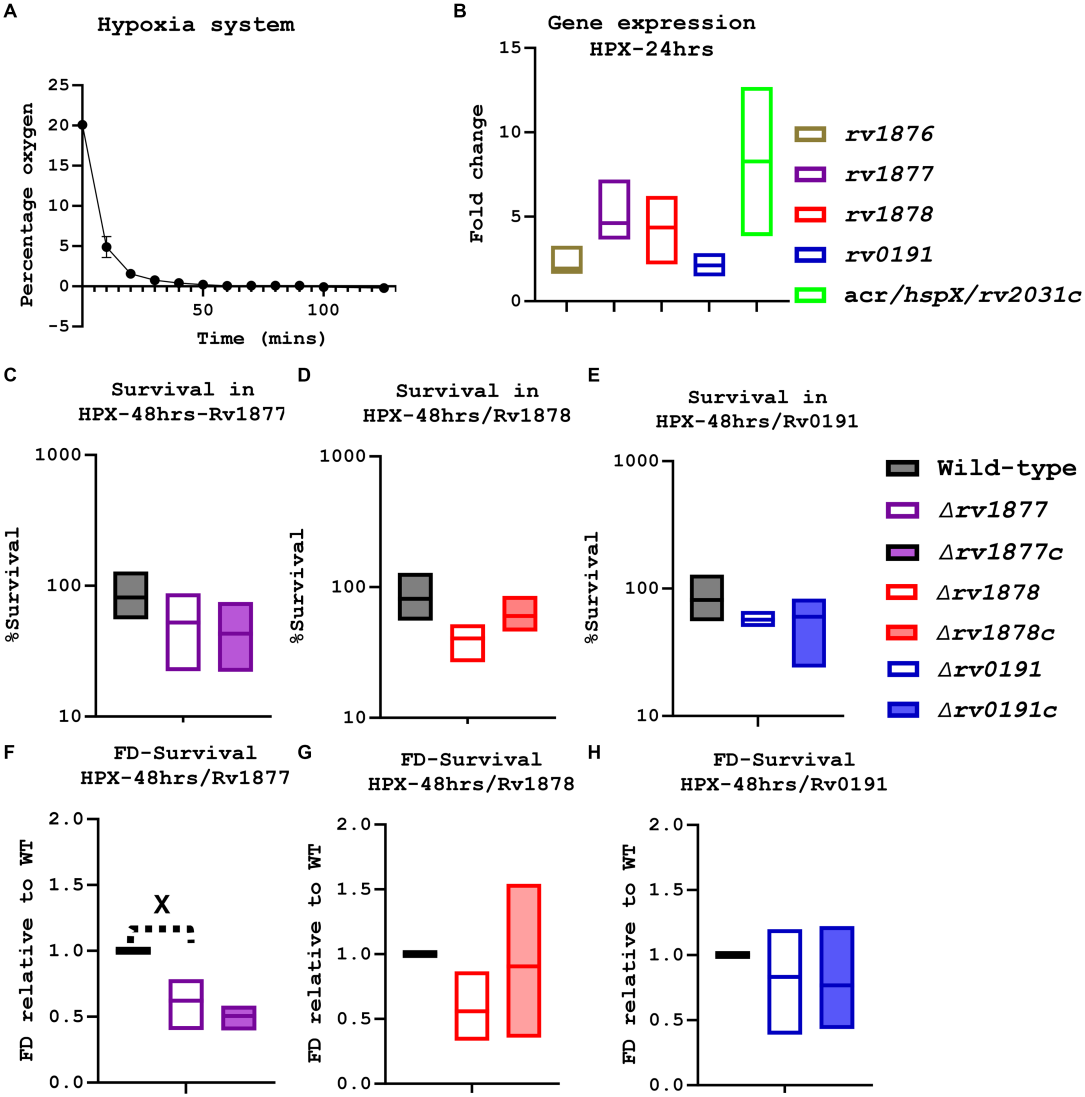
Evaluation of the physiological of the mutants during hypoxia. **(A)** An oxygen meter was placed in the hypoxia system, and the percentage of oxygen displayed on the meter was recorded every10-20 min. **(B)** Using the integrated software in the lightcycler480, the expression level of specific genes (in the anaerobic condition) was determined relative to the house-keeping gene sigA, then the absolute value was determined relative to a standard curve, finally the fold change was derived relative to the expression levels in the aerobic agitated cultures. The gene rv1877, rv1878 and acr, were marginally upregulated. **(C)** The *∆rv1877* mutant seemed not to be sensitive. **(D)** The *∆rv1878* displayed a marginal sensitivity that was partially reversed in its complement. **(E)** The *∆rv0191* mutant also displayed no sensitivity. **(F)** When the survival percentage of the mutant was normalized to the wild-type’s, the *∆rv1877* mutant displayed a statistically significant sensitivity (*p* = 0.03), but this was not complemented. **(G)** The *∆rv1878* mutant still displayed a marginal sensitivity 4 h. The *∆rv0191* mutant remained not sensitive. Alpha was set to 0.05, ^*^p < 0.05, ^**^p < 0.01, ^***^p < 0.001, ^****^p < 0.0001 during the *t*-test.

In order to determine if Rv1877, Rv1878 or Rv0191 enabled M.tb to survive during hypoxia, we made use of a hypoxia system (Anaerogen Gas Pack System, Thermo Fisher) that was previously used in an M.tb related study (Tan et al., 2010). To make sure that the system enabled depletion of oxygen to hypoxic levels, we measured the level of oxygen over time using an oxygen meter. Three independent tests revealed that oxygen was completely depleted after 3 h in the system (Figure 4A). Next, we investigated if the growth of mycobacteria could be halted in the system but yet restored as soon as they were removed from the system and exposed to oxygen (re-aerated) as observed with M.tb wild-type strains in previous hypoxic systems (Gengenbacher et al., 2010; Sherrid et al., 2010; Tizzano et al., 2021; Lin et al., 2022). This was investigated in the nutrient rich media (7H9-ADS-Tyl), in the IS and the NS media using both the fast-growing mycobacteria *M. smegmatis* and the slow-growing M.tb wild-type strain. While the growth of *M. smegmatis* was delayed but not completely halted in the system (Supplementary Figures S6A,B) the growth of M.tb was completely halted in the system (Supplementary Figure 6C), yet was restored after re-aeration of the rich media and IS media, but not in case of the NS media (Supplementary Figure 6C). The system was further validated by investigating the expression of the studied genes under the hypoxic conditions, including α’-crystallin (acr; *rv2031c*) known to be upregulated during hypoxia (Sherman et al., 2001) and *rv1876* located upstream *rv1877,* also marginally upregulated during hypoxia in previous studies (Voskuil et al., 2004). The genes *rv1877*, *rv1878* and *rv2031c* were upregulated in the system, in a range (3–5 fold; Figure 4B), similar to a previously published range observed in different hypoxia systems (Voskuil et al., 2004). However, when the sensitivity of all strains to hypoxia was evaluated by CFUs count (Figures 4C–E), the ∆*rv1878* and ∆*rv187*7 mutants displayed only a marginal (*p* ˃ 0.05) sensitivity. The ∆*rv0191* mutant was not sensitive at all, which was expected according to the expression level of *rv0191* during hypoxia (Figure 4B). When the data was analyzed differently by normalizing their survival percentage to the wild-type’s, the ∆*rv1877* mutant became statistically sensitive to hypoxia (*p* < 0.05; Figure 4F) while the ∆*rv1878* mutant remained marginally sensitive (Figure 4G) and the ∆*rv0191* mutant remained not sensitive at all (Figure 4H). These phenotypes were partially complemented (Figures 4C,D,F,G). The marginal (but not significant) phenotypes of the ∆*rv1877* and ∆*rv1878* mutants was probably due to compensation by the redundant enzymes that enable M.tb to survive in hypoxia (Voskuil et al., 2004) and/or the upregulated hypoxia related genes (*rv1738* and *rv2780*, Table 1).

### Rv1878 is involved in the regulation of iron homeostasis

**Figure 5:**
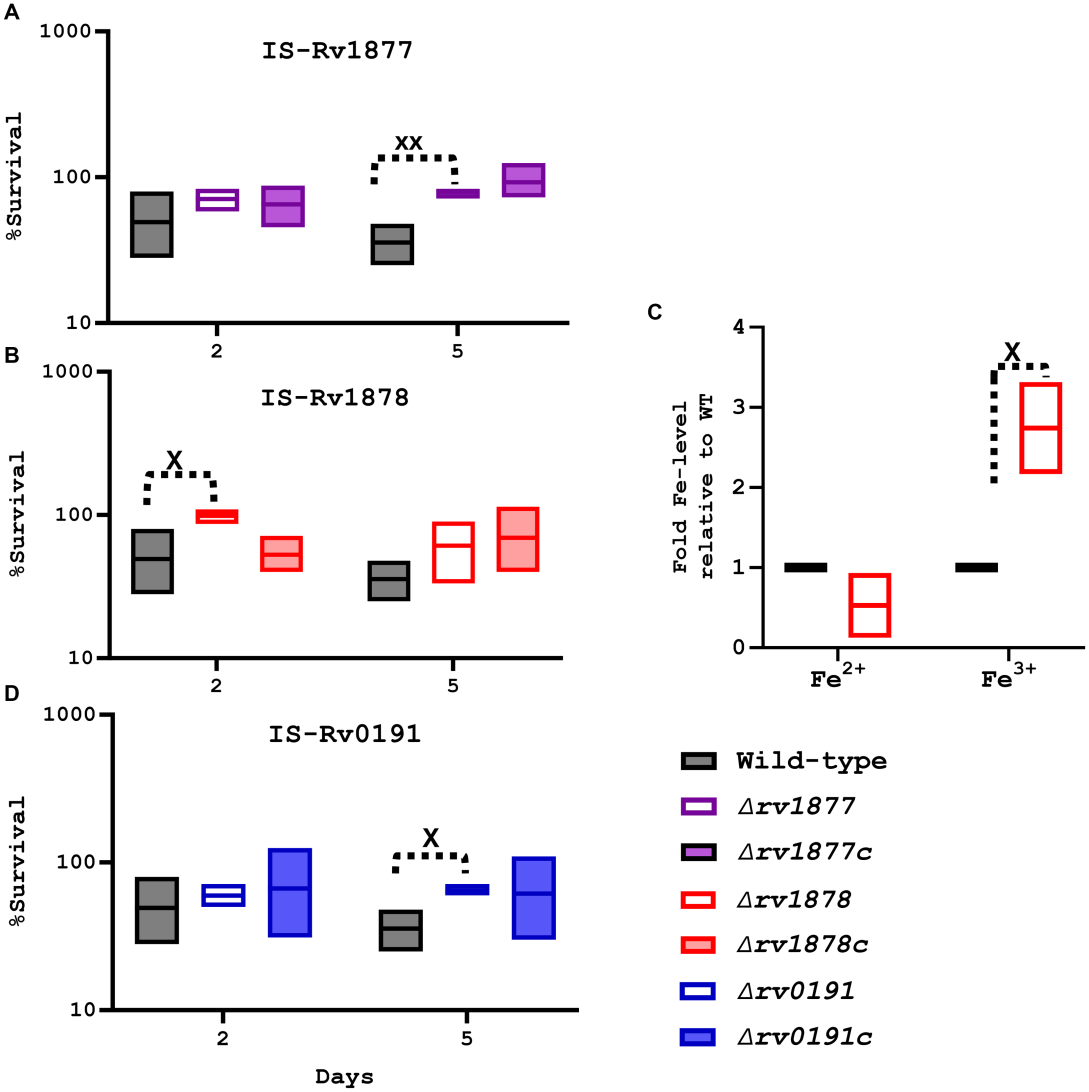
Evaluation of the physiological role of the mutants during iron starvation (IS). **(A)** The ∆rv1877 mutant was exposed for more than 2 days, to IS. Survival percentage was derived relative to CFUs obtained from day-1. It displayed a statically significant resistance at day-5 (*p* = 0.004); however, this phenotype was not reversed in its complement. **(B)** The ∆rv1878 mutant was treated similarly, and it displayed a statically resistance at day-2 (*p* = 0.04), which was reversed in its complemented strain. **(C)** The levels of ferric and ferrous irons were quantified in the wild-type and the ∆rv1878 mutant. The levels obtained in the mutants were normalized to the wild-type’s to obtain the fold Fe-levels. The mutant seemed to store more iron in ferric form, compared to the wild-type. **(D)** The ∆rv0191 exposed to IS, also displayed a statically resistance phenotype at day-5 (*p* = 0.01), which was partially reversed in its complement. Alpha was set to 0.05, ^*^p < 0.05, ^**^p < 0.01, ^***^p < 0.001, ^****^p < 0.0001 during the *t*-test.

In light of the up regulation of many genes involved in iron homeostasis in both the ∆*rv1877* and the ∆*rv1878* mutants (Table 1) and because *rv1876* located upstream of *rv*1877-*rv1878* codes for bacterioferritin (*bfrA*; Pandey and Rodriguez, 2012), that has been implicated in the regulation of iron homeostasis (Khare et al., 2017; Sharma and Bisht, 2017), we investigated the susceptibility of the mutants to IS. We found that the ∆*rv1878* was resistant to IS as it survived better than the wild-type after 2 days and 5 days (Figure 5). Though this phenotype was fully complemented after 2 days, it was not the case after 5 days (Figure 5B). This indicates that during later time points non-specific stress responses may occur, probably explaining the resistance that was observed in other strains after 5 days (Figures 5A,D) lacking a complete reversal of the phenotype in their respective complements. To further understand why the ∆*rv1878* mutant survived better in IS, we measured the level of different species of iron in the ∆*rv1878* mutant and found that it contained more ferric iron than the wild-type (Figure 5C), suggesting that it was able to store excess iron (as ferric iron) during iron abundance, which enabled it to better survive during IS.

### Rv1878 and Rv1877 enable *Mycobacterium tuberculosis* to survive during acid stress

**Figure 6:**
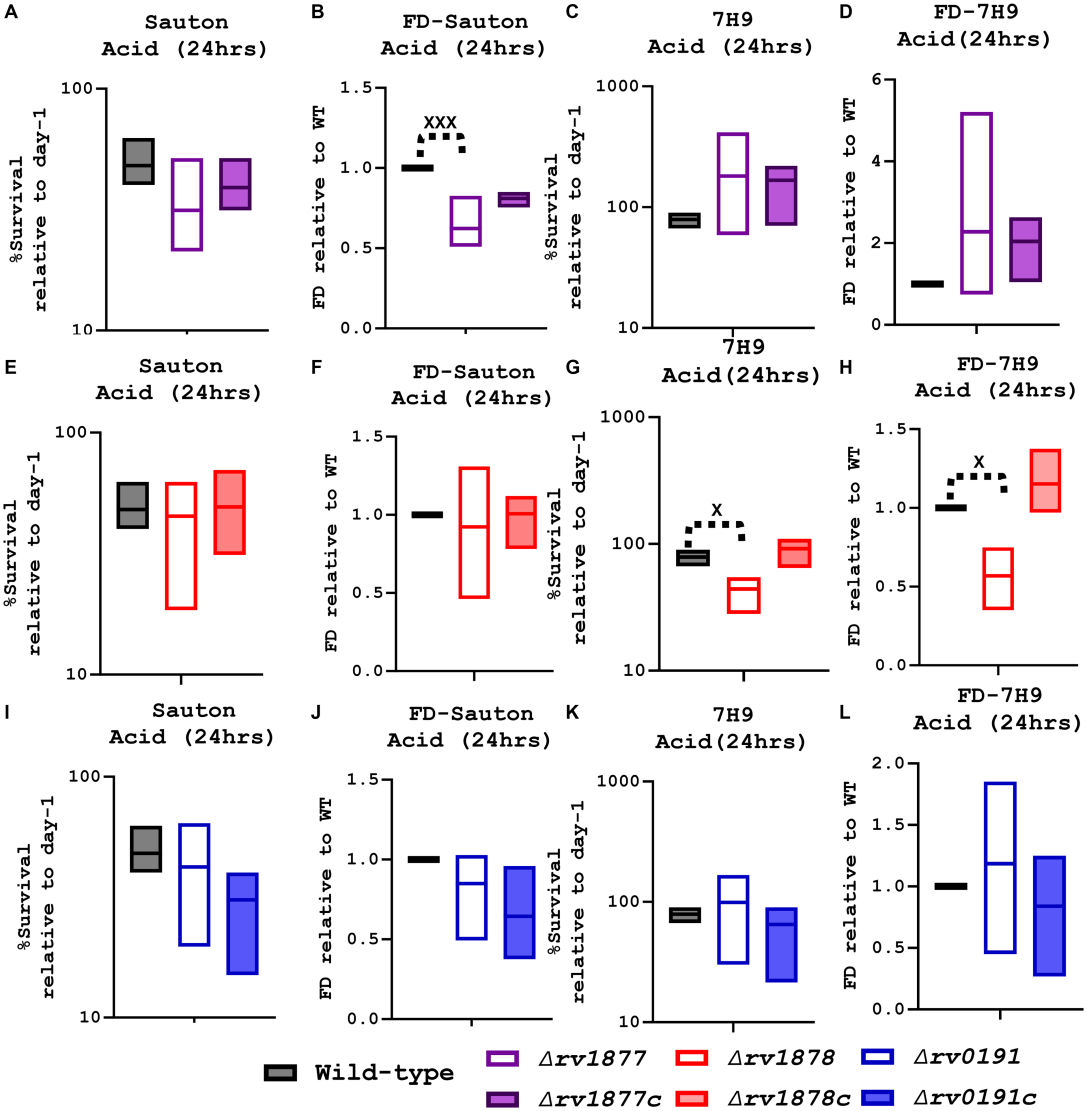
Evaluation of the survival of the mutants in acidified media. **(A)** When the ∆rv1877 mutant was exposed to acidified Sauton’s media for 24 h, it displayed a marginal sensitivity to acid stress. **(B)** For further analysis, the survival percentage of each strain was divided to the survival percentage of the wild-type to obtain the fold difference (FD), and in this case the mutant was statistically sensitive to acid stress (*p* = 0.0001). **(C)** However, it was not sensitive to acidified 7H9, 6d. Even when it was analyzed differently by using the FD. **(E)** The ∆rv1878 mutant was treated as described in 7a, and it was not sensitive to acidified Sauton’s media. **(F)** Neither was it, even when it was analyzed differently by using the FD. **(G)** However, it was significantly (*p* = 0.03) sensitive in acidified 7H9. **(H)** And also significantly sensitive (*p* = 0.0211) when it was analyzed differently by using the FD. **(I)** The ∆rv0191 was treated as described in 7a, and it was not sensitive in acidified Sauton’s media. **(J)** Neither was it, when the FD was derived. **(K)** Nor in acidified 7H9. **(L)** Also, not when data were further analyzed using the FD. Alpha was set to 0.05, ^*^p < 0.05, ^**^p < 0.01, ^***^p < 0.001, ^****^p < 0.0001during the *t*-test.

On the other hand, the mutants were also exposed to acidified media (7H9-ADS-Tyl, Sauton’s-Tyl, pH ~ 5, 24–48 h). The ∆*rv1877* mutant appeared to be sensitive (with partial complementation) when exposed for 24–48 h to acidified Sauton’s media [marginal (*p* ˃ 0.05), when the survival percentages were compared, and statistically significant (*p* < 0.001) when its survival percentage was normalized to the wild-type’s (Figures 6A–D)]. The opposite was true for the ∆*rv1878* mutant: it was not sensitive to acidified Sauton’s media, but was significantly sensitive to acidified 7H9 media when the survival percentages were compared and when its survival percentage was normalized to the wild-type’s (p < 0.05). Moreover, this phenotype was completely reversed in its complement (Figure 6). As for the ∆*rv0191* mutant, it was not sensitive to acid stress, irrespective of the media used, and the method used to analyze the data (Figures 6I–L).

## Discussion

We have recently shown that GlnA3_Mt_ (Rv1878) was not required for the detoxification of Spm in M.tb (Krysenko et al., 2023), whereas other studies showed that the corresponding *S. coelicolor* ortholog (GlnA3_Sc_) was required for the survival of this actinomycete in excess polyamines (Krysenko et al., 2017). In that same study we nevertheless found that *rv1877* encoding a multi-drug transporter (Adhikary et al., 2022) was marginally upregulated during Spm stress (Krysenko et al., 2023). Moreover, genes encoding other multi-drug transporters such as *rv3065* were significantly upregulated (Krysenko et al., 2023). Nevertheless, since we wanted to re-evaluate the physiological role of Rv1878, we generated a ∆*rv1877* mutant. This was because *rv1877* is located upstream *rv1878*, is co-transcribed with *rv1878* and other genes in the cluster (Harth et al., 2005) and they may therefore have overlapping physiological roles though not necessarily during Spm stress. Furthermore, to investigate if tolerance to Spm was facilitated by any efflux pump, or may be specific to multi-drug efflux pumps such as Rv1877 (Adhikary et al., 2022) and Rv3065 (Rodrigues et al., 2013), we generated a ∆*rv0191* mutant, where *rv0191* codes for an efflux pump which seems not be a multi-drug pump, but is more specific to chloramphenicol (Li et al., 2019). We first examined their transcriptomic profile and found that the ∆*rv1877* and ∆*rv1878* mutants displayed almost similar transcriptomic profiles (Table 1), as genes encoding enzymes involved in dormancy, hypoxia, C/N, iron and lipid metabolism were upregulated in both strains (see Results section, Supplementary Tables S3, S4), suggesting they could have overlapping functions in these physiological conditions. Overall, the mutants displayed similar transcription profiles (see Results section, Supplementary Tables S3–S5) which could be a transcription signature for all M.tb deletion mutants.

In our previous studies, we showed that Spm was bactericidal against M.tb when tested in 7H9 media, yet with a very high MIC_90_ of ~5 mM (MIC_50_ ~ 3 mM), partially due to the conjugation of Spm to albumin found in the supplement of the 7H9 media (Krysenko et al., 2023; Sao Emani and Reiling, 2023). However, it had a much lower MIC in Sauton’s media (that does not contain albumin), yet with a wide range of MIC_90_ ~ 150-320 μM (MIC_50_ ~ 80; Krysenko et al., 2023; Sao Emani and Reiling, 2023). These MICs were determined from dose response curves usually performed over 2 weeks, and the broth microdilution (resazurin) assay, which also requires incubation of mycobacteria for at least 7 days (Krysenko et al., 2023; Sao Emani and Reiling, 2023). In the current study, we exposed M.tb for only 3 h to a high concentration (2 mM) of Spm in Sauton’s media. Therefore, the toxic effect of Spm was not expected for such a short incubation, even though it was a high concentration, unless the mycobacteria had lost a protein that supposed to enable it to survive a short burst of Spm stress, which is what we found with the ∆*rv1877* mutant (Figure 2A). It was sensitive while the wild-type was not affected (Figure 2A). Suspecting that an extended exposure of the wild-type to Spm stress could affect it at concentrations ≥320 μM, we chose concentrations lower and concentrations higher than the MIC, and exposed the mycobacteria for a longer period (7 days) in a nutrient deprived buffer (PBS). As expected, the wild-type was indeed sensitive to Spm at 500 μM while it was unaffected at 250 μM, and the ∆*rv1877* mutant displayed a marginal sensitivity in this case, while its complement survived better than the wild-type (Figure 2). It is worth clarifying that the previously determined MIC_90_ used the visual scoring of the color change of resazurin in the broth microdilution assay and OD_600_ values measured over time in the dose response curves for about 2 weeks (Krysenko et al., 2023; Sao Emani and Reiling, 2023). But in this study, we used the CFUs-based method, which could explain why, though we observed loss of viability of the wild-type at a concentration (500 μM) higher than its MIC_90_, it was not a 90% loss of viability (Figure 2). This is expected when comparing different methods. In the case of the CFU-based method in this study, the mycobacteria were first exposed for 7 days, then serially diluted, and plated on 7H11 agar base plates, which were incubated for at least 2.5 weeks before the colonies were counted, giving room for the inhibited (but still viable) mycobacteria to recover, explaining the slight discrepancy between the two methods.

In our previous studies we found that the ∆*rv1878* mutant, was not sensitive to Spm stress (Krysenko et al., 2023). Instead, at lower non-toxic concentrations of Spm, it seemed to multiply despite lacking nutrients (Krysenko et al., 2023). Therefore, the physiological role of Rv1877 seems to be antagonistic to that of Rv1878 in this condition (Figure 2). Furthermore, the expression of *rv1878* is upregulated in the ∆*rv1877* mutant (Table 1; Supplementary Figure S3C). Therefore, the expression of *rv1878* is not affected by the in-frame unmarked deletion of *rv1877*, further showing that the sensitivity of the ∆*rv1877* mutant is likely not due to a polar effect on downstream genes (Supplementary Figure S3) but probably due to its non-specific efflux activity. In addition, the homologous recombination templates (inserts) of the constructs used to generate each mutant in this study were sequenced to check for integrity during each stage of the cloning process. Thus, mutation in other genes besides the genes of interest is less likely, yet is possible in some circumstances. Moreover, the reversal of this phenotype in the complemented strain of the ∆*rv1877* mutant further supports its role in Spm tolerance (Figures 2A,C).

However, it is worth noting that the difference between the wild-type and the ∆*rv1877* mutant is not more than 50% (Figure 2A, though statistically significant). It is probably due to compensation by the other multi-drug efflux pumps such as Rv3065 (Krysenko et al., 2023; and/or similar efflux pumps). It is worth noting that, *rv3065* was found to be upregulated in the ∆*rv1877* and ∆*rv1878* mutants (Table 1; Supplementary Tables S3, S4) in this study. This gene (*rv3065*) encodes a multi-drug efflux pump (Krysenko et al., 2023) that was found to be the most upregulated during Spm stress and therefore was speculated to be involved in Spm tolerance in our previous studies (Krysenko et al., 2023). Therefore, if the ∆*rv1877* mutant still displays a sensitivity towards Spm despite the upregulation of *rv3065* (that is supposed to detoxify Spm), then Rv1877 is indeed involved in the detoxification of Spm, though it is not the only protein involved (explaining the low sensitivity). Compensation can also occur by other mechanism of Spm inactivation/detoxification such as acetylation by SpeG as reported in other bacteria (Limsuwun and Jones, 2000; Filippova et al., 2019; Le et al., 2021; Kumar et al., 2022). In our previous studies, we found that M.tb has a SpeG ortholog that is Rv3034c (Sao Emani and Reiling, 2023). In this study, we discovered the upregulation of two genes that may play similar role (*rv3535c* and *rv1323* that code for enzymes possessing acetylation activities (Platt et al., 1995)) in the ∆*rv1877* mutant (Supplementary Table S3). All these findings justify the low but significant sensitivity of the ∆*rv1877* mutant to Spm stress.

It was previously shown that over-expression of *rv1877* in *E. coli* increased its resistance to a wide range of antibiotics (Adhikary et al., 2022) making it a multi-drug efflux pump, while over-expression of *rv0191* in *E. coli* increased its resistance to only chloramphenicol (Li et al., 2019; making it a more specific efflux pump). Since M.tb is naturally resistant to chloramphenicol supposedly due to the inactivating enzyme chloramphenicol acetyltransferase (CAT; Shaw, 1983; Sohaskey, 2004), it is possible that Rv0191 likely plays a more specific physiological role in M.tb. This has been observed before with other efflux pumps. A few examples include LfrA which seems to be specific for fluoroquinolones (Takiff et al., 1996) and TaP (Rv1258c) that seems to be specific for tetracycline (Ramón-García et al., 2006). On the other hand, others can detoxify a wider range of antibiotics such as the multi-drug efflux pump Mmr (Rv3065; Takiff et al., 1996; de Rossi et al., 1998; Rodrigues et al., 2013). Moreover, some efflux pumps ensure transport or secretion of endogenous metabolites such as the LpqY-SugA-SugB-SugC system that transports trehalose from the cell wall to the cytosol (Kalscheuer et al., 2010; Sharma et al., 2022) and AsnP2 (Rv0346c) that imports asparagine (Gouzy et al., 2014). Therefore, the ability of Rv1877 to enable Spm tolerance could be due to its ability to transport a wide range of compounds (de Rossi et al., 2002; Adhikary et al., 2022), as opposed to Rv0191, which has been described to be more specific, though they are both MFS-type PMF driven transporters (de Rossi et al., 2002; Li et al., 2019). It is worth noting that the gene encoding galactose kinase (galk, *rv0620*) that was found to be significantly upregulated during Spm stress in our previous studies (Krysenko et al., 2023), was significantly downregulated in the ∆*rv1877* mutant in this study (Table 2; Supplementary Tables S3). It is unclear why Spm stress will induce the expression of galK. It could be related to the effect of Spm on energy metabolism (Lüthi et al., 1999; Song et al. 2010; Sao Emani and Reiling, 2023), since galactose is imported into the cell in order to be converted through a series of reaction (one of them catalyzed by GalK) to glucose which produces ATP through glycolysis (Frey, 1996; Solopova et al., 2018). Therefore, if Rv1877 is involved in the import of galactose, the absence of Rv1877 in the corresponding mutant may explain the downregulation of galk. This suggests that Rv1877 may enable M.tb to tolerate Spm through the import of galactose, and therefore through the restoration of energy levels altered by Spm. However, this remains to be shown. On the other hand, Spm tolerance could also occur through the direct transport/export of Spm by Rv1877 and other multi-drug efflux pumps. The anti-mycobacterial activity of Spm and the ability of Spm to enhance the activity of some antibiotics (Sao Emani and Reiling, 2023) make it an attractive substrate for multi-drug efflux pumps. This is supported by previous studies that demonstrated transport of polyamines in eukaryotes by efflux pumps (Sala-Rabanal et al., 2013; Abdulhussein and Wallace, 2014; Moriyama et al., 2020). Eukaryotes are able to synthesize Spm or obtain it through their diet (Pegg, 2009). However, excess Spm can be toxic to eukaryotes (Tabor and Rosenthal, 1956), therefore the high MIC of Spm can cast doubts on the clinical relevance of this study. However, since eukaryotes can already produce Spm, it means they can tolerate a certain level of Spm, as opposed to other antibiotics. Moreover, the introduction of Spm as a food supplement to treat or prevent other disease conditions, further supports the ability of eukaryotes to tolerate an appreciable level of Spm (Senekowitsch et al., 2023). Lastly, because Spm can reduce the MIC of known TB drugs and vice versa (Sao Emani and Reiling, 2023), a high dose may not be required after all, if used in combination with these antibiotics. However, this requires further investigations *in vivo*, and in clinical settings, which is beyond the scope of this study.

Previous studies have shown that Rv1877, Rv1878 and Rv0191 are not essential for the survival of M.tb in macrophages (Rengarajan et al., 2005), nor in the *in vivo* mouse model of infection (Sassetti and Rubin, 2003;Harth et al., 2005; Lee et al., 2006). However, this could be because these systems were not suitable for the elicitation of the role of these proteins. For instance, though Rv1878 was found not to be essential for the survival of M.tb in the mouse model of infection (Harth et al., 2005; Lee et al., 2006), it was found to be essential for the survival of M.tb in the primate model of infection (Lee et al., 2006; Dutta et al., 2010). Moreover, since Spm is known to induce the polarization state of macrophages (Huang et al., 2015; Le et al., 2020), it is possible that an *in vivo* model that presents granuloma features such as the C3HeB/FeJ mice (Kramnik et al., 1998) and/or the guinea pig (Larenas-Muñoz et al., 2023) model of infection and an *ex vivo* model that uses pre or post-activated macrophages may be suitable for the study of the role of Rv1877, Rv1878 and Rv0191. However, this remains to be shown in future studies. Moreover, it was previously indicated that the ∆*rv1878* M.tb mutant was not sensitive to stress conditions such as microaerobic conditions, the utilization of different nitrogen sources, growth at different pH values and high-salt conditions (Lee et al., 2006). Here we expanded the investigation on the role of Rv1878 by characterizing the mutant in other physiological conditions, and observed that the ∆*rv1878* mutant was sensitive to acidified 7H9 and the ∆*rv1877* mutant was sensitive to acidified Sauton’s media (Figure 6). It was not indicated before at what pH values was the ∆*rv1878* mutant tested and what media was used and for how long (Lee et al., 2006). Therefore, the discrepancy between the two results could be at the type of media tested, since in this study, this mutant was not sensitive to acidified Sauton’s. Moreover, it could have been due to the difference in the exposure period to acid stress (as we observed that at later time points, this sensitivity phenotype was lost, Supplementary Figure S7). In this study, we also observed that while two genes involved in nitrogen metabolism were differentially regulated in all strains, which were *rv3012c* encoding a glutamyl-tRNA(GLN) amidotransferase-subunit C (Wolfe et al., 2010; downregulated) and *rv2780* encoding l-alanine dehydrogenase (Giffin et al., 2012; upregulated), more genes in this context were differentially regulated in the ∆*rv1877* and ∆*rv1878* mutants. These were *rv0337c* encoding an aspartate aminotransferase (AspC; Jansen et al., 2020), *rv0013* encoding a glutamine amido-transferase (Bashiri et al., 2015) and *rv3011c* encoding a glutamyl-tRNA(GLN) amidotransferase-subunit A which were downregulated in the ∆*rv1877* and ∆*rv1878* mutants (Supplementary Tables S3, S4). Moreover, few other genes involved in nitrogen metabolism were differentially regulated only in the ∆*rv1878* mutant. These were *rv1652* (*argC*), *rv1653* (*argJ*), *rv1655* (*argD*) which are all involved in the biosynthesis of arginine from glutamate (Tiwari et al., 2018) and *rv0858c* encoding N-succinyldiaminopimelate aminotransferase (DapC; Weyand et al., 2006) which were downregulated. Also, *rv3290c* (encoding lysine 6-aminotransferase; Mani Tripathi and Ramachandran, 2006) that was shown to be contribute to the ability of M.tb to persist during hypoxia (Duan et al., 2016) was upregulated only in the ∆*rv1878* mutant. Moreover, Rv1878 has been experimentally proven to be involved in nitrogen/glutamate metabolism (Harth et al., 2005). Therefore, these findings support the possibility that both Rv1877 and Rv1878 are implicated in nitrogen metabolism. And if that is the case, their phenotype during acid stress is justifiable since proteins involved in nitrogen metabolism have been implicated in the survival of M.tb during acid stress (Gouzy et al., 2014; Gallant et al., 2016). This is because of the release or consumption of ammonia (by these proteins), which is able to reduce/alter the pH of the environment (Gouzy et al., 2014; Gallant et al., 2016). It is intriguing that as opposed to the ∆*rv1878* mutant, the ∆*rv1877* mutant was sensitive to acidified Sauton’s but was not to acidified 7H9 (Figure 6). It is possible that the missing proteins in these strains play a protective role against acid stress during different environmental conditions: Rv1878, when there is abundance of nutrients (7H9) and Rv1877, when nutrients start depleting (Sauton’s) during the first 3 days of infection. Or because as opposed to 7H9, Sauton’s media does not contain glutamate, but contains asparagine as a C/N source. And the role of Rv1878 in acid stress could have been more pronounced in presence of glutamate (7H9), because it is a glutamine synthetase (Harth et al., 2005) and because few genes implicated in glutamate metabolism were downregulated in the ∆*rv1878* mutant as discussed above (Supplementary Table S4), while that of Rv1877 could have been more pronounced in the presence of asparagine (Sauton’s) because an aspartate amino transferase (*rv0337c*) is downregulated in the ∆*rv1877* mutant (Supplementary Table S3) for reasons that remained to be investigated.

On the other hand, Rv1878 seems to play a role in the cell wall modeling of M.tb since the ∆ *rv1878* mutant was sensitive to cell wall stress generated by 0.5% SDS (Figure 3). Poly-L-glutamate/glutamine cell wall structure accounts for 10% of the cell wall mass (Imaeda et al., 1968; Wietzerbin et al., 1975). Therefore, the glutamate/glutamine metabolomic function of Rv1878 (Harth et al., 2005) supports its putative role in the reconstitution of the cell wall of M.tb. Moreover, the ∆*rv1877* mutant displayed a marginal sensitivity to cell wall stress (Figure 3). However, the lack of sensitivity of the ∆*rv0191* mutant to the cell wall stress generated by SDS does not necessarily imply that it does not play any role in the cell wall reconstitution of M.tb. It could be simply be that the role of Rv0191 is not interfered by the effect of SDS. This hypothesis is supported by our previous study, where the ∆*cysk_2_* mutant was not sensitive to SDS, nor isoniazid (Sao Emani et al., 2022), yet, it was sensitive to vancomycin and displayed an altered cell wall lipid profile (Sao Emani et al., 2022). This is because vancomycin targets a lipid component (phthiocerol dimycocerosate: PDIM; Nieto and Perkins, 1971; Hammes and Neuhaus, 1974; Soetaert et al., 2015) of the cell wall of M.tb (related to the role of CysK_2_), that is different to the cell wall lipid component targeted by isoniazid (mycolate; Takayama et al., 1972; Quémard et al., 1991). Moreover, while the expression of almost no gene involved in nitrogen metabolism was altered in the ∆*rv0191* mutant, the only two genes which had an altered expression in this context in the ∆*rv0191* mutant (including other strains) were *rv3012c* which encodes a glutamyl-tRNA(GLN) amidotransferase-subunit C that was identified to be a protein of the cell wal (Wolfe et al., 2010; downregulated) and *rv2780* which encodes l-alanine dehydrogenase that catalyzes the oxidative deamination of l-alanine to pyruvate that is channeled towards the production of peptidoglycan (Giffin et al., 2012; upregulated). In addition, *rv3574* (kstR) that regulates lipid metabolism (Kendall et al., 2007) was also upregulated in all mutants while *rv0447c* encoding a cyclopropane-fatty-acyl-phospholipid synthase UfaA (Meena and Kolattukudy, 2013) was also downregulated (Supplementary Tables S3–S5) in these mutants. Moreover, the expression of *rv2911* that encodes a penicillin-binding protein (DacB2) was downregulated in all mutants and *rv3330* that encodes another penicillin-binding protein (DacB1) was downregulated in only the *rv1878* mutant (Supplementary Tables S3–S5) while penicillin itself is known to inhibit cell wall synthesis (Yocum et al., 1980). The cell envelope of M.tb consists of a plasma membrane, the myco-membrane, the cell wall and finally a capsule (Daffé et al., 2015). And the cell wall of M.tb consists of peptidoglycan, arabinoglycan, and mycolic acid forming a complex known as mycolyl-arabinogalactan-peptidoglycan (mAGP) complex (Daffé et al., 2015). In view of the finding that *rv2780* that is involved the biosynthesis of peptidoglycans (Giffin et al., 2012; a component of the cell wall; Daffé et al., 2015) was upregulated in these strains, while *rv3012c*, *rv2911* and *rv3330* that are involved in the synthesis of the cell wall (Yocum et al., 1980; Wolfe et al., 2010) were downregulated in these strains, in addition to the prior knowledge that poly-L-glutamate/glutamine cell wall structure accounts for 10% of the cell wall mass (Imaeda et al., 1968; Wietzerbin et al., 1975), these results all together suggest putative roles of Rv1877, Rv1878 and Rv0191 in the reconstitution of the cell wall of M.tb.

Furthermore, we found that the ∆*rv1878* mutant survived better in IS (Figure 5) indicating its role in the regulation of iron homeostasis. The ∆*rv1877* and ∆*rv0191* mutants displayed the same phenotype but later (5 days). However, these phenotypes were not fully complemented (only partially in the ∆*rv0191*c, Figures 5A,D). It is either because after a prolonged exposure to IS, a secondary general stress response is triggered, not necessarily related to only IS. Or, because of the general low expression of *rv1877* in its complement (Supplementary Figure S8A), or this may be related to the specific role of Rv1877 and Rv0191 during IS, they may play dual functions, enabling M.tb to survive in IS and in excess iron (EI; Figure 5D). However, this remains to be shown. The transcription profile of the mutants revealed dysregulation of many iron-homeostasis-related genes with the highest regulations observed in the ∆*rv1877* and ∆*rv1878* mutants (Table 3). A few were *rv3503c* (fdxD; Ortega Ugalde et al., 2018) an iron–sulfur cluster protein, *rv0282* encoding a type VII secretion system (Tufariello et al., 2016), and genes involved in the utilization/uptake of iron such as *rv2123* encoding PPE37 (Tullius et al., 2019) and *rv1037c* encoding an ESAT-6-like protein (EsxL; Bukka et al., 2011; Kumar et al., 2013; Jha et al., 2020), *rv3841* (*brfB*; Pandey and Rodriguez, 2012; Reddy et al., 2012; Khare et al., 2017), *rv1786* (Choi et al., 2021), *rv1177* (*fdxC*; Ortega Ugalde et al., 2018), *rv1636* (Chakraborti et al., 2021), *rv1349* (Rodriguez and Smith, 2006), *rv3597c* (*Lsr2*; Liu and Gordon, 2012), fdxD (Ortega Ugalde et al., 2018) and many others (Table 3). In addition, many other PE_PPE and PE_PGRS related genes were also differential regulated in these strains (Supplementary Table S3–S5; Table 3). However, it is worth noting that PE proteins are not all involved in the iron homeostasis of M.tb. Most are involved in the host pathogen interaction during infection, others in the secretion of other proteins, others in the regulation of apoptosis and others in the general stress response and many other physiological roles (Ates, 2020; De Maio et al., 2020). Moreover, alteration in the expression of iron-homeostasis-related gene can be induced by various stress conditions such as fatty acid synthesis inhibition, respiration inhibition, ATP synthesis inhibition, oxidative stress, DNA gyrase inhibition and others, as previously demonstrated by compounds generating these conditions (Boshoff et al., 2004). In addition, it was shown that while some PE genes were induced during IS, others were induced during EI (Gold et al., 2001; Rodriguez et al., 2002), which could explain the diversity in their regulation observed in the mutants in this study as some were upregulated while others were downregulated (Supplementary Tables S3–S5; Table 3). Therefore, relying on our RNA sequencing data alone was not enough to delineate the mechanistic roles of Rv1877, Rv1878 and Rv0191 during iron homeostasis. However, comparing our data to previously published (Gold et al., 2001; Rodriguez et al., 2002) transcriptomic profiles of M.tb during EI or IS, revealed the following. The genes *mbtA* (*rv2384*) and *rv3402c* that were previously shown to be upregulated during IS (Gold et al., 2001) are downregulated in the mutants (Table 3; Supplementary Tables S3–S5), suggesting that these mutants are not experiencing IS but the opposite, probably because they stored it in iron storage proteins such bacterioferritin, ferredoxin, since the related encoding genes were upregulated in these strains (Table 3; Supplementary Tables S3–S5). This could explain the accumulated iron levels in the ∆*rv1878* mutant (Figure 5C). Further comparison of previously published (Gold et al., 2001; Rodriguez et al., 2002) transcriptomic profile to ours, revealed that *rv2526*, *rv2549c*, *rv2550c*, *rv2927c* and *rv3246* (*mtrA*) and NADH dehydrogenase (Ndh (*rv1854c*) but not NuoA (*rv3145*) in our study) that were shown to be upregulated during EI (Rodriguez et al., 2002), are also upregulated in the mutants (Supplementary Tables S3–S5). These findings further support the theory that these mutants have high intracellular iron levels (Supplementary Tables S3–S5). Lastly, more comparison of previous findings to ours, revealed that *rv0464c*, *rv0465c*, *rv1169c*, r*v1184c*, *rv1520* and monooxygenases (*rv2378c, rv0385* and *rv0793* in our study but not specifically *rv3854c* and *rv1393c*) that were found to be downregulated during EI (Rodriguez et al., 2002), were also downregulated in the mutants of our study (Supplementary Tables S3–S5). It is worth mentioning that there were some few discrepancies (between the previous data (Rodriguez et al., 2002) and ours), but this could be because of other physiological roles of Rv1877, Rv1878 and Rv0191 that may have altered the transcription profile of the respective mutants. An example of these discrepancies was on membrane associated proteins or lipid metabolism related protein, which is reasonable since these Rv1877, Rv1878 and Rv0191 seem to be involved as well in the cell wall reconstitution of M.tb. Nevertheless, these results altogether imply that at least Rv1878 and probably Rv1877 and Rv0191 are implicated in iron homeostasis in relation to the production of iron storage proteins, though the exact mechanism remains to be shown. The study of iron homeostasis in bacteria is complex because while some enzymes enable bacteria to survive in IS, others enable them to thrive in EI and still others fulfil both functions (Khare et al., 2017; Rodriguez et al., 2022; Richardson-Sanchez et al., 2023).

**Table 3:** Iron-homeostasis and PE-related genes that were differentially regulated in the mutants.

Gene	Gene product	Regulation	Strain (FC)
*rv1983*	PE-PGRS family protein PE_PGRS35	U	∆*rv1877* (2 FC)
*rv3620c*	Putative ESAT-6 like protein EsxW (ESAT-6 like protein 10)	U	∆*rv1877*(2 FC)
*rv2107*	PE family protein PE22	U	∆*rv1877* (2 FC)
*rv2099c*	PE family protein PE21	U	∆*rv1877* (2 FC)
*rv0335c*	PE family protein PE6	D	∆*rv1877* (-5FC)
*rv1089*	PE family protein PE10	U	∆*rv1877* (2 FC)
*rv1039c*	PPE family protein PPE15	D	∆*rv1878* (−3 FC)
*rv2123*	PPE family protein PPE37	D	∆*rv1878* (−16 FC)
*rv1037c*	Putative ESAT-6 like protein EsxI (ESAT-6 like protein 1)	D	∆*rv1878* (−26 FC)
*rv0872c*	PE-PGRS family protein PE_PGRS15	U	∆*rv1878* (2 FC)
*rv3597c*	Iron-regulated H-NS-like protein Lsr2	U	∆*rv1878* (2 FC)
*rv1636*	Iron-regulated universal stress protein family protein TB15.3	U	∆*rv1877*, ∆*rv1878* (2–3 FC)
*rv1349*	Iron-regulated transporter IrtB	D	∆*rv1877*, ∆*rv1878* (−3 FC)
*rv1786*	Probable ferredoxin	U	∆*rv1877*, ∆*rv1878* (2 FC)
*rv1177*	Probable ferredoxin FdxC	U	∆*rv1877*, ∆*rv1878* (2 FC)
*rv0354c*	PPE family protein PPE7	D	∆*rv1877*, ∆*rv1878* (−2_3 FC)
*rv3875*	6 kDa early secretory antigenic target EsxA (ESAT-6)	U	∆*rv1877*, ∆*rv1878* (2 FC)
*rv2371*	PE-PGRS family protein PE_PGRS40	U	∆*rv1877*, ∆*rv1878* (2 FC)
*rv0284*	ESX conserved component EccC3. ESX-3 type VII secretion system protein. Possible membrane protein	U	∆*rv0191* (3FC)
*rv1788*	PE family protein PE18	D	∆*rv1878,* ∆*rv0191* (−2 FC)
*rv1169c*	PE family protein. Possible lipase LipX	D	∆*rv1878,* ∆*rv0191* (−2 FC)
*rv3135*	PPE family protein PPE50	D	∆*rv1878,* ∆*rv0191* (−2 FC)
*rv0096*	PPE family protein PPE1	U	∆*rv1877*, ∆*rv1878* ∆*rv0191* (5–8 FC)
*rv0280*	PPE family protein PPE3	U	∆*rv1877*, ∆*rv1878* ∆*rv0191* (5 FC)
*rv0281*	Possible S-adenosylmethionine-dependent methyltransferase	U	∆*rv1877*, ∆*rv1878* ∆*rv0191* (3 FC)
*rv0282*	ESX conserved component EccA3. ESX-3 type VII secretion system protein	U	∆*rv1877*, ∆*rv1878* ∆*rv0191* (3_6 FC)
*rv0283*	ESX conserved component EccB3. ESX-3 type VII secretion system protein. Possible membrane protein	U	∆*rv1877*, ∆*rv1878* ∆*rv0191* (3 FC)
*rv0285*	PE family protein PE5	U	∆*rv1877*, ∆*rv1878* ∆*rv0191* (3–5 FC)
*rv0286*	PPE family protein PPE4	U	∆*rv1877*, ∆*rv1878* ∆*rv0191* (3 FC)
*rv0287*	ESAT-6 like protein EsxG (conserved protein TB9.8)	U	∆*rv1877*, ∆*rv1878* ∆*rv0191* (3–4 FC)
*rv0288*	Low molecular weight protein antigen 7 EsxH (10 kDa antigen; CFP-7; protein TB10.4)	U	∆*rv1877*, ∆*rv1878* ∆*rv0191* (3–4 FC)
*rv0289*	ESX-3 secretion-associated protein EspG3	U	∆*rv1877*, ∆*rv1878* ∆*rv0191* (3 FC)
*rv3503c*	Probable ferredoxin FdxD	U	∆*rv1877*, ∆*rv1878* ∆*rv0191* (4–7 FC)
*rv3841*	Bacterioferritin BfrB	U	∆*rv1877*, ∆*rv1878* ∆*rv0191* (2_3 FC)
*rv3425*	PPE family protein PPE57	D	∆*rv1877*, ∆*rv1878* ∆*rv0191* (−2_3 FC)
*rv3746c*	Probable PE family protein PE34 (PE family-related protein)	U	∆*rv1877*, ∆*rv1878* ∆*rv0191* (4_3 FC)
*rv1088*	PE family protein PE9	U	∆*rv1877*, ∆*rv1878* ∆*rv0191* (2_4 FC)

U, upregulated; D, downregulated.

Evaluation of the transcriptomic profile of the mutants revealed many genes that displayed the same regulation across all mutants. However, genes involved the survival of M.tb during hypoxia and dormancy were mostly dysregulated in the ∆*rv1877* and ∆*rv1878* mutants (see results section, Table 1; Supplementary Tables S3–S5). Moreover, *rv1877* and *rv1878* were upregulated during hypoxia (Figure 4B) as previously shown (Voskuil et al., 2004). In line with that, we found that the ∆*rv1877* and ∆*rv1878* mutants were marginally sensitive to hypoxia (Figures 4C,D,F,G). The marginal or low sensitivity of these mutants is unlikely due to our hypoxia system, since it was validated by RT-PCR (Figure 4B) as genes previously shown to be upregulated during hypoxia were also upregulated in ours (Figure 4B). It was also validated by an oxygen meter that showed that it became hypoxic within 3 h (Figure 4A). Finally, it was also validated by the growth phenotype of mycobacteria in the system (Supplementary Figure S6). Therefore, it is possible that the upregulated hypoxia-related genes, compensated for the lack of *rv1877* or *rv1878*, making the respective mutants only marginally sensitive to hypoxia. It is worth noting that, though the ∆*rv1877* and ∆*rv1878* mutants appeared to be sensitive to hypoxia, backed up by the expression of their respective missing genes during hypoxia (Figure 4) and the dysregulation of hypoxia-related genes in their transcriptomic profile.

## Results

Table 1; Supplementary Tables S3, S4, solidifying at least a marginal role of these proteins (Rv1877 and Rv1878) during hypoxia, the phenotypes of the mutants were not fully complemented (Figures 4C,D,F,G). It is either because the experimental conditions do not allow full expression of these genes under the *hsp*60 promoter as previously observed with other genes (Carroll et al., 2010; Kolbe et al., 2020) or because of the general low expression level of these genes in their complements (Supplementary Figure S8) since the expression of *rv1877* and the expression of *rv1878* were low while that of *rv0191* was ~50-fold higher in their respective complements (Supplementary Figure S8). Therefore, it is possible that *rv1877* and *rv1878* may require regulatory elements found in their genomic location to ensure optimal expression, and could explain why there was a general low/partial complementation of the ∆*rv1877* and ∆*rv1878* mutants in most (but not all) phenotypes presented in this study. This is because the plasmid (pMV306hsp., single copy integrative vector) used to complement these strains is integrated at the *attP* site of the genome of M.tb, where each gene is transcribed independently (Lee et al., 1991; Saviola and Bishai, 2004) as opposed to the wild-type where they are co-transcribed in their original genomic location (Harth et al., 2005), probably subject to necessary regulatory elements. While complementation with pMVhsp60 in the manner presented in this study works for some genes (such as *rv0191*; Supplementary Figure S8), the expression of other genes is optimal only if they are re-introduced in their genomic location or when an episomal multi-copy plasmid is used.

On the other hand, the mutants show no sensitivity to nitrosative stress generated by TBN (relative to the wild-type, Supplementary Figures S5A–C). Since *acr* was upregulated under this condition, while the corresponding missing genes of the mutants were not (Supplementary Figure S5D), it is possible that Rv1877, Rv1878 and Rv0191 do not play any role in the defense of M.tb against nitrosative stress. Nevertheless, we previously noticed and discussed that the difference in the species of free radicals generated by various ROS or RNS donors, or the difference in their half-lives and/or structure may result to different effects on mycobacteria (Sao Emani et al., 2019). Ideally, it would be more reliable to test a wide range of RNS and ROS donors, nevertheless, the up regulation of *acr* (Supplementary Figure S5D), when mycobacteria were treated with TBN, supports its suitability for TB studies investigating the physiological roles of proteins during nitrosative stress, and therefore validates these findings. Nevertheless, the best way to show the clinical relevance of the role of these proteins during nitrosative stress and OS, would be to investigate the survival of the respective mutants in specific mice with altered OS or altered nitrosative stress responses such as the gp91phox^−/−^ (OS-deficient/phagocyte oxidase-deficient mice; Adams et al., 1997) and the NOS2^−/−^ (nitrosative stress deficient/inducible nitric oxide synthase deficient-mice; Adams et al., 1997) in future studies.

The ∆*rv0191* mutant was the only mutant that was sensitive to OS (Figure 3). It is worth noting that the difference between the wild-type and the ∆*rv0191* mutant was not very large, though was statistically significant. This is possibly due to the redundant ROS-detoxification system of M.tb (Wengenack et al., 1999; Chouchane et al., 2000; Buchmeier and Fahey, 2006; Jaeger and Flohé, 2006; Rho et al., 2006; Xu et al., 2011; Nambi et al., 2015; Saini et al., 2016; Sao Emani et al., 2018b,c, 2019), resulting to compensation by other enzymes. Moreover, the transcriptomic profile of the ∆*rv0191* mutant revealed no significant alteration in the expression of genes involved in redox-homeostasis. It remains to be shown, if that is the case under OS, if the expression profile of the ∆*rv0191* would reveal higher regulation of ROS-related enzymes relative to the wild-type. If that is the case, it is possible that under standard conditions, the ∆*rv0191* mutant is not required for basic/intrinsic ROS detoxification, but it becomes important when the strain encounters external OS assault. This phenomenon was also observed in our previous study, where the ∆*cysK_2_* mutant of M.tb showed no increased levels of ROS under standard culture conditions, yet was sensitive to OS relative to the wild-type (Sao Emani et al., 2022). While, in another study, the mycothiol-deficient ∆*mshA* M.tb mutant, could not grow on agar plates that did not contain catalase (Sareen et al., 2003; Xu et al., 2011; Sao Emani et al., 2018b), indicating its requirement for basic redox homeostasis of M.tb (den Hengst and Buttner, 2008), though *mshA* (*rv0486*) is not upregulated during oxidative stress (Namouchi et al., 2016). Therefore, it is possible that some ROS-detoxification enzymes are essential to maintain a balanced redox state even under standard conditions, while other enzymes come to play only when the mycobacteria are experiencing unusual and elevated ROS assaults. This could be related to the mechanism of ROS-detoxification of the specific enzyme. In case of MshA, it is because it is the only enzyme that catalyzes the first step of mycothiol biosynthesis. Mycothiol is a low molecular weight thiol (LMWT), that is able to detoxify a wide range of ROS, RNS and other toxins, including some antibiotics (Wang et al., 2015; Sao Emani et al., 2019). In the case of CysK_2_ (Rv0848), it is thought to be because it catalyzes the synthesis of cysteine-sulfate which serves as a signaling metabolite, that is able to activate the production of other molecules, when mycobacteria encounters stress conditions (Steiner et al., 2014). In case of Rv0191, it is possible that it enables the transport of LMWT. Therefore, as seen with genes involved in LMWT biosynthesis (including *mshA*), their anti-oxidative roles are not depicted at the transcriptomic profile but at their metabolomic profile of M.tb. This could explain why, the expression of *rv0191* is not altered under OS as opposed to *cysK_2_* (Supplementary Figure S4) whose expression is altered by various stress conditions (Voskuil, 2004; Provvedi et al., 2009; Vilchèze et al., 2013; Kurthkoti et al., 2017) including when M.tb loses a gene that may affect its fitness, as seen in this study (Supplementary Tables S3–S5; Results section), because it catalyzes the synthesis of a stress signaling molecule. Therefore, since the transcriptomic profile of the ∆*rv0191* mutant under standard growth conditions (Supplementary Table S5) did not give us a hint on its mechanistic role in the defense of M.tb against OS, a targeted metabolomic profile of intracellular and extracellular LMWT, under standard and OS stress conditions coupled with a proteomic and transcriptomic profile of the ∆*rv0191* mutant during OS may shed light on the actual mechanistic role of Rv0191 during OS. The little information we could obtain that support the putative role of Rv0191 during OS, is the fact that it is located upstream a gene encoding an oxidoreductase (*rv0197*) that was shown to be upregulated during OS (Voskuil et al., 2011). Moreover, *rv0192A*, which is small gene overlapping the DS region of *rv0191* encodes for a protein that is able to indirectly interact with MshA according to the string database.^3^ The role of efflux pumps in the protection of M.tb against OS is still an underexplored field. This is because, it was believed that the defense against OS, relied solely on ROS-detoxifying enzymes and LMWT (Voskuil et al., 2011; Sao Emani et al., 2013, 2018a,d, 2019). It was only recently that it was shown that LMWT could be secreted (Sao Emani et al., 2013, 2018d), thereby indicating that M.tb has both extracellular and intracellular ROS-defense mechanisms. It was shown that *Salmonella enterica* is able to secrete siderophore products through the MacAB efflux pump as a defense mechanism against oxidative stress (Bogomolnaya et al., 2020). Therefore, it is also possible that M.tb uses efflux pumps to secrete LMWT to detoxify extracellular ROS and RNS. To the best of our knowledge, only two studies, have reported the role of efflux pumps, p55 (Rv1410c; Ramón-García et al., 2009), Rv1258c (Sun et al., 2024) during OS in M.tb. Therefore, our results further support the possible role of specific efflux pumps during oxidative stress.

## Conclusion

In brief, we have shown for the first time, that the multi-drug efflux pump Rv1877, enables M.tb to tolerate excess spermine. Furthermore, we identified a physiological role of Rv1878 during iron starvation and cell wall stress and the roles of Rv1877 and Rv1878 during hypoxia and acidic stress. Finally, we demonstrated for the first time that Rv0191 plays a role during oxidative stress.

## Data availability statement

Raw sequencing data supporting the conclusions of this article has been deposited as a collection at figshare: Sao Emani, Carine; Reiling, Norbert (2024). The efflux pumps *Rv1877* and *Rv0191* play differential roles in the protection of Mycobacterium tuberculosis against chemical stress. figshare. Collection. https://doi.org/10.6084/m9.figshare.c.7001352.v1.

## Author contributions

CS: Conceptualization, Data curation, Investigation, Methodology, Validation, Writing – original draft, Writing – review & editing. NR: Funding acquisition, Project administration, Resources, Supervision, Visualization, Writing – review & editing.

## Footnotes

^1^ https://string-db.org/network/83332.Rv2653c

^2^ https://string-db.org/network/83332.Rv1041c

^3^ https://string-db.org/cgi/network?taskId=bL9gc4LxAy86&sessionId=bwvgB7zzVX06
