# Supplementary material for: The efflux pumps Rv1877 and Rv0191 play differential roles in the protection of Mycobacterium tuberculosis against chemical stress
Source: Front Microbiol. 2024 Mar 4;15:1359188. doi: 10.3389/fmicb.2024.1359188 (PMC10956863; doi:10.3389/fmicb.2024.1359188)
Supplement: Supplementary file 1 [file Data_Sheet_1.docx]

**The efflux pumps Rv1877 and Rv0191 play differential roles in the protection of *Mycobacterium tuberculosis* against chemical stress**

**Carine Sao Emani^1*^, Norbert Reiling^1,2*^**

**^1^Microbial Interface Biology, Research Center Borstel, Leibniz Lung Center, Parkallee 1-40, Borstel, Germany.**

^2^German Center for Infection Research (DZIF), Partner Site Hamburg-Lübeck-Borstel-Riems, Borstel, Germany

*Corresponding authors:

Carine Sao Emani ([carine.emani.sao@gmail.com](mailto:carine.emani.sao@gmail.com))**,**

**Norbert Reiling (**[nreiling@fz-borstel.de](mailto:nreiling@fz-borstel.de))

## **Supplementary Table.1**

| **Supplementary Table.1: Primers used in this study** | | | | |
| --- | --- | --- | --- | --- |
| **Primer** | **Sequence** | ***RS** | **Purpose** | **Product/size** |
| **A3-USF** | GC**TGTACA**TCTCAAGCCAGGAATCGTCAT | **BsrgI** | In frame deletion of 1245bp of the 1353 bp of *rv1878* | *US= 2402 bp |
| **A3-USR** | GC**ACTAGT** CAAGCGGTGTGGCTGTCAT | **SpeI** |  |  |
| **A3-DSF** | GC**ACTAGT** TCGCGGTACGCCAGTTAGA | **SpeI** |  | DS= 2404 bp |
| **A3-DSR** | GC**AAGCTT** AACCGCGCCAACTACCTGAC | **HindIII** |  |  |
| **A3-SF** | GGTCGACTCCCTCGCAGTTT | **NA** | Screening of the ∆*rv1878* mutant |  |
| **A3-SR1** | CGATCCGGCTCAGTGTTCC | **NA** |  | **Set 1:** WT= **614bp**  **∆ = 0 bp**  **Set 2:** WT=**2251bp**  ∆ = 2251-1245= **1006bp** |
| **A3-SR2** | GCAGCAGAACCCGATCCTGT | **NA** |  |  |
| **A3-F*** | **AAGCTT**AGAAGGAGAAGTACCGATGACAGCCACACCGCTTG | **HindIII** | Complementation of ∆*rv1878* | 1383+16+6+8=1413 |
| **A3-R** | GC**GTTAAC**TTACACACTCCAAGCCATCCGG | **HpaI** |  |  |
| **1877-USF** | GC**TGTACA**TCGTCGATGACCCTGTTCG | **BsrgI** | In frame deletion of **2004** nucleotides of *rv1877* | **2416bp** |
| **1877-USR** | GC**ACTAGT** CCATATCCGCCCTCCCATC | **SpeI** |  |  |
| **1877-DSF** | GC**ACTAGT**GGTATGAAGATCTGGGCGACC | **SpeI** |  | **2459bp** |
| **1877-DSR** | GC**AAGCTT** CACCGAGAAAGTGCAGTTCC | **HindIII** |  |  |
| **1877-SF** | CGCCGTACTGCTGGAGAAA | **NA**  **NA**  **NA**  **NA**  **NA**  **NA**  **NA** | Screening of the ∆*rv1877* mutant | **Set 1:** WT=**1072bp**  Δ=**0**  **Set 2:**  Δ=2593-2004= **589bp**  WT=2593**bp** |
| **1877-SR1** | GCACACGGCAAATACTGGG |  |  |  |
| **1877-SR2** | GGACAGATCGATGCGGAGAC |  |  |  |
| **1877-F*** | **AAGCTT**AGAAGGAGAAGTACCGATGGCGGGCCCCACAGC | **HindIII** | Complementation of the ∆*rv1877* mutant | **2064+16+6+8= 2094** |
| **1877-R** | GC**GTTAAC**CTACGTTGTAGCCGCGAGTTGGCG | **HpaI** |  |  |
| **0191-USF** | GC**GGTACC**AGGGCAACTCGGGAATGTC | **kpnI** | In frame deletion of **1098** nucleotides of *rv0191* | **2452bp** |
| **0191-USR** | GC**ACTAGT** CATAGATAAAGGCCGCGCAA | **SpeI** |  |  |
| **0191-DSF** | GC**ACTAGT**AGCACTTGTTCGAGAATCCG | **SpeI** |  | **2470bp** |
| **0191-DSR** | GC**AAGCTT** CTGCATATCGACAACACCCC | **HindIII** |  |  |
| **0191-SF** | CAGCGTAATCGTGCCCTGA | **NA** | Screening of the ∆*rv0191* mutant | **Set 1:** WT=**751bp**  Δ=**0**  **Set 2:**  Δ=1395-1098= **297bp**  WT=**1395bp** |
| **0191-SR1** | TCGTGAGCACACTGACCTTGA |  |  |  |
| **0191-SR2** | CAGCGCGACATAATTGCTTTC |  |  |  |
| **0191-F*** | **AAGCTT**AGAAGGAGAAGTACCGATGACTGCCCCAACCGGAAC | **HindIII** | Complementation of the ∆*rv0191* mutant | 1242+16+6+8= **1272bp** |
| **0191-R** | GC**GTTAAC**TTAGCCGTCGCCGGGACTC | **HpaI** |  |  |
| **MvKan-F** | CGGTTGCATTCGATTCCTGT | **NA** | Screening of M.tb strains that contain the pMVhsp complementation constructs | **150bp** |
| **MvKan-F** | CCAGACTTGTTCAACAGGCC | **NA** |  |  |
| **Sequencing primers** | | | | |
| **0191-Seq-R** | TATTCGAAGGCACCGCAAG | **NA** | Sequencing of ∆*rv0191* deletion construct  The homologous recombination template |  |
| **0191-Seq-F1** | GCCGTCGGTCAGCAGTAGAA | **NA** |  |  |
| **0191-Seq-F2** | TTCTCGAACGCCTCCTTGG | **NA** |  |  |
| **0191-Seq-F3** | TGCCGAACTCAAGCGGAT | **NA** |  |  |
| **0191-Seq-F4** | CGCCTGGTTCGTTCCTGAT | **NA** |  |  |
| **0191-Seq-F5** | GGCAGGATCCGACATCGTT | **NA** |  |  |
| **0191-Seq-F6** | CAACAGCGGCAACTTCGTG | **NA** |  |  |
| **0191-Seq-F7** | GCTGCTGAATGTCGGCAAG | **NA** |  |  |
| **0191-Seq-F8** | GGTCACCTCGATCACCGTTG | **NA** |  |  |
| **MVF** | GGCCAAGACAATTGCGGA | **NA** | Sequencing of insert in pMVhsp complementation constructs |  |
| **91-seq3** | CTGCCATGAGCCTGATGT | **NA** | Sequencing of insert in *rv0191* complementation construct (primers MV-F and 0191-SR-1 were used as well) |  |
| **91-seq4** | GTCACCGATGCTGCAATC | **NA** |  |  |
| **1877-Seq-R** | TACATCCGCTGTCAGGCGT | **NA** | Sequencing of ∆*rv1877* deletion construct  The homologous recombination template |  |
| **1877-Seq-F1** | CGACGCCAACCAGGACTTT | **NA** |  |  |
| **1877-Seq-F2** | CACACGCAAGTGGCTATCGAG | **NA** |  |  |
| **1877-Seq-F3** | AGCGATTGCGGCTGCTACT | **NA** |  |  |
| **1877-Seq-F4** | CCCATAGGAGTGGTCATGCAA | **NA** |  |  |
| **1877-Seq-F5** | CAACTCGCGGCTACAACGTA | **NA** |  |  |
| **1877-Seq-F6** | GTTCGTCCGCGATGTCAAC | **NA** |  |  |
| **1877-Seq-F7** | GCACTCGACGGCATGAAGA | **NA** |  |  |
| **1877-Seq-F8** | TCTTGAACAGGTGGCCGAA | **NA** |  |  |
| **77-seq-3** | CCGGTCATCGACTACCTTG | **NA** | Sequencing of insert in *rv1877* complementation construct (primers MV-F and 1877-SR1 were used as well) |  |
| **77-seq-4** | TCGTAAACTTCCTGGACCG | **NA** |  |  |
| **A3-Seq-R** | AACGATGGTTTGGCCGGT | **NA** | Sequencing of the ∆*rv1878* deletion construct  The homologous recombination template |  |
| **A3-Seq-F1** | GCCAGGAATCGTCATGTGC | **NA** |  |  |
| **A3-Seq-F2** | CAGCACCGTTGACCAGTCGT | **NA** |  |  |
| **A3-Seq-F3** | CAGTGGCGCTGGGTTTCTT | **NA** |  |  |
| **A3-Seq-F4** | AGTGCCATCTCCGGCTGTCT | **NA** |  |  |
| **A3-Seq-F5** | TTGAAGCGACAGCCAGACC | **NA** |  |  |
| **A3-Seq-F6** | TTGATCCGCAGACTTATTGGG | **NA** |  |  |
| **A3-Seq-F7** | AATCGTGTTGCTGCACTGCT | **NA** |  |  |
| **A3-Seq-F8** | CGTTCGATGTCAAAGCGGT | **NA** |  |  |
| **A3-Seq-F9** | CCTACGACGTCCACAATCCC | **NA** |  |  |
| **78-seq-3** | GCATCGCTATCGAGCAGTT | **NA** | Sequencing of insert in *rv1878* complementation construct (primers MV-F and 1878-SR1 were used as well) |  |
| **78-seq-4** | GGAATCTATGCATGCTGGG | **NA** |  |  |
| **G17-NIL-DS-R** | GTGCCTGACTGCGTTAGCAA | **NA** | Sequencing of all final deletion constructs | 500-600bp  Flanking outside inserted US and DS |
| **NIL-LACZ-US-F1** | GCACCGCCGAAACCCTTAT | **NA** | Sequencing of all final deletion constructs |  |
| **NIL-SACB-US-F2** | GGCTGCAGGAATTCGATATCA | **NA** | Sequencing of all final deletion constructs |  |
|  |  | **NA** |  |  |
| **Reverse transcriptase PCR primers and probes** | | | | |
| **Primer** | **Sequence** | **Probe** | **Sequence (5’-Fam/ 3’-quencher)** | **Purpose** |
| 16S-RT-F | GACCACGGGATGCATGTC | 16S-probe | F-CACCCCACCAACAAGCTGATAGGC-Q | House-keeping gene to normalize RT-PCR data of each gene |
| 16S-RT-R | CCGTCGTCGCCTTGGTAG |  |  |  |
| 76-RT-F | GCTATCAACCAATACTTTCTGCACT | 76-probe | F-TCGACGAAATGCGGCACG-Q | Quantification of the expression level of *rv1876* |
| 76-RT-R | ATCCAGCAACAAGATGCGA |  |  |  |
| 77-RT-F | GCTGTACCTGGTCGTCCT | 77-probe | F-TGTTCTGCACGATGAGAACGAGCCAC-Q | Quantification of the expression level of *rv1877* |
| 77-RT-R | AAGGTCACACCCGATGTTG |  |  |  |
| 78-RT-F | GATACGCCGGACCAACACA | 78-probe | F-ATCCTGGCCTCGGCGCCA-Q | Quantification of the expression level of *rv1878* |
| 78-RT-R | ACAGAAGGTATGCCACACCG |  |  |  |
| 79-RT-F | CGACTGGCTGGTGGAG | 79-probe | F-TCGTGAGCGTGGCTGCCCG-Q | Quantification of the expression level of *rv1879* |
| 79-RT-R | GGCGCAGTATCTCGTTG |  |  |  |
| 0191-RT-F | GGCGATGATGCTGACCG | 91-probe | F-TCGGCGGGTTTGATGGGTG-Q | Quantification of the expression level of *rv0191* |
| 0191-RT-R | CTCGAACAAGTGCTGGCTAA |  |  |  |
| CysK2-RT-F | CGGAGGGATATCAAGTCACTC | K2-probe | F-CAATACCCCGGTGCTTTGGATACCT-Q | Quantification of the expression level of *rv0848* |
| CysK2-RT-R | TTCTAGCTTGGCCCAAAAT |  |  |  |
| acr-RT-F | CATTATGGTCCGCGATGGTC | Acr-probe | F-CATCAAGGCCGAGCGCACCGAGCAGAAGAA-Q | Quantification of the expression level of *rv2031* |
| acr-RT-R | GCGAACGAAGGAACCGTAC |  |  |  |
| SigA-RT-F | CAA ACA GAT CGG CAA GGT AG | SigA probe | F-CTCAACGCCGAGGAAGAGGTC-Q | House-keeping gene to normalize RT-PCR data of each gene |
| SigA-RT-R | CTC GAT CCG CTT GGC TAG |  |  |  |

**Abbreviations/Legend:** RS: restriction site (bold nucleotides), US: upstream, DS: downstream, USF: upstream forward, USR: upstream reverse, DSF: downstream forward, DSR: downstream reverse, underlined nucleotides in A3-F, 1877-F and 0191-F are the optimized ribosomal binding site (Shine Dalgarno sequence).

## **Supplementary Table.2**

**Supplementary Table 2: Strains and Plasmids used in this study**

| **Name** | **Description** | **Source/ Reference** |
| --- | --- | --- |
| **Plasmids** | | |
| pJET | Subcloning suicidal vector, Amp^r^ | ThermoFischer |
| pJET-1877US | pJET carrying the US fragment of *rv1877* | This study |
| pJET-1877DS | pJET carrying the DS fragment of *rv1877* | This study |
| pJET-1878US | pJET carrying the US fragment of *rv1878* | ^1^ |
| pJET-1878DS | pJET carrying the DS fragment of *rv1878* | ^1^ |
| pJET-0191US | pJET carrying the US fragment of *rv0191* | This study |
| pJET-0191DS | pJET carrying the DS fragment of *rv0191* | This study |
| pJET-1877 | pJET carrying the *rv1877* gene | This study |
| pJET-1878 | pJET carrying the *rv1878* gene | This study |
| pJET-0191 | pJET carrying the *rv0191* gene | This study |
| p2NIL | Cloning vector, kan^r^ | ^2^ |
| p2NIL-1877 | p2NIL vector carrying 2416bp region upstream *rv1877* and 2459bp downstream region *rv1877* | This study |
| p2NIL-1878 | p2NIL vector carrying 2416bp region upstream *rv1878* and 2459bp downstream region *rv1878* | ^1^ |
| p2NIL-0191 | p2NIL vector carrying 2416bp region upstream *rv0191* and 2459bp downstream region *rv0191* | This study |
| pGOAL17 | Plasmid carrying *lacZ* and *sacB* genes as a *Pac*I cassette; Amp^r^ | ^2^ |
| p2NIL-1877US/DS-G17 | p2NIL-1877 carrying *lacZ* and *sacB* genes as a *Pac*I cassette | This study |
| p2NIL-1878US/DS-G17 | p2NIL-1878 carrying *lacZ* and *sacB* genes as a *Pac*I cassette | ^1^ |
| p2NIL-0191US/DS-G17 | p2NIL-0191 carrying *lacZ* and *sacB* genes as a *Pac*I cassette | This study |
| pMV306hsp | *Mycobacterium* integrating shuttle vector, Kan^r^ | ^3^ |
| pMV306hsp-*rv1877* | pMV306hsp carrying *rv1877* downstream the hsp60 promoter | This study |
| pMV306hsp-*rv1878* | pMV306hsp carrying *rv1878* downstream the hsp60 promoter | ^1^ |
| pMV306hsp-*rv0191* | pMV306hsp carrying *rv0191* downstream the hsp60 promoter | This study |
| **Strains** | | |
|  |  |  |
| H37Rv | Wild-type M.tb laboratory strain used to generate the M.tb mutants in this study | Laboratory stock (ATCC 27294) |
| *M. smegmatis* (mc^2^155) | Wild-type *M.smegmatis* strain used for the initial optimization of the hypoxia system | Laboratory stock |
| *∆rv1877* | Derivative of H37Rv carrying an unmarked deletion of *rv1877* | This study |
| *∆rv1878* | Derivative of H37Rv carrying an unmarked deletion of *rv1878* | ^1^ |
| *∆rv0191* | Derivative of H37Rv carrying an unmarked deletion of *rv0191* | This study |
| *∆rv1877 attB:: pMV306hsp-rv1877* | Derivative of *∆rv1877* carrying pMV306hsp-rv1877 integrated at the attB locus; Kan^r^ | This study |
| *∆rv1878 attB:: pMV306hsp-rv1878* | Derivative of *∆rv1878* carrying pMV306hsp-rv1878 integrated at the attB locus; Kan^r^ | This study |
| *∆rv0191 attB:: pMV306hsp-rv0191* | Derivative of *∆rv0191* carrying pMV306hsp-rv0191 integrated at the attB locus; Kan^r^ | This study |
| *DH10β* | *E.coli* strain used for routine cloning experiments, phage resistant and endonuclease I (endA1) deficient. | New England Biolabs |

## **Supplementary Table.3**

| **Supplementary Table.3 Genes that are differentially regulated in the ∆*rv1877* mutant relative to the wild-type (in Sauton’s media)** | | | |
| --- | --- | --- | --- |
| **P-value** | **gene** | **gene product** | **FC** |
| 3E-33 | *rv1878* | Probable glutamine synthetase GlnA3 (glutamine synthase) (GS-I) | 21.1 |
| 6E-04 | *rv1057* | Conserved hypothetical protein | 8.4 |
| 1E-03 | *rv0096* | PPE family protein PPE1 | 8.3 |
| 2E-02 | *rv1405c* | Putative methyltransferase | 8.1 |
| 2E-02 | *rv2057c* | 50S ribosomal protein L33 RpmG1 | 6.9 |
| 1E-02 | *rv2780* | Secreted L-alanine dehydrogenase Ald (40 kDa antigen) (TB43) | 6.8 |
| 4E-02 | *rv2058c* | 50S ribosomal protein L28 RpmB2 | 6.2 |
| 1E-03 | *rv3503c* | Probable ferredoxin FdxD | 6.2 |
| 7E-03 | *rv0282* | ESX conserved component EccA3. ESX-3 type VII secretion system protein. | 5.9 |
| 2E-02 | *rv2628* | Hypothetical protein | 5.7 |
| 2E-02 | *rv1738* | Conserved protein | 5.7 |
| 4E-02 | *rv3746c* | Probable PE family protein PE34 (PE family-related protein) | 5.6 |
| 5E-03 | *rv3574* | Transcriptional regulatory protein KstR (probably TetR-family) | 5.5 |
| 8E-03 | *rv3065* | Multidrugs-transport integral membrane protein Mmr | 5.3 |
| 2E-02 | *rv0106* | Conserved hypothetical protein | 5.0 |
| 4E-02 | *rv0280* | PPE family protein PPE3 | 5.0 |
| 1E-02 | *rv0285* | PE family protein PE5 | 4.9 |
| 1E-03 | *rv0067c* | Possible transcriptional regulatory protein (possibly TetR-family) | 4.9 |
| 7E-03 | *rv1854c* | Probable NADH dehydrogenase Ndh | 4.7 |
| 3E-03 | *rv0677c* | Possible conserved membrane protein MmpS5 | 4.6 |
| 3E-03 | *rv3502c* | Probable short-chain type dehydrogenase/reductase. Possible 17-beta-hydroxysteroid dehydrogenase. | 4.5 |
| 4E-02 | *rv0195* | Possible two component transcriptional regulatory protein (probably LuxR-family) | 4.4 |
| 2E-03 | *rv1088* | PE family protein PE9 | 4.4 |
| 4E-03 | *rv2830c* | Possible antitoxin VapB22 | 4.4 |
| 2E-02 | *rv0288* | Low molecular weight protein antigen 7 EsxH (10 kDa antigen) (CFP-7) (protein TB10.4) | 4.3 |
| 9E-04 | *rv2927c* | Conserved hypothetical protein | 4.2 |
| 4E-03 | *rv1990c* | Probable transcriptional regulatory protein | 4.2 |
| 6E-03 | *rv2729c* | Probable conserved integral membrane alanine valine and leucine rich protein | 4.1 |
| 2E-02 | *rv0678* | Conserved protein | 4.1 |
| 3E-02 | *rv1403c* | Putative methyltransferase | 4.1 |
| 4E-02 | *rv2629* | Conserved protein | 4.0 |
| 6E-02 | *rv2627c* | Conserved protein | 3.9 |
| 6E-02 | *rv3130c* | Triacylglycerol synthase (diacylglycerol acyltransferase) Tgs1 | 3.9 |
| 4E-02 | *rv1996* | Universal stress protein family protein | 3.9 |
| 8E-04 | *rv1159A* | Unknown protein | 3.8 |
| 6E-02 | *rv0569* | Conserved protein | 3.7 |
| 5E-04 | *rv0116c* | Probable L%2CD-transpeptidase LdtA | 3.7 |
| 3E-02 | *rv0287* | ESAT-6 like protein EsxG (conserved protein TB9.8) | 3.7 |
| 6E-02 | *rv3133c* | Two-component transcriptional regulatory protein DevR (probably LuxR/UhpA-family) | 3.6 |
| 3E-03 | *rv1419* | Unknown protein | 3.6 |
| 7E-04 | *rv1332* | Probable transcriptional regulatory protein | 3.6 |
| 9E-03 | *rv0188* | Probable conserved transmembrane protein | 3.5 |
| 2E-02 | *rv0286* | PPE family protein PPE4 | 3.5 |
| 9E-03 | *rv2557* | Conserved protein | 3.5 |
| 1E-03 | *rv0099* | Possible fatty-acid-CoA ligase FadD10 (fatty-acid-CoA synthetase) (fatty-acid-CoA synthase) | 3.4 |
| 1E-01 | *rv0575c* | Possible oxidoreductase | 3.4 |
| 4E-03 | *rv0097* | Possible oxidoreductase | 3.4 |
| 3E-03 | *rv0757* | Possible two component system response transcriptional positive regulator PhoP | 3.4 |
| 3E-02 | *rv0289* | ESX-3 secretion-associated protein EspG3 | 3.3 |
| 7E-03 | *rv2829c* | Possible toxin VapC22 | 3.3 |
| 1E-02 | *rv0996* | Probable conserved transmembrane protein | 3.3 |
| 1E-02 | *rv1991c* | Toxin MazF6 | 3.3 |
| 3E-03 | *rv2549c* | Possible toxin VapC20 | 3.2 |
| 2E-02 | *rv3140* | Probable acyl-CoA dehydrogenase FadE23 | 3.2 |
| 1E-03 | *rv1539* | Probable lipoprotein signal peptidase LspA | 3.2 |
| 7E-02 | *rv3188* | Conserved hypothetical protein | 3.2 |
| 4E-02 | *rv0283* | ESX conserved component EccB3. ESX-3 type VII secretion system protein. Possible membrane protein. | 3.2 |
| 7E-03 | *rv1051c* | Conserved hypothetical protein | 3.1 |
| 5E-03 | *rv0088* | Possible polyketide cyclase/dehydrase | 3.1 |
| 2E-02 | *rv1461* | Conserved protein | 3.1 |
| 6E-02 | *rv2990c* | Hypothetical protein | 3.0 |
| 9E-04 | *rv3122* | Hypothetical protein | 3.0 |
| 3E-02 | *rv2816c* | Conserved hypothetical protein | 3.0 |
| 1E-02 | *rv1670* | Conserved hypothetical protein | 3.0 |
| 2E-03 | *rv0476* | Possible conserved transmembrane protein | 3.0 |
| 1E-02 | *rv3288c* | Putative protein UsfY | 3.0 |
| 7E-03 | *rv2518c* | Probable L%2CD-transpeptidase LdtB | 3.0 |
| 2E-02 | *rv1894c* | Conserved hypothetical protein | 3.0 |
| 8E-02 | *rv3139* | Probable acyl-CoA dehydrogenase FadE24 | 2.9 |
| 3E-02 | *rv1076* | Possible lipase LipU | 2.8 |
| 7E-03 | *rv1991A* | Antitoxin MazE6 | 2.8 |
| 3E-03 | *rv0095c* | Conserved hypothetical protein | 2.8 |
| 3E-03 | *rv3238c* | Probable conserved integral membrane protein | 2.7 |
| 1E-02 | *rv2497c* | Probable branched-chain keto acid dehydrogenase E1 component%2C alpha subunit BkdA | 2.7 |
| 3E-02 | *rv3061c* | Probable acyl-CoA dehydrogenase FadE22 | 2.7 |
| 8E-03 | *rv0098* | Probable fatty acyl CoA thioesterase type III FcoT | 2.7 |
| 2E-02 | *rv3504* | Probable acyl-CoA dehydrogenase FadE26 | 2.7 |
| 8E-03 | *rv2496c* | Probable branched-chain keto acid dehydrogenase E1 component%2C beta subunit BkdB | 2.7 |
| 4E-02 | *rv1577c* | Probable PhiRv1 phage protein | 2.7 |
| 3E-02 | *rv3841* | Bacterioferritin BfrB | 2.7 |
| 1E-02 | *rv1152* | Probable transcriptional regulatory protein | 2.7 |
| 8E-02 | *rv0281* | Possible S-adenosylmethionine-dependent methyltransferase | 2.7 |
| 4E-03 | *rv3010c* | Probable 6-phosphofructokinase PfkA (phosphohexokinase) (phosphofructokinase) | 2.7 |
| 5E-03 | *rv1671* | Probable membrane protein | 2.7 |
| 2E-03 | *rv0078A* | Hypothetical protein | 2.6 |
| 4E-02 | *rv1089* | PE family protein PE10 | 2.6 |
| 2E-03 | *rv3112* | Probable molybdenum cofactor biosynthesis protein D MoaD1 (molybdopterin converting factor small subunit) (molybdopterin [MPT] converting factor%2C subunit 1) | 2.6 |
| 3E-02 | *rv2651c* | Possible PhiRv2 prophage protease | 2.6 |
| 2E-02 | *rv1982A* | Possible antitoxin VapB36 | 2.6 |
| 1E-01 | *rv0274* | Conserved protein | 2.6 |
| 1E-02 | *rv3246c* | Two component sensory transduction transcriptional regulatory protein MtrA | 2.6 |
| 9E-02 | *rv3128c* | Conserved hypothetical protein | 2.6 |
| 2E-03 | *rv1766* | Conserved protein | 2.6 |
| 7E-03 | *rv3007c* | Possible oxidoreductase | 2.5 |
| 3E-02 | *rv2650c* | Possible PhiRv2 prophage protein | 2.5 |
| 1E-02 | *rv3583c* | Possible transcription factor | 2.5 |
| 1E-02 | *rv1976c* | Conserved hypothetical protein | 2.5 |
| 8E-03 | *rv0645c* | Methoxy mycolic acid synthase 1 MmaA1 (methyl mycolic acid synthase 1) (MMA1) (hydroxy mycolic acid synthase) | 2.5 |
| 2E-03 | *rv0101* | Probable peptide synthetase Nrp (peptide synthase) | 2.5 |
| 1E-02 | *rv1222* | Anti-sigma factor RseA | 2.5 |
| 3E-03 | *rv1786* | Probable ferredoxin | 2.5 |
| 3E-02 | *rv1536* | Isoleucyl-tRNA synthetase IleS | 2.4 |
| 9E-03 | *rv1019* | Probable transcriptional regulatory protein (probably TetR-family) | 2.4 |
| 3E-03 | *rv0313* | Conserved protein | 2.4 |
| 5E-02 | *rv3289c* | Possible transmembrane protein | 2.4 |
| 1E-02 | *rv1331* | Conserved hypothetical protein | 2.4 |
| 6E-02 | *rv1888c* | Possible transmembrane protein | 2.4 |
| 1E-02 | *rv1177* | Probable ferredoxin FdxC | 2.4 |
| 2E-03 | *rv2886c* | Probable resolvase | 2.4 |
| 7E-04 | *rv3766* | Hypothetical protein | 2.4 |
| 3E-04 | *rv0100* | Conserved hypothetical protein | 2.4 |
| 1E-02 | *rv3620c* | Putative ESAT-6 like protein EsxW (ESAT-6 like protein 10) | 2.3 |
| 2E-02 | *rv2664* | Hypothetical protein | 2.3 |
| 8E-03 | *rv3847* | Hypothetical protein | 2.3 |
| 2E-02 | *rv1636* | Iron-regulated universal stress protein family protein TB15.3 | 2.3 |
| 2E-02 | *rv0686* | Probable membrane protein | 2.3 |
| 1E-02 | *rv0397A* | Conserved protein | 2.3 |
| 2E-03 | *rv0103c* | Probable cation-transporter P-type ATPase B CtpB | 2.3 |
| 8E-03 | *rv3221c* | Biotinylated protein TB7.3 | 2.3 |
| 1E-02 | *rv1899c* | Possible lipoprotein LppD | 2.3 |
| 3E-02 | *rv0744c* | Possible transcriptional regulatory protein | 2.3 |
| 2E-02 | *rv0885* | Conserved hypothetical protein | 2.2 |
| 2E-02 | *rv3287c* | Anti-sigma factor RsbW (sigma negative effector) | 2.2 |
| 3E-02 | *rv1375* | Conserved hypothetical protein | 2.2 |
| 4E-02 | *rv2558* | Conserved protein | 2.2 |
| 3E-02 | *rv3008* | Hypothetical protein | 2.2 |
| 2E-02 | *rv2371* | PE-PGRS family protein PE_PGRS40 | 2.2 |
| 3E-02 | *rv2745c* | Transcriptional regulatory protein ClgR | 2.2 |
| 3E-02 | *rv3545c* | Probable cytochrome P450 125 Cyp125 | 2.2 |
| 3E-03 | *rv2204c* | Conserved protein | 2.2 |
| 2E-02 | *rv3535c* | Probable acetaldehyde dehydrogenase (acetaldehyde dehydrogenase [acetylating]) | 2.2 |
| 3E-02 | *rv2280* | Probable dehydrogenase | 2.2 |
| 8E-02 | *rv1404* | Probable transcriptional regulatory protein | 2.2 |
| 5E-03 | *rv1488* | Possible exported conserved protein | 2.2 |
| 6E-02 | *rv1832* | Probable glycine dehydrogenase GcvB (glycine decarboxylase) (glycine cleavage system P-protein) | 2.2 |
| 1E-02 | *rv1435c* | Probable conserved proline%2C glycine%2C valine-rich secreted protein | 2.2 |
| 1E-02 | *rv1451* | Probable cytochrome C oxidase assembly factor CtaB | 2.2 |
| 8E-03 | *rv2530A* | Possible antitoxin VapB39 | 2.2 |
| 7E-03 | *rv3526* | Oxygenase component of 3-ketosteroid-9-alpha-hydroxylase KshA | 2.2 |
| 6E-03 | *rv3171c* | Possible non-heme haloperoxidase Hpx | 2.2 |
| 1E-02 | *rv2107* | PE family protein PE22 | 2.1 |
| 3E-03 | *rv3142c* | Hypothetical protein | 2.1 |
| 8E-02 | *rv2390c* | Conserved hypothetical protein | 2.1 |
| 2E-02 | *rv1350* | Probable 3-oxoacyl-[acyl-carrier protein] reductase FabG2 (3-ketoacyl-acyl carrier protein reductase) | 2.1 |
| 4E-03 | *rv1983* | PE-PGRS family protein PE_PGRS35 | 2.1 |
| 3E-02 | *rv2374c* | Probable heat shock protein transcriptional repressor HrcA | 2.1 |
| 1E-02 | *rv1989c* | Hypothetical protein | 2.1 |
| 4E-03 | *rv2697c* | Probable deoxyuridine 5'-triphosphate nucleotidohydrolase Dut (dUTPase) (dUTP pyrophosphatase) (deoxyuridine 5'-triphosphatase) (dUTP diphosphatase) (deoxyuridine-triphosphatase) | 2.1 |
| 1E-03 | *rv0495c* | Conserved hypothetical protein | 2.1 |
| 7E-03 | *rv2975c* | Conserved hypothetical protein | 2.1 |
| 1E-02 | *rv3002c* | Probable acetolactate synthase (small subunit) IlvN (acetohydroxy-acid synthase) (AHAS) (ALS) | 2.1 |
| 1E-02 | *rv0257* | Conserved hypothetical protein | 2.1 |
| 7E-03 | *rv1827* | Conserved protein with FHA domain%2C GarA | 2.1 |
| 3E-02 | *rv0941c* | Conserved hypothetical protein | 2.1 |
| 5E-02 | *rv2526* | Possible antitoxin VapB17 | 2.1 |
| 1E-02 | *rv0605* | Possible resolvase | 2.1 |
| 4E-02 | *rv3875* | 6 kDa early secretory antigenic target EsxA (ESAT-6) | 2.1 |
| 1E-02 | *rv2115c* | Mycobacterial proteasome ATPase Mpa | 2.0 |
| 5E-02 | *rv2099c* | PE family protein PE21 | 2.0 |
| 2E-03 | *rv2530c* | Possible toxin VapC39. Contains PIN domain. | 2.0 |
| 3E-02 | *rv0078B* | Conserved protein | 2.0 |
| 1E-02 | *rv1129c* | Probable transcriptional regulator protein | 2.0 |
| 6E-03 | *rv1855c* | Possible oxidoreductase | 2.0 |
| 5E-02 | *rv0898c* | Conserved hypothetical protein | 2.0 |
| 3E-02 | *rv1588c* | Partial REP13E12 repeat protein | 2.0 |
| 2E-02 | *rv1323* | Probable acetyl-CoA acetyltransferase FadA4 (acetoacetyl-CoA thiolase) | 2.0 |
| 5E-03 | *rv1096* | Possible glycosyl hydrolase | -2.0 |
| 5E-02 | *rv2116* | Conserved lipoprotein LppK | -2.0 |
| 4E-03 | *rv3443c* | 50S ribosomal protein L13 RplM | -2.0 |
| 1E-02 | *rv3322c* | Possible methyltransferase | -2.0 |
| 4E-03 | *rv2075c* | Possible hypothetical exported or envelope protein | -2.0 |
| 3E-02 | *rv3899c* | Conserved hypothetical protein | -2.0 |
| 4E-03 | *rv3623* | Probable conserved lipoprotein LpqG | -2.0 |
| 2E-02 | *rv1495* | Possible toxin MazF4 | -2.0 |
| 4E-02 | *rv3384c* | Possible toxin VapC46. Contains PIN domain. | -2.0 |
| 8E-02 | *rv2331A* | Hypothetical protein | -2.0 |
| 1E-02 | *rv1520* | Probable sugar transferase | -2.0 |
| 2E-02 | *rv0062* | Possible cellulase CelA1 (endoglucanase) (endo-1%2C4-beta-glucanase) (FI-cmcase) (carboxymethyl cellulase) | -2.0 |
| 6E-02 | *rv3332* | Probable N-acetylglucosamine-6-phosphate deacetylase NagA (GlcNAc 6-P deacetylase) | -2.0 |
| 4E-03 | *rv2784c* | Probable lipoprotein LppU | -2.0 |
| 3E-03 | *rv3011c* | Probable glutamyl-tRNA(GLN) amidotransferase (subunit A) GatA (Glu-ADT subunit A) | -2.0 |
| 5E-03 | *rv2971* | Probable oxidoreductase | -2.0 |
| 3E-03 | *rv2412* | 30S ribosomal protein S20 RpsT | -2.0 |
| 1E-02 | *rv0135c* | Possible transcriptional regulatory protein | -2.0 |
| 1E-02 | *rv0989c* | Probable polyprenyl-diphosphate synthase GrcC2 (polyprenyl pyrophosphate synthetase) | -2.0 |
| 6E-03 | *rv2566* | Long conserved protein | -2.0 |
| 5E-02 | *rv3212* | Conserved alanine valine rich protein | -2.0 |
| 8E-03 | *rv0464c* | Conserved protein | -2.1 |
| 1E-02 | *rv3742c* | Possible oxidoreductase | -2.1 |
| 8E-02 | *rv1745c* | Probable isopentenyl-diphosphate delta-isomerase Idi (IPP isomerase) (isopentenyl pyrophosphate isomerase) | -2.1 |
| 4E-02 | *rv2972c* | Possible conserved membrane or exported protein | -2.1 |
| 5E-03 | *rv1365c* | Anti-anti-sigma factor RsfA (anti-sigma factor antagonist) (regulator of sigma F A) | -2.1 |
| 7E-03 | *rv0337c* | Probable aspartate aminotransferase AspC (transaminase A) (ASPAT) | -2.1 |
| 3E-02 | *rv1637c* | Conserved protein | -2.1 |
| 2E-02 | *rv1870c* | Conserved hypothetical protein | -2.1 |
| 1E-02 | *rv2560* | Probable proline and glycine rich transmembrane protein | -2.1 |
| 8E-03 | *rv0264c* | Conserved hypothetical protein | -2.1 |
| 6E-02 | *rv2341* | Probable conserved lipoprotein LppQ | -2.1 |
| 1E-02 | *rv2507* | Possible conserved proline rich membrane protein | -2.1 |
| 1E-02 | *rv1200* | Probable conserved integral membrane transport protein | -2.1 |
| 6E-04 | *rv3809c* | UDP-galactopyranose mutase Glf (UDP-GALP mutase) (NAD+-flavin adenine dinucleotide-requiring enzyme) | -2.1 |
| 3E-02 | *rv1262c* | Hypothetical hit-like protein | -2.1 |
| 1E-02 | *rv1552* | Probable fumarate reductase [flavoprotein subunit] FrdA (fumarate dehydrogenase) (fumaric hydrogenase) | -2.1 |
| 1E-02 | *rv3403c* | Hypothetical protein | -2.1 |
| 6E-02 | *rv1532c* | Conserved hypothetical protein | -2.1 |
| 3E-02 | *rv0748* | Possible antitoxin VapB31 | -2.1 |
| 6E-02 | *rv0515* | Conserved 13E12 repeat family protein | -2.1 |
| 5E-03 | *rv1167c* | Probable transcriptional regulatory protein | -2.1 |
| 3E-03 | *rv2252* | Diacylglycerol kinase | -2.1 |
| 9E-03 | *rv0432* | Periplasmic superoxide dismutase [Cu-Zn] SodC | -2.1 |
| 5E-02 | *rv0698* | Conserved hypothetical protein | -2.1 |
| 2E-02 | *rv3796* | Conserved protein | -2.1 |
| 2E-02 | *rv2964* | Probable formyltetrahydrofolate deformylase PurU (formyl-FH(4) hydrolase) | -2.1 |
| 2E-02 | *rv0749A* | Hypothetical protein (fragment) | -2.1 |
| 8E-02 | *rv2749* | Conserved protein | -2.1 |
| 3E-03 | *rv3416* | Transcriptional regulatory protein WhiB-like WhiB3. Contains [4FE-4S] cluster. | -2.1 |
| 2E-02 | *rv0040c* | Secreted proline rich protein Mtc28 (proline rich 28 kDa antigen) | -2.1 |
| 7E-02 | *rv0087* | Possible formate hydrogenase HycE (FHL) | -2.1 |
| 4E-03 | *rv0774c* | Probable conserved exported protein | -2.1 |
| 4E-02 | *rv1580c* | Probable PhiRv1 phage protein | -2.1 |
| 2E-02 | *rv2578c* | Conserved hypothetical protein | -2.1 |
| 2E-02 | *rv3489* | Unknown protein | -2.1 |
| 2E-02 | *rv2917* | Conserved hypothetical alanine and arginine rich protein | -2.1 |
| 1E-02 | *rv0437c* | Possible phosphatidylserine decarboxylase Psd (PS decarboxylase) | -2.2 |
| 8E-03 | *rv0546c* | Conserved protein | -2.2 |
| 3E-03 | *rv0627* | Possible toxin VapC5 | -2.2 |
| 3E-02 | *rv2970A* | Conserved hypothetical protein | -2.2 |
| 2E-02 | *rv3506* | Fatty-acid-CoA synthetase FadD17 (fatty-acid-CoA synthase) (fatty-acid-CoA ligase) | -2.2 |
| 6E-03 | *rv1659* | Probable argininosuccinate lyase ArgH | -2.2 |
| 6E-02 | *rv0753c* | Probable methylmalonate-semialdehyde dehydrogenase MmsA (methylmalonic acid semialdehyde dehydrogenase) (MMSDH) | -2.2 |
| 3E-03 | *rv1957* | Hypothetical protein | -2.2 |
| 1E-03 | *rv2766c* | Probable short-chain type dehydrogenase/reductase | -2.2 |
| 4E-02 | *rv2875* | Major secreted immunogenic protein Mpt70 | -2.2 |
| 2E-02 | *rv1944c* | Conserved protein | -2.2 |
| 8E-02 | *rv2655c* | Possible PhiRv2 prophage protein | -2.2 |
| 3E-02 | *rv3060c* | Probable transcriptional regulatory protein (probably GntR-family) | -2.2 |
| 3E-03 | *rv1273c* | Probable drugs-transport transmembrane ATP-binding protein ABC transporter | -2.2 |
| 5E-03 | *rv1399c* | Probable non lipolytic carboxylesterase NlhH | -2.2 |
| 9E-04 | *rv0013* | Possible anthranilate synthase component II TrpG (glutamine amidotransferase) | -2.2 |
| 2E-02 | *rv0224c* | Possible methyltransferase (methylase) | -2.2 |
| 1E-02 | *rv0217c* | Possible esterase LipW | -2.2 |
| 4E-03 | *rv3589* | Probable adenine glycosylase MutY | -2.2 |
| 2E-02 | *rv1008* | Probable deoxyribonuclease TatD (YJJV protein) | -2.2 |
| 5E-02 | *rv1246c* | Toxin RelE | -2.2 |
| 3E-02 | *rv0421c* | Conserved hypothetical protein | -2.2 |
| 2E-02 | *rv2952* | Possible methyltransferase (methylase) | -2.2 |
| 1E-03 | *rv2017* | Transcriptional regulatory protein | -2.2 |
| 9E-03 | *rv0953c* | Possible oxidoreductase | -2.2 |
| 3E-03 | *rv0184* | Conserved hypothetical protein | -2.2 |
| 9E-04 | *rv1166* | Probable conserved lipoprotein LpqW | -2.2 |
| 5E-02 | *rv3327* | Probable transposase fusion protein | -2.2 |
| 2E-02 | *rv1158c* | Conserved hypothetical ala-%2C pro-rich protein | -2.3 |
| 8E-02 | *rv2383c* | Phenyloxazoline synthase MbtB (phenyloxazoline synthetase) | -2.3 |
| 6E-03 | *rv3693* | Possible conserved membrane protein | -2.3 |
| 2E-02 | *rv2019* | Conserved protein | -2.3 |
| 9E-03 | *rv0447c* | Probable cyclopropane-fatty-acyl-phospholipid synthase UfaA1 (cyclopropane fatty acid synthase) (CFA synthase) | -2.3 |
| 4E-02 | *rv2602* | Possible toxin VapC41. Contains PIN domain. | -2.3 |
| 2E-03 | *rv1230c* | Possible membrane protein | -2.3 |
| 2E-02 | *rv0316* | Possible muconolactone isomerase | -2.3 |
| 4E-03 | *rv3268* | Conserved hypothetical protein | -2.3 |
| 6E-03 | *rv2638* | Conserved hypothetical protein | -2.3 |
| 7E-03 | *rv1179c* | Unknown protein | -2.3 |
| 3E-02 | *rv1203c* | Hypothetical protein | -2.3 |
| 3E-02 | *rv2071c* | Precorrin-3 methylase CobM (precorrin-4 C11-methyltransferase) | -2.3 |
| 1E-02 | *rv3750c* | Possible excisionase | -2.3 |
| 6E-03 | *rv0651* | 50S ribosomal protein L10 RplJ | -2.3 |
| 3E-03 | *rv0765c* | Probable oxidoreductase | -2.3 |
| 4E-03 | *rv3039c* | Probable enoyl-CoA hydratase EchA17 (crotonase) (unsatured acyl-CoA hydratase) (enoyl hydrase) | -2.3 |
| 2E-02 | *rv3076* | Conserved hypothetical protein | -2.3 |
| 3E-03 | *rv0070c* | Serine hydroxymethyltransferase GlyA2 (serine methylase 2) (SHMT 2) | -2.3 |
| 4E-03 | *rv0048c* | Possible membrane protein | -2.3 |
| 2E-02 | *rv2529* | Hypothetical protein | -2.3 |
| 8E-02 | *rv2086* | Conserved hypothetical protein | -2.3 |
| 4E-02 | *rv1268c* | Hypothetical protein | -2.3 |
| 7E-03 | *rv1516c* | Probable sugar transferase | -2.3 |
| 2E-02 | *rv3191c* | Probable transposase | -2.3 |
| 3E-03 | *rv0130* | Probable 3-hydroxyl-thioester dehydratase | -2.4 |
| 3E-02 | *rv3912* | Hypothetical alanine rich protein | -2.4 |
| 9E-02 | *rv2807* | Conserved hypothetical protein | -2.4 |
| 3E-04 | *rv3924c* | 50S ribosomal protein L34 RpmH | -2.4 |
| 7E-02 | *rv1136* | Possible enoyl-CoA hydratase | -2.4 |
| 2E-02 | *rv2378c* | Lysine-N-oxygenase MbtG (L-lysine 6-monooxygenase) (lysine N6-hydroxylase) | -2.4 |
| 5E-02 | *rv2803* | Conserved hypothetical protein | -2.4 |
| 4E-03 | *rv0544c* | Possible conserved transmembrane protein | -2.4 |
| 8E-03 | *rv2370c* | Conserved hypothetical protein | -2.4 |
| 7E-02 | *rv0596c* | Possible antitoxin VapB4 | -2.4 |
| 3E-02 | *rv0779c* | Possible conserved transmembrane protein | -2.4 |
| 3E-03 | *rv0385* | Probable monooxygenase | -2.4 |
| 2E-02 | *rv2009* | Antitoxin VapB15 | -2.4 |
| 5E-03 | *rv3479* | Possible transmembrane protein | -2.4 |
| 5E-03 | *rv2957* | Possible glycosyl transferase | -2.4 |
| 5E-03 | *rv1171* | Conserved hypothetical protein | -2.4 |
| 1E-02 | *rv3405c* | Possible transcriptional regulatory protein | -2.4 |
| 7E-03 | *rv3752c* | Possible cytidine/deoxycytidylate deaminase | -2.4 |
| 1E-03 | *rv3909* | Conserved protein | -2.5 |
| 1E-02 | *rv1252c* | Probable lipoprotein LprE | -2.5 |
| 2E-03 | *rv2486* | Probable enoyl-CoA hydratase EchA14 (enoyl hydrase) (unsaturated acyl-CoA hydratase) (crotonase) | -2.5 |
| 3E-02 | *rv2667* | Possible ATP-dependent protease ATP-binding subunit ClpC2 | -2.5 |
| 1E-02 | *rv2843* | Probable conserved transmembrane alanine rich protein | -2.5 |
| 7E-02 | *rv3366* | Probable tRNA/rRNA methylase SpoU (tRNA/rRNA methyltransferase) | -2.5 |
| 1E-02 | *rv0402c* | Probable conserved transmembrane transport protein MmpL1 | -2.5 |
| 5E-02 | *rv0815c* | Probable thiosulfate sulfurtransferase CysA2 (rhodanese-like protein) (thiosulfate cyanide transsulfurase) (thiosulfate thiotransferase) | -2.5 |
| 4E-03 | *rv1453* | Possible transcriptional activator protein | -2.5 |
| 7E-03 | *rv0960* | Possible toxin VapC9 | -2.5 |
| 2E-02 | *rv1693* | Conserved hypothetical protein | -2.5 |
| 1E-02 | *rv0414c* | Thiamine-phosphate pyrophosphorylase ThiE (TMP pyrophosphorylase) (TMP-PPASE) (thiamine-phosphate synthase) | -2.5 |
| 1E-02 | *rv1702c* | Conserved hypothetical protein | -2.6 |
| 1E-02 | *rv2290* | Probable conserved lipoprotein LppO | -2.6 |
| 2E-02 | *rv2377c* | Putative conserved protein MbtH | -2.6 |
| 2E-03 | *rv2582* | Probable peptidyl-prolyl cis-trans isomerase B PpiB (cyclophilin) (PPIase) (rotamase) (peptidylprolyl isomerase) | -2.6 |
| 3E-03 | *rv1244* | Probable lipoprotein LpqZ | -2.6 |
| 2E-03 | *rv0822c* | Conserved protein | -2.6 |
| 7E-03 | *rv1471* | Probable thioredoxin TrxB1 | -2.6 |
| 1E-02 | *rv3463* | Conserved protein | -2.6 |
| 2E-03 | *rv3145* | Probable NADH dehydrogenase I (chain A) NuoA (NADH-ubiquinone oxidoreductase chain A) | -2.6 |
| 3E-03 | *rv2700* | Possible conserved secreted alanine rich protein | -2.6 |
| 8E-03 | *rv1723* | Probable hydrolase | -2.6 |
| 5E-02 | *rv1621c* | Probable 'component linked with the assembly of cytochrome' transport transmembrane ATP-binding protein ABC transporter CydD | -2.6 |
| 3E-03 | *rv2050* | Conserved protein | -2.6 |
| 7E-02 | *rv3427c* | Possible transposase | -2.7 |
| 2E-02 | *rv1349* | Iron-regulated transporter IrtB | -2.7 |
| 1E-02 | *rv0303* | Probable dehydrogenase/reductase | -2.7 |
| 8E-02 | *rv1995* | Unknown protein | -2.7 |
| 7E-02 | *rv3845* | Hypothetical protein | -2.7 |
| 4E-03 | *rv1935c* | Possible enoyl-CoA hydratase EchA13 (enoyl hydrase) (unsaturated acyl-CoA hydratase) (crotonase) | -2.7 |
| 3E-03 | *rv2203* | Possible conserved membrane protein | -2.7 |
| 4E-03 | *rv2541* | Hypothetical alanine rich protein | -2.7 |
| 1E-02 | *rv1975* | Conserved hypothetical protein | -2.7 |
| 9E-04 | *rv1184c* | Possible exported protein | -2.7 |
| 8E-03 | *rv2331* | Hypothetical protein | -2.7 |
| 1E-03 | *rv3390* | Probable conserved lipoprotein LpqD | -2.7 |
| 2E-02 | *rv1582c* | Probable PhiRv1 phage protein | -2.8 |
| 4E-04 | *rv1895* | Possible dehydrogenase | -2.8 |
| 5E-02 | *rv0767c* | Conserved hypothetical protein | -2.8 |
| 1E-03 | *rv3911* | Possible alternative RNA polymerase sigma factor SigM | -2.8 |
| 9E-03 | *rv3425* | PPE family protein PPE57 | -2.8 |
| 7E-03 | *rv2641* | Cadmium inducible protein CadI | -2.8 |
| 5E-03 | *rv3706c* | Conserved hypothetical proline rich protein | -2.8 |
| 5E-03 | *rv0542c* | Possible O-succinylbenzoic acid--CoA ligase MenE (OSB-CoA synthetase) (O-succinylbenzoate-CoA synthase) | -2.8 |
| 3E-05 | *rv3094c* | Conserved hypothetical protein | -2.8 |
| 5E-05 | *rv1860* | Alanine and proline rich secreted protein Apa (fibronectin attachment protein) (immunogenic protein MPT32) (antigen MPT-32) (45-kDa glycoprotein) (45/47 kDa antigen) | -2.8 |
| 7E-03 | *rv1638A* | Conserved hypothetical protein | -2.9 |
| 3E-03 | *rv3195* | Conserved hypothetical protein | -2.9 |
| 1E-02 | *rv2873* | Cell surface lipoprotein Mpt83 (lipoprotein P23) | -2.9 |
| 3E-03 | *rv0653c* | Possible transcriptional regulatory protein (probably TetR-family) | -2.9 |
| 6E-03 | *rv0076c* | Probable membrane protein | -2.9 |
| 5E-03 | *rv0302* | Probable transcriptional regulatory protein (probably TetR/AcrR-family) | -2.9 |
| 8E-03 | *rv2643* | Probable arsenic-transport integral membrane protein ArsC | -2.9 |
| 6E-03 | *rv1255c* | Probable transcriptional regulatory protein | -3.0 |
| 4E-03 | *rv0354c* | PPE family protein PPE7 | -3.0 |
| 1E-03 | *rv2473* | Possible alanine and proline rich membrane protein | -3.0 |
| 7E-02 | *rv3839* | Conserved hypothetical protein | -3.0 |
| 1E-03 | *rv0191* | Probable conserved integral membrane protein | -3.0 |
| 2E-03 | *rv3717* | Conserved hypothetical protein | -3.0 |
| 2E-03 | *rv2754c* | Probable thymidylate synthase ThyX (ts) (TSase) | -3.0 |
| 5E-03 | *rv2988c* | Probable 3-isopropylmalate dehydratase (large subunit) LeuC (isopropylmalate isomerase) (alpha-IPM isomerase) (IPMI) | -3.0 |
| 1E-02 | *rv2646* | Probable integrase | -3.1 |
| 4E-02 | *rv2662* | Hypothetical protein | -3.1 |
| 4E-02 | *rv0330c* | Hypothetical protein | -3.1 |
| 2E-03 | *rv0970* | Probable conserved integral membrane protein | -3.1 |
| 1E-02 | *rv2384* | Bifunctional enzyme MbtA: salicyl-AMP ligase (SAL-AMP ligase) + salicyl-S-ArCP synthetase | -3.1 |
| 6E-03 | *rv0789c* | Hypothetical protein | -3.1 |
| 7E-04 | *rv0725c* | Conserved hypothetical protein | -3.2 |
| 9E-03 | *rv1271c* | Conserved hypothetical secreted protein | -3.2 |
| 2E-02 | *rv2307D* | Hypothetical protein | -3.2 |
| 2E-02 | *rv1999c* | Probable conserved integral membrane protein | -3.2 |
| 3E-02 | *rv0836c* | Hypothetical protein | -3.2 |
| 5E-03 | *rv1233c* | Conserved hypothetical membrane protein | -3.3 |
| 6E-03 | *rv0344c* | Probable lipoprotein LpqJ | -3.3 |
| 2E-03 | *rv1969* | Mce-family protein Mce3D | -3.3 |
| 5E-03 | *rv3833* | Transcriptional regulatory protein (probably AraC-family) | -3.3 |
| 1E-03 | *rv0104* | Conserved hypothetical protein | -3.3 |
| 2E-03 | *rv0717* | 30S ribosomal protein S14 RpsN1 | -3.3 |
| 2E-04 | *rv0923c* | Conserved hypothetical protein | -3.3 |
| 1E-03 | *rv1579c* | Probable PhiRv1 phage protein | -3.3 |
| 2E-03 | *rv3705A* | Conserved hypothetical proline rich protein | -3.3 |
| 1E-02 | *rv2647* | Hypothetical protein | -3.4 |
| 2E-02 | *rv1586c* | Probable PhiRv1 integrase | -3.5 |
| 8E-04 | *rv0322* | Probable UDP-glucose 6-dehydrogenase UdgA (UDP-GLC dehydrogenase) (UDP-GLCDH) (UDPGDH) | -3.5 |
| 5E-02 | *rv1134* | Hypothetical protein | -3.5 |
| 8E-04 | *rv3789* | GTRA family protein | -3.6 |
| 3E-04 | *rv2911* | Probable penicillin-binding protein DacB2 (D-alanyl-D-alanine carboxypeptidase) (DD-peptidase) (DD-carboxypeptidase) (PBP) (DD-transpeptidase) (serine-type D-ala-D-ala carboxypeptidase) (D-amino acid hydrolase) | -3.6 |
| 5E-04 | *rv0449c* | Conserved hypothetical protein | -3.6 |
| 1E-03 | *rv2016* | Hypothetical protein | -3.7 |
| 1E-04 | *rv0250c* | Conserved protein | -3.7 |
| 8E-03 | *rv3012c* | Probable glutamyl-tRNA(GLN) amidotransferase (subunit C) GatC (Glu-ADT subunit C) | -3.7 |
| 5E-04 | *rv2874* | Possible integral membrane C-type cytochrome biogenesis protein DipZ | -3.7 |
| 2E-03 | *rv0001* | Chromosomal replication initiator protein DnaA | -3.7 |
| 1E-02 | *rv0791c* | Conserved protein | -3.8 |
| 2E-04 | *rv1632c* | Hypothetical protein | -3.8 |
| 1E-02 | *rv2544* | Probable conserved lipoprotein LppB | -3.8 |
| 1E-03 | *rv3772* | Probable histidinol-phosphate aminotransferase HisC2 (imidazole acetol-phosphate transaminase) (imidazolylacetolphosphate aminotransferase) | -3.9 |
| 4E-03 | *rv2642* | Possible transcriptional regulatory protein (probably ArsR-family) | -3.9 |
| 9E-04 | *rv2989* | Probable transcriptional regulatory protein | -4.0 |
| 4E-02 | *rv3395A* | Probable membrane protein | -4.0 |
| 4E-02 | *rv0619* | Probable galactose-1-phosphate uridylyltransferase GalTb [second part] | -4.0 |
| 4E-05 | *rv0165c* | Probable transcriptional regulatory protein Mce1R (probably GntR-family) | -4.0 |
| 5E-04 | *rv0959A* | Possible antitoxin VapB9 | -4.0 |
| 3E-02 | *rv2656c* | Possible PhiRv2 prophage protein | -4.2 |
| 2E-02 | *rv1583c* | Probable PhiRv1 phage protein | -4.2 |
| 5E-02 | *rv3108* | Hypothetical protein | -4.2 |
| 6E-03 | *rv1620c* | Probable 'component linked with the assembly of cytochrome' transport transmembrane ATP-binding protein ABC transporter CydC | -4.3 |
| 5E-05 | *rv0331* | Possible dehydrogenase/reductase | -4.3 |
| 6E-04 | *rv2034* | ArsR repressor protein | -4.5 |
| 2E-03 | *rv3352c* | Possible oxidoreductase | -4.5 |
| 7E-02 | *rv0335c* | PE family protein PE6 | -4.7 |
| 2E-03 | *rv1081c* | Probable conserved membrane protein | -4.8 |
| 9E-03 | *rv0792c* | Probable transcriptional regulatory protein (probably GntR-family) | -4.9 |
| 1E-02 | *rv0251c* | Heat shock protein Hsp (heat-stress-induced ribosome-binding protein A) | -4.9 |
| 5E-05 | *rv2912c* | Probable transcriptional regulatory protein (probably TetR-family) | -5.3 |
| 5E-03 | *rv3269* | Conserved protein | -5.3 |
| 3E-03 | *rv0790c* | Hypothetical protein | -5.4 |
| 6E-02 | *rv0793* | Possible monooxygenase | -5.4 |
| 3E-04 | *rv3312A* | Secreted protein antigen | -5.7 |
| 7E-04 | *rv1584c* | Possible PhiRv1 phage protein | -5.9 |
| 4E-04 | *rv0614* | Conserved hypothetical protein | -6.0 |
| 3E-05 | *rv2913c* | Possible D-amino acid aminohydrolase (D-amino acid hydrolase) | -6.0 |
| 3E-02 | *rv0327c* | Possible cytochrome P450 135A1 Cyp135A1 | -6.1 |
| 3E-04 | *rv3354* | Conserved hypothetical protein | -6.4 |
| 4E-04 | *rv2963* | Probable integral membrane protein | -7.0 |
| 3E-06 | *rv0846c* | Probable oxidase | -7.6 |
| 2E-04 | *rv3167c* | Probable transcriptional regulatory protein (probably TetR-family) | -8.1 |
| 3E-05 | *rv2386c* | Isochorismate synthase MbtI | -8.2 |
| 5E-05 | *rv2545* | Possible antitoxin VapB18 | -8.4 |
| 2E-04 | *rv0849* | Probable conserved integral membrane transport protein | -9.0 |
| 2E-02 | *rv1089A* | Probable cellulase CelA2a (endo-1%2C4-beta-glucanase) (endoglucanase) (carboxymethyl cellulase) | -9.0 |
| 9E-04 | *rv3270* | Probable metal cation-transporting P-type ATPase C CtpC | -9.2 |
| 1E-04 | *rv3402c* | Conserved hypothetical protein | -9.5 |
| 6E-04 | *rv1519* | Conserved hypothetical protein | -9.9 |
| 6E-03 | *rv0850* | Putative transposase (fragment) | -11.4 |
| 2E-04 | *rv3054c* | Conserved hypothetical protein | -13.8 |
| 3E-02 | *rv0620* | Probable galactokinase GalK (galactose kinase) | -17.4 |
| 3E-05 | *rv0848* | Possible cysteine synthase a CysK2 (O-acetylserine sulfhydrylase) (O-acetylserine (thiol)-lyase) (CSASE) | -17.8 |
| 6E-04 | *rv0448c* | Conserved hypothetical protein | -30.1 |
| 7E-03 | *rv0745* | Conserved hypothetical protein | -33.2 |
| 5E-05 | *rv0186A* | Metallothionein%2C MymT | -35.6 |
| 7E-03 | *rv2653c* | Possible PhiRv2 prophage protein | -36.8 |
| 2E-05 | *rv0847* | Probable lipoprotein LpqS | -45.0 |
| 4E-05 | *rv0150c* | Conserved hypothetical protein | -95.9 |

FC: fold change, negative values are downregulated fold-change values

## **Supplementary Table.4**

| **Supplementary Table.4 Genes that are differentially regulated in the ∆*rv1878* mutant relative to the wild-type (in Sauton’s media)** | | | |
| --- | --- | --- | --- |
| **p_value** | **gene** | **gene product** | **FC** |
| 1.8E-02 | *rv1405c* | Putative methyltransferase | 7.9 |
| 1.0E-03 | *rv1057* | Conserved hypothetical protein | 7.5 |
| 6.4E-04 | *rv3503c* | Probable ferredoxin FdxD | 7.1 |
| 2.5E-02 | *rv3746c* | Probable PE family protein PE34 (PE family-related protein) | 6.4 |
| 4.8E-03 | *rv0096* | PPE family protein PPE1 | 6.1 |
| 1.7E-02 | *rv2780* | Secreted L-alanine dehydrogenase Ald (40 kDa antigen) (TB43) | 6.0 |
| 2.2E-02 | *rv2628* | Hypothetical protein | 5.9 |
| 1.6E-02 | *rv1738* | Conserved protein | 5.9 |
| 3.7E-02 | *rv2057c* | 50S ribosomal protein L33 RpmG1 | 5.9 |
| 5.4E-03 | *rv3574* | Transcriptional regulatory protein KstR (probably TetR-family) | 5.4 |
| 1.0E-02 | *rv0282* | ESX conserved component EccA3. ESX-3 type VII secretion system protein. | 5.4 |
| 9.8E-03 | *rv3065* | Multidrugs-transport integral membrane protein Mmr | 5.1 |
| 1.1E-03 | *rv0067c* | Possible transcriptional regulatory protein (possibly TetR-family) | 4.9 |
| 1.3E-02 | *rv0285* | PE family protein PE5 | 4.7 |
| 7.9E-03 | *rv1854c* | Probable NADH dehydrogenase Ndh | 4.6 |
| 4.0E-02 | *rv0195* | Possible two component transcriptional regulatory protein (probably LuxR-family) | 4.5 |
| 2.9E-03 | *rv0677c* | Possible conserved membrane protein MmpS5 | 4.5 |
| 5.4E-02 | *rv0280* | PPE family protein PPE3 | 4.5 |
| 4.2E-03 | *rv2830c* | Possible antitoxin VapB22 | 4.4 |
| 4.5E-02 | *rv2627c* | Conserved protein | 4.4 |
| 2.0E-02 | *rv0288* | Low molecular weight protein antigen 7 EsxH (10 kDa antigen) (CFP-7) (protein TB10.4) | 4.2 |
| 3.2E-02 | *rv1996* | Universal stress protein family protein | 4.2 |
| 4.5E-04 | *rv2549c* | Possible toxin VapC20 | 4.1 |
| 1.2E-03 | *rv2927c* | Conserved hypothetical protein | 4.0 |
| 1.8E-02 | *rv0678* | Conserved protein | 4.0 |
| 7.3E-03 | *rv2729c* | Probable conserved integral membrane alanine valine and leucine rich protein | 4.0 |
| 5.9E-02 | *rv3130c* | Triacylglycerol synthase (diacylglycerol acyltransferase) Tgs1 | 4.0 |
| 5.3E-03 | *rv1990c* | Probable transcriptional regulatory protein | 3.9 |
| 7.6E-03 | *rv3502c* | Probable short-chain type dehydrogenase/reductase. Possible 17-beta-hydroxysteroid dehydrogenase. | 3.9 |
| 5.8E-02 | *rv0106* | Conserved hypothetical protein | 3.8 |
| 2.1E-03 | *rv1419* | Unknown protein | 3.8 |
| 4.7E-02 | *rv2629* | Conserved protein | 3.8 |
| 8.8E-04 | *rv1159A* | Unknown protein | 3.8 |
| 6.2E-02 | *rv0569* | Conserved protein | 3.7 |
| 1.3E-02 | *rv2816c* | Conserved hypothetical protein | 3.7 |
| 5.8E-04 | *rv1332* | Probable transcriptional regulatory protein | 3.6 |
| 6.4E-03 | *rv2557* | Conserved protein | 3.6 |
| 7.3E-03 | *rv0996* | Probable conserved transmembrane protein | 3.6 |
| 5.1E-02 | *rv1403c* | Putative methyltransferase | 3.6 |
| 6.1E-02 | *rv3133c* | Two-component transcriptional regulatory protein DevR (probably LuxR/UhpA-family) | 3.5 |
| 7.1E-02 | *rv2623* | Universal stress protein family protein TB31.7 | 3.4 |
| 1.2E-02 | *rv0188* | Probable conserved transmembrane protein | 3.3 |
| 6.5E-03 | *rv3289c* | Possible transmembrane protein | 3.3 |
| 8.3E-02 | *rv0570* | Probable ribonucleoside-diphosphate reductase (large subunit) NrdZ (ribonucleotide reductase) | 3.2 |
| 6.4E-02 | *rv3188* | Conserved hypothetical protein | 3.2 |
| 5.2E-02 | *rv0287* | ESAT-6 like protein EsxG (conserved protein TB9.8) | 3.2 |
| 5.4E-03 | *rv0757* | Possible two component system response transcriptional positive regulator PhoP | 3.2 |
| 2.4E-02 | *rv1088* | PE family protein PE9 | 3.1 |
| 7.6E-04 | *rv0095c* | Conserved hypothetical protein | 3.1 |
| 2.4E-03 | *rv0116c* | Probable L%2CD-transpeptidase LdtA | 3.1 |
| 5.6E-02 | *rv2990c* | Hypothetical protein | 3.1 |
| 1.0E-02 | *rv1670* | Conserved hypothetical protein | 3.1 |
| 3.1E-02 | *rv3140* | Probable acyl-CoA dehydrogenase FadE23 | 3.1 |
| 8.9E-03 | *rv3288c* | Putative protein UsfY | 3.0 |
| 4.5E-02 | *rv0286* | PPE family protein PPE4 | 3.0 |
| 1.4E-02 | *rv2651c* | Possible PhiRv2 prophage protease | 2.9 |
| 5.5E-02 | *rv0281* | Possible S-adenosylmethionine-dependent methyltransferase | 2.9 |
| 5.7E-02 | *rv0283* | ESX conserved component EccB3. ESX-3 type VII secretion system protein. Possible membrane protein. | 2.9 |
| 3.1E-02 | *rv1461* | Conserved protein | 2.9 |
| 6.0E-02 | *rv0289* | ESX-3 secretion-associated protein EspG3 | 2.9 |
| 9.2E-02 | *rv0827c* | Metal sensor transcriptional regulator KmtR (ArsR-SmtB family) | 2.9 |
| 2.2E-02 | *rv1894c* | Conserved hypothetical protein | 2.9 |
| 3.4E-02 | *rv1076* | Possible lipase LipU | 2.8 |
| 3.4E-02 | *rv1577c* | Probable PhiRv1 phage protein | 2.8 |
| 2.0E-03 | *rv3122* | Hypothetical protein | 2.8 |
| 1.3E-02 | *rv1982A* | Possible antitoxin VapB36 | 2.8 |
| 4.4E-03 | *rv1539* | Probable lipoprotein signal peptidase LspA | 2.8 |
| 1.3E-02 | *rv2497c* | Probable branched-chain keto acid dehydrogenase E1 component%2C alpha subunit BkdA | 2.7 |
| 4.2E-03 | *rv1065* | Conserved hypothetical protein | 2.7 |
| 2.4E-02 | *rv2829c* | Possible toxin VapC22 | 2.7 |
| 3.2E-03 | *rv3010c* | Probable 6-phosphofructokinase PfkA (phosphohexokinase) (phosphofructokinase) | 2.7 |
| 2.1E-02 | *rv3504* | Probable acyl-CoA dehydrogenase FadE26 | 2.7 |
| 9.6E-03 | *rv2550c* | Possible antitoxin VapB20 | 2.7 |
| 1.0E-02 | *rv1991A* | Antitoxin MazE6 | 2.7 |
| 5.2E-03 | *rv1222* | Anti-sigma factor RseA | 2.7 |
| 9.5E-02 | *rv3139* | Probable acyl-CoA dehydrogenase FadE24 | 2.7 |
| 3.0E-02 | *rv3061c* | Probable acyl-CoA dehydrogenase FadE22 | 2.7 |
| 8.9E-02 | *rv0274* | Conserved protein | 2.7 |
| 7.8E-02 | *rv3128c* | Conserved hypothetical protein | 2.6 |
| 3.4E-02 | *rv1991c* | Toxin MazF6 | 2.6 |
| 1.5E-02 | *rv0088* | Possible polyketide cyclase/dehydrase | 2.6 |
| 1.0E-02 | *rv0099* | Possible fatty-acid-CoA ligase FadD10 (fatty-acid-CoA synthetase) (fatty-acid-CoA synthase) | 2.6 |
| 4.2E-03 | *rv1019* | Probable transcriptional regulatory protein (probably TetR-family) | 2.6 |
| 1.6E-02 | *rv2518c* | Probable L%2CD-transpeptidase LdtB | 2.6 |
| 4.4E-03 | *rv0397A* | Conserved protein | 2.6 |
| 3.0E-02 | *rv3841* | Bacterioferritin BfrB | 2.6 |
| 9.9E-03 | *rv1636* | Iron-regulated universal stress protein family protein TB15.3 | 2.6 |
| 1.1E-02 | *rv2307B* | Hypothetical glycine rich protein | 2.6 |
| 6.7E-03 | *rv1331* | Conserved hypothetical protein | 2.6 |
| 2.0E-03 | *rv1766* | Conserved protein | 2.6 |
| 8.1E-03 | *rv3875* | 6 kDa early secretory antigenic target EsxA (ESAT-6) | 2.5 |
| 3.2E-02 | *rv2650c* | Possible PhiRv2 prophage protein | 2.5 |
| 6.0E-03 | *rv3238c* | Probable conserved integral membrane protein | 2.5 |
| 4.7E-02 | *rv1888c* | Possible transmembrane protein | 2.5 |
| 9.3E-03 | *rv0476* | Possible conserved transmembrane protein | 2.5 |
| 1.8E-02 | *rv3008* | Hypothetical protein | 2.4 |
| 3.3E-02 | *rv0097* | Possible oxidoreductase | 2.4 |
| 2.0E-02 | *rv3246c* | Two component sensory transduction transcriptional regulatory protein MtrA | 2.4 |
| 1.6E-02 | *rv0898c* | Conserved hypothetical protein | 2.4 |
| 2.3E-02 | *rv1152* | Probable transcriptional regulatory protein | 2.4 |
| 5.0E-03 | *rv3221c* | Biotinylated protein TB7.3 | 2.4 |
| 1.1E-02 | *rv1177* | Probable ferredoxin FdxC | 2.4 |
| 1.4E-02 | *rv0581* | Possible antitoxin VapB26 | 2.4 |
| 6.9E-03 | *rv1899c* | Possible lipoprotein LppD | 2.3 |
| 5.5E-03 | *rv1786* | Probable ferredoxin | 2.3 |
| 1.1E-02 | *rv2371* | PE-PGRS family protein PE_PGRS40 | 2.3 |
| 1.9E-02 | *rv3583c* | Possible transcription factor | 2.3 |
| 2.6E-02 | *rv2496c* | Probable branched-chain keto acid dehydrogenase E1 component%2C beta subunit BkdB | 2.3 |
| 3.5E-03 | *rv2886c* | Probable resolvase | 2.3 |
| 7.5E-03 | *rv3112* | Probable molybdenum cofactor biosynthesis protein D MoaD1 (molybdopterin converting factor small subunit) (molybdopterin [MPT] converting factor%2C subunit 1) | 2.3 |
| 3.9E-02 | *rv1536* | Isoleucyl-tRNA synthetase IleS | 2.3 |
| 4.7E-03 | *rv0157A* | Conserved protein | 2.3 |
| 1.6E-02 | *rv3007c* | Possible oxidoreductase | 2.3 |
| 1.3E-02 | *rv1588c* | Partial REP13E12 repeat protein | 2.3 |
| 5.7E-02 | *rv3290c* | Probable L-lysine-epsilon aminotransferase Lat (L-lysine aminotransferase) (lysine 6-aminotransferase) | 2.3 |
| 3.8E-02 | *rv2558* | Conserved protein | 2.3 |
| 1.8E-02 | *rv1671* | Probable membrane protein | 2.3 |
| 7.2E-02 | *rv1994c* | Metal sensor transcriptional regulator CmtR (ArsR-SmtB family) | 2.2 |
| 6.0E-02 | *rv0810c* | Conserved hypothetical protein | 2.2 |
| 4.3E-02 | *rv2169c* | Probable conserved transmembrane protein | 2.2 |
| 1.1E-02 | *rv0078A* | Hypothetical protein | 2.2 |
| 2.5E-02 | *rv0885* | Conserved hypothetical protein | 2.2 |
| 7.7E-02 | *rv1404* | Probable transcriptional regulatory protein | 2.2 |
| 2.1E-02 | *rv3287c* | Anti-sigma factor RsbW (sigma negative effector) | 2.2 |
| 1.3E-02 | *rv3847* | Hypothetical protein | 2.2 |
| 7.7E-04 | *rv2901c* | Conserved protein | 2.2 |
| 6.7E-03 | *rv0313* | Conserved protein | 2.2 |
| 6.0E-02 | *rv1832* | Probable glycine dehydrogenase GcvB (glycine decarboxylase) (glycine cleavage system P-protein) | 2.2 |
| 3.5E-02 | *rv0744c* | Possible transcriptional regulatory protein | 2.2 |
| 2.2E-03 | *rv3766* | Hypothetical protein | 2.2 |
| 2.5E-02 | *rv0686* | Probable membrane protein | 2.2 |
| 8.0E-02 | *rv1993c* | Conserved protein | 2.2 |
| 1.5E-02 | *rv1451* | Probable cytochrome C oxidase assembly factor CtaB | 2.1 |
| 9.2E-02 | *rv0824c* | Probable acyl-[acyl-carrier protein] desaturase DesA1 (acyl-[ACP] desaturase) (stearoyl-ACP desaturase) (protein Des) | 2.1 |
| 3.5E-02 | *rv1990A* | Possible dehydrogenase (fragment) | 2.1 |
| 3.5E-03 | *rv1698* | Outer membrane protein MctB | 2.1 |
| 4.0E-02 | *rv1976c* | Conserved hypothetical protein | 2.1 |
| 7.8E-02 | *rv3016* | Probable lipoprotein LpqA | 2.1 |
| 3.3E-02 | *rv0941c* | Conserved hypothetical protein | 2.1 |
| 1.1E-02 | *rv0101* | Probable peptide synthetase Nrp (peptide synthase) | 2.1 |
| 2.0E-02 | *rv0818* | Transcriptional regulatory protein | 2.1 |
| 5.7E-03 | *rv1855c* | Possible oxidoreductase | 2.1 |
| 3.2E-02 | *rv2374c* | Probable heat shock protein transcriptional repressor HrcA | 2.0 |
| 3.2E-02 | *rv3597c* | Iron-regulated H-NS-like protein Lsr2 | 2.0 |
| 4.9E-02 | *rv3545c* | Probable cytochrome P450 125 Cyp125 | 2.0 |
| 8.5E-03 | *rv2925c* | Probable ribonuclease III Rnc (RNase III) | 2.0 |
| 1.3E-02 | *rv1596* | Probable nicotinate-nucleotide pyrophosphatase NadC | 2.0 |
| 9.3E-02 | *rv2390c* | Conserved hypothetical protein | 2.0 |
| 4.7E-02 | *rv0872c* | PE-PGRS family protein PE_PGRS15 | 2.0 |
| 5.6E-02 | *rv2526* | Possible antitoxin VapB17 | 2.0 |
| 1.2E-02 | *rv3171c* | Possible non-heme haloperoxidase Hpx | 2.0 |
| 8.8E-02 | *rv3370c* | Probable DNA polymerase III (alpha chain) DnaE2 (DNA nucleotidyltransferase) | 2.0 |
| 5.4E-03 | *rv0227c* | Probable conserved membrane protein | -2.0 |
| 4.6E-03 | *rv3584* | Possible conserved lipoprotein LpqE | -2.0 |
| 5.9E-03 | *rv2683* | Conserved protein | -2.0 |
| 2.2E-02 | *rv0544c* | Possible conserved transmembrane protein | -2.0 |
| 9.6E-03 | *rv1725c* | Conserved hypothetical protein | -2.0 |
| 3.6E-03 | *rv3011c* | Probable glutamyl-tRNA(GLN) amidotransferase (subunit A) GatA (Glu-ADT subunit A) | -2.0 |
| 7.0E-03 | *rv3330* | Probable penicillin-binding protein DacB1 (D-alanyl-D-alanine carboxypeptidase) (DD-peptidase) (DD-carboxypeptidase) (PBP) (DD-transpeptidase) (serine-type D-ala-D-ala carboxypeptidase) (D-amino acid hydrolase) | -2.0 |
| 5.0E-02 | *rv2970A* | Conserved hypothetical protein | -2.0 |
| 9.6E-03 | *rv0337c* | Probable aspartate aminotransferase AspC (transaminase A) (ASPAT) | -2.0 |
| 9.5E-02 | *rv2893* | Possible oxidoreductase | -2.0 |
| 1.5E-02 | *rv0429c* | Probable polypeptide deformylase Def (PDF) (formylmethionine deformylase) | -2.0 |
| 1.7E-02 | *rv0138* | Conserved hypothetical protein | -2.0 |
| 3.8E-03 | *rv1166* | Probable conserved lipoprotein LpqW | -2.0 |
| 6.1E-03 | *rv1968* | Mce-family protein Mce3C | -2.0 |
| 1.4E-02 | *rv2000* | Unknown protein | -2.0 |
| 1.5E-03 | *rv3668c* | Possible protease | -2.0 |
| 1.5E-02 | *rv0546c* | Conserved protein | -2.0 |
| 8.4E-02 | *rv0344c* | Probable lipoprotein LpqJ | -2.0 |
| 1.4E-02 | *rv0130* | Probable 3-hydroxyl-thioester dehydratase | -2.0 |
| 4.4E-02 | *rv1297* | Probable transcription termination factor Rho homolog | -2.0 |
| 3.6E-02 | *rv1474c* | Probable transcriptional regulatory protein | -2.0 |
| 4.0E-02 | *rv0653c* | Possible transcriptional regulatory protein (probably TetR-family) | -2.0 |
| 4.2E-02 | *rv0316* | Possible muconolactone isomerase | -2.0 |
| 5.9E-02 | *rv2071c* | Precorrin-3 methylase CobM (precorrin-4 C11-methyltransferase) | -2.1 |
| 3.3E-02 | *rv2952* | Possible methyltransferase (methylase) | -2.1 |
| 1.7E-02 | *rv1653* | Probable glutamate N-acetyltransferase ArgJ | -2.1 |
| 1.3E-02 | *rv0618* | Probable galactose-1-phosphate uridylyltransferase GalTa [first part] | -2.1 |
| 5.8E-02 | *rv0354c* | PPE family protein PPE7 | -2.1 |
| 7.6E-03 | *rv0184* | Conserved hypothetical protein | -2.1 |
| 1.8E-02 | *rv3453* | Possible conserved transmembrane protein | -2.1 |
| 1.2E-02 | *rv1552* | Probable fumarate reductase [flavoprotein subunit] FrdA (fumarate dehydrogenase) (fumaric hydrogenase) | -2.1 |
| 5.9E-04 | *rv3093c* | Hypothetical oxidoreductase | -2.1 |
| 1.0E-02 | *rv0749* | Possible toxin VapC31. Contains PIN domain. | -2.1 |
| 3.9E-02 | *rv1252c* | Probable lipoprotein LprE | -2.1 |
| 5.4E-03 | *rv1652* | Probable N-acetyl-gamma-glutamyl-phoshate reductase ArgC | -2.1 |
| 5.1E-03 | *rv2566* | Long conserved protein | -2.1 |
| 1.3E-02 | *rv0048c* | Possible membrane protein | -2.1 |
| 4.4E-02 | *rv3060c* | Probable transcriptional regulatory protein (probably GntR-family) | -2.1 |
| 1.5E-02 | *rv0062* | Possible cellulase CelA1 (endoglucanase) (endo-1%2C4-beta-glucanase) (FI-cmcase) (carboxymethyl cellulase) | -2.1 |
| 7.5E-02 | *rv3751* | Probable integrase (fragment) | -2.1 |
| 1.2E-02 | *rv0956* | Probable 5'-phosphoribosylglycinamide formyltransferase PurN (GART) (gar transformylase) (5'-phosphoribosylglycinamide transformylase) | -2.1 |
| 3.6E-03 | *rv2046* | Probable lipoprotein LppI | -2.1 |
| 1.9E-03 | *rv1710* | Possible segregation and condensation protein ScpB | -2.1 |
| 3.9E-02 | *rv0402c* | Probable conserved transmembrane transport protein MmpL1 | -2.1 |
| 2.8E-02 | *rv2069* | RNA polymerase sigma factor%2C ECF subfamily%2C SigC | -2.1 |
| 8.2E-03 | *rv2053c* | Probable transmembrane protein FxsA | -2.1 |
| 5.9E-04 | *rv3809c* | UDP-galactopyranose mutase Glf (UDP-GALP mutase) (NAD+-flavin adenine dinucleotide-requiring enzyme) | -2.1 |
| 2.8E-03 | *rv3416* | Transcriptional regulatory protein WhiB-like WhiB3. Contains [4FE-4S] cluster. | -2.1 |
| 1.4E-02 | *rv2469c* | Conserved hypothetical protein | -2.1 |
| 7.0E-02 | *rv0087* | Possible formate hydrogenase HycE (FHL) | -2.1 |
| 5.9E-02 | *rv3517* | Conserved hypothetical protein | -2.1 |
| 8.7E-03 | *rv0989c* | Probable polyprenyl-diphosphate synthase GrcC2 (polyprenyl pyrophosphate synthetase) | -2.1 |
| 8.9E-03 | *rv0070c* | Serine hydroxymethyltransferase GlyA2 (serine methylase 2) (SHMT 2) | -2.1 |
| 1.3E-02 | *rv2209* | Probable conserved integral membrane protein | -2.1 |
| 3.8E-03 | *rv2765* | Probable alanine rich hydrolase | -2.1 |
| 7.7E-03 | *rv0858c* | Probable N-succinyldiaminopimelate aminotransferase DapC (DAP-at) | -2.1 |
| 9.2E-03 | *rv3039c* | Probable enoyl-CoA hydratase EchA17 (crotonase) (unsatured acyl-CoA hydratase) (enoyl hydrase) | -2.1 |
| 1.3E-02 | *rv0428c* | GCN5-related N-acetyltransferase | -2.1 |
| 2.2E-02 | *rv1709* | Possible segregation and condensation protein ScpA | -2.1 |
| 1.4E-02 | *rv3479* | Possible transmembrane protein | -2.1 |
| 1.3E-03 | *rv0013* | Possible anthranilate synthase component II TrpG (glutamine amidotransferase) | -2.2 |
| 1.5E-02 | *rv0447c* | Probable cyclopropane-fatty-acyl-phospholipid synthase UfaA1 (cyclopropane fatty acid synthase) (CFA synthase) | -2.2 |
| 3.1E-03 | *rv1655* | Probable acetylornithine aminotransferase ArgD | -2.2 |
| 1.3E-03 | *rv3443c* | 50S ribosomal protein L13 RplM | -2.2 |
| 1.4E-03 | *rv0465c* | Probable transcriptional regulatory protein | -2.2 |
| 1.8E-02 | *rv0494* | Probable transcriptional regulatory protein (probably GntR-family) | -2.2 |
| 6.6E-03 | *rv0432* | Periplasmic superoxide dismutase [Cu-Zn] SodC | -2.2 |
| 1.0E-02 | *rv2757c* | Possible toxin VapC21 | -2.2 |
| 2.5E-03 | *rv1169c* | PE family protein. Possible lipase LipX. | -2.2 |
| 1.9E-02 | *rv3752c* | Possible cytidine/deoxycytidylate deaminase | -2.2 |
| 6.4E-02 | *rv2646* | Probable integrase | -2.2 |
| 1.2E-03 | *rv3924c* | 50S ribosomal protein L34 RpmH | -2.2 |
| 9.7E-03 | *rv1031* | Probable potassium-transporting ATPase C chain KdpC (potassium-translocating ATPase C chain) (ATP phosphohydrolase [potassium-transporting] C chain) (potassium binding and translocating subunit C) | -2.2 |
| 1.1E-02 | *rv1495* | Possible toxin MazF4 | -2.2 |
| 1.1E-02 | *rv2964* | Probable formyltetrahydrofolate deformylase PurU (formyl-FH(4) hydrolase) | -2.2 |
| 3.2E-02 | *rv3076* | Conserved hypothetical protein | -2.2 |
| 7.1E-03 | *rv2486* | Probable enoyl-CoA hydratase EchA14 (enoyl hydrase) (unsaturated acyl-CoA hydratase) (crotonase) | -2.2 |
| 9.7E-04 | *rv1657* | Probable arginine repressor ArgR (AHRC) | -2.2 |
| 4.7E-02 | *rv2602* | Possible toxin VapC41. Contains PIN domain. | -2.2 |
| 8.8E-03 | *rv1179c* | Unknown protein | -2.2 |
| 8.0E-02 | *rv1788* | PE family protein PE18 | -2.2 |
| 2.4E-02 | *rv0421c* | Conserved hypothetical protein | -2.2 |
| 3.1E-02 | *rv2529* | Hypothetical protein | -2.2 |
| 5.1E-02 | *rv0753c* | Probable methylmalonate-semialdehyde dehydrogenase MmsA (methylmalonic acid semialdehyde dehydrogenase) (MMSDH) | -2.2 |
| 2.4E-02 | *rv0959A* | Possible antitoxin VapB9 | -2.2 |
| 1.3E-02 | *rv2012* | Conserved hypothetical protein | -2.2 |
| 3.2E-03 | *rv2466c* | Conserved protein | -2.3 |
| 1.2E-02 | *rv1453* | Possible transcriptional activator protein | -2.3 |
| 6.3E-02 | *rv3135* | PPE family protein PPE50 | -2.3 |
| 2.0E-02 | *rv0587* | Conserved hypothetical integral membrane protein YrbE2A | -2.3 |
| 4.9E-02 | *rv3352c* | Possible oxidoreductase | -2.3 |
| 1.8E-02 | *rv3405c* | Possible transcriptional regulatory protein | -2.3 |
| 1.3E-02 | *rv1944c* | Conserved protein | -2.3 |
| 4.8E-03 | *rv1200* | Probable conserved integral membrane transport protein | -2.3 |
| 2.8E-02 | *rv3705A* | Conserved hypothetical proline rich protein | -2.3 |
| 2.7E-03 | *rv3102c* | Putative cell division ATP-binding protein FtsE (septation component-transport ATP-binding protein ABC transporter) | -2.3 |
| 2.2E-02 | *rv2378c* | Lysine-N-oxygenase MbtG (L-lysine 6-monooxygenase) (lysine N6-hydroxylase) | -2.3 |
| 3.3E-02 | *rv2331* | Hypothetical protein | -2.3 |
| 2.1E-02 | *rv1723* | Probable hydrolase | -2.3 |
| 6.9E-02 | *rv2803* | Conserved hypothetical protein | -2.3 |
| 3.5E-02 | *rv3425* | PPE family protein PPE57 | -2.3 |
| 1.1E-02 | *rv3090* | Unknown alanine and valine rich protein | -2.3 |
| 3.0E-03 | *rv1659* | Probable argininosuccinate lyase ArgH | -2.3 |
| 5.5E-03 | *rv0651* | 50S ribosomal protein L10 RplJ | -2.3 |
| 3.7E-02 | *rv3351c* | Conserved hypothetical protein | -2.4 |
| 7.8E-03 | *rv3796* | Conserved protein | -2.4 |
| 1.2E-02 | *rv1637c* | Conserved protein | -2.4 |
| 1.2E-03 | *rv1956* | Possible antitoxin HigA | -2.4 |
| 1.2E-02 | *rv1541c* | Possible lipoprotein LprI | -2.4 |
| 4.6E-03 | *rv0822c* | Conserved protein | -2.4 |
| 1.5E-02 | *rv1158c* | Conserved hypothetical ala-%2C pro-rich protein | -2.4 |
| 3.5E-02 | *rv1482c* | Conserved hypothetical protein | -2.4 |
| 2.3E-04 | *rv1186c* | Conserved protein | -2.4 |
| 1.4E-02 | *rv2767c* | Possible membrane protein | -2.4 |
| 1.3E-02 | *rv2019* | Conserved protein | -2.4 |
| 3.9E-02 | *rv2667* | Possible ATP-dependent protease ATP-binding subunit ClpC2 | -2.4 |
| 1.2E-02 | *rv3195* | Conserved hypothetical protein | -2.4 |
| 1.4E-02 | *rv2874* | Possible integral membrane C-type cytochrome biogenesis protein DipZ | -2.4 |
| 5.3E-02 | *rv0456B* | Possible antitoxin MazE1 | -2.4 |
| 1.1E-03 | *rv3909* | Conserved protein | -2.4 |
| 8.3E-04 | *rv1273c* | Probable drugs-transport transmembrane ATP-binding protein ABC transporter | -2.5 |
| 1.9E-04 | *rv2412* | 30S ribosomal protein S20 RpsT | -2.5 |
| 1.3E-02 | *rv2843* | Probable conserved transmembrane alanine rich protein | -2.5 |
| 4.1E-03 | *rv1171* | Conserved hypothetical protein | -2.5 |
| 1.5E-02 | *rv3706c* | Conserved hypothetical proline rich protein | -2.5 |
| 5.0E-03 | *rv2700* | Possible conserved secreted alanine rich protein | -2.5 |
| 3.4E-05 | *rv1879* | Conserved hypothetical protein | -2.5 |
| 3.0E-02 | *rv1349* | Iron-regulated transporter IrtB | -2.5 |
| 5.8E-02 | *rv2958c* | Possible glycosyl transferase | -2.5 |
| 2.8E-02 | *rv1246c* | Toxin RelE | -2.5 |
| 9.4E-02 | *rv2544* | Probable conserved lipoprotein LppB | -2.5 |
| 2.9E-02 | *rv1233c* | Conserved hypothetical membrane protein | -2.5 |
| 5.6E-04 | *rv1957* | Hypothetical protein | -2.5 |
| 7.6E-02 | *rv2307D* | Hypothetical protein | -2.5 |
| 2.5E-02 | *rv2643* | Probable arsenic-transport integral membrane protein ArsC | -2.5 |
| 5.2E-03 | *rv3506* | Fatty-acid-CoA synthetase FadD17 (fatty-acid-CoA synthase) (fatty-acid-CoA ligase) | -2.5 |
| 8.9E-02 | *rv3427c* | Possible transposase | -2.5 |
| 5.3E-03 | *rv0191* | Probable conserved integral membrane protein | -2.5 |
| 4.9E-03 | *rv0960* | Possible toxin VapC9 | -2.6 |
| 1.0E-02 | *rv0449c* | Conserved hypothetical protein | -2.6 |
| 1.4E-03 | *rv0385* | Probable monooxygenase | -2.6 |
| 4.6E-03 | *rv2050* | Conserved protein | -2.6 |
| 4.3E-04 | *rv1230c* | Possible membrane protein | -2.6 |
| 1.5E-02 | *rv1693* | Conserved hypothetical protein | -2.6 |
| 1.4E-02 | *rv1255c* | Probable transcriptional regulatory protein | -2.6 |
| 8.4E-04 | *rv1520* | Probable sugar transferase | -2.6 |
| 6.7E-03 | *rv3384c* | Possible toxin VapC46. Contains PIN domain. | -2.6 |
| 1.2E-02 | *rv0303* | Probable dehydrogenase/reductase | -2.6 |
| 9.2E-03 | *rv2290* | Probable conserved lipoprotein LppO | -2.6 |
| 2.3E-03 | *rv1244* | Probable lipoprotein LpqZ | -2.6 |
| 2.0E-02 | *rv2377c* | Putative conserved protein MbtH | -2.6 |
| 2.5E-02 | *rv1946c* | Possible lipoprotein | -2.7 |
| 1.5E-04 | *rv1860* | Alanine and proline rich secreted protein Apa (fibronectin attachment protein) (immunogenic protein MPT32) (antigen MPT-32) (45-kDa glycoprotein) (45/47 kDa antigen) | -2.7 |
| 6.2E-04 | *rv0765c* | Probable oxidoreductase | -2.7 |
| 1.9E-03 | *rv0923c* | Conserved hypothetical protein | -2.7 |
| 3.1E-03 | *rv2203* | Possible conserved membrane protein | -2.7 |
| 2.3E-02 | *rv1271c* | Conserved hypothetical secreted protein | -2.7 |
| 8.9E-03 | *rv1039c* | PPE family protein PPE15 | -2.7 |
| 4.2E-03 | *rv1579c* | Probable PhiRv1 phage protein | -2.7 |
| 8.6E-04 | *rv2582* | Probable peptidyl-prolyl cis-trans isomerase B PpiB (cyclophilin) (PPIase) (rotamase) (peptidylprolyl isomerase) | -2.7 |
| 1.0E-02 | *rv2988c* | Probable 3-isopropylmalate dehydratase (large subunit) LeuC (isopropylmalate isomerase) (alpha-IPM isomerase) (IPMI) | -2.8 |
| 8.2E-04 | *rv1184c* | Possible exported protein | -2.8 |
| 1.3E-03 | *rv3390* | Probable conserved lipoprotein LpqD | -2.8 |
| 7.8E-03 | *rv0302* | Probable transcriptional regulatory protein (probably TetR/AcrR-family) | -2.8 |
| 4.1E-02 | *rv1621c* | Probable 'component linked with the assembly of cytochrome' transport transmembrane ATP-binding protein ABC transporter CydD | -2.8 |
| 2.5E-03 | *rv3750c* | Possible excisionase | -2.8 |
| 6.9E-03 | *rv3789* | GTRA family protein | -2.8 |
| 9.5E-02 | *rv3861* | Hypothetical protein | -2.8 |
| 5.8E-03 | *rv0542c* | Possible O-succinylbenzoic acid--CoA ligase MenE (OSB-CoA synthetase) (O-succinylbenzoate-CoA synthase) | -2.8 |
| 3.5E-05 | *rv3094c* | Conserved hypothetical protein | -2.8 |
| 1.2E-03 | *rv2957* | Possible glycosyl transferase | -2.8 |
| 2.2E-03 | *rv2370c* | Conserved hypothetical protein | -2.8 |
| 7.5E-04 | *rv3145* | Probable NADH dehydrogenase I (chain A) NuoA (NADH-ubiquinone oxidoreductase chain A) | -2.8 |
| 2.8E-04 | *rv0310c* | Conserved protein | -2.8 |
| 4.2E-04 | *rv1256c* | Probable cytochrome P450 130 Cyp130 | -2.8 |
| 2.3E-02 | *rv0521* | Possible methyltransferase/methylase (fragment) | -2.8 |
| 1.3E-02 | *rv3833* | Transcriptional regulatory protein (probably AraC-family) | -2.9 |
| 6.1E-03 | *rv2641* | Cadmium inducible protein CadI | -2.9 |
| 2.4E-02 | *rv0815c* | Probable thiosulfate sulfurtransferase CysA2 (rhodanese-like protein) (thiosulfate cyanide transsulfurase) (thiosulfate thiotransferase) | -2.9 |
| 3.9E-02 | *rv0767c* | Conserved hypothetical protein | -2.9 |
| 1.1E-03 | *rv0250c* | Conserved protein | -3.0 |
| 6.4E-02 | *rv1134* | Hypothetical protein | -3.0 |
| 2.2E-03 | *rv0970* | Probable conserved integral membrane protein | -3.0 |
| 4.1E-03 | *rv1638A* | Conserved hypothetical protein | -3.0 |
| 2.3E-02 | *rv1999c* | Probable conserved integral membrane protein | -3.1 |
| 8.6E-04 | *rv0725c* | Conserved hypothetical protein | -3.1 |
| 2.0E-03 | *rv3717* | Conserved hypothetical protein | -3.1 |
| 3.5E-04 | *rv1516c* | Probable sugar transferase | -3.2 |
| 7.8E-04 | *rv2473* | Possible alanine and proline rich membrane protein | -3.2 |
| 4.9E-03 | *rv3772* | Probable histidinol-phosphate aminotransferase HisC2 (imidazole acetol-phosphate transaminase) (imidazolylacetolphosphate aminotransferase) | -3.2 |
| 1.4E-03 | *rv1471* | Probable thioredoxin TrxB1 | -3.2 |
| 6.2E-05 | *rv1895* | Possible dehydrogenase | -3.2 |
| 2.2E-03 | *rv0076c* | Probable membrane protein | -3.2 |
| 1.1E-02 | *rv2384* | Bifunctional enzyme MbtA: salicyl-AMP ligase (SAL-AMP ligase) + salicyl-S-ArCP synthetase | -3.2 |
| 1.8E-03 | *rv0322* | Probable UDP-glucose 6-dehydrogenase UdgA (UDP-GLC dehydrogenase) (UDP-GLCDH) (UDPGDH) | -3.2 |
| 1.2E-02 | *rv2642* | Possible transcriptional regulatory protein (probably ArsR-family) | -3.3 |
| 5.1E-04 | *rv1262c* | Hypothetical hit-like protein | -3.3 |
| 7.3E-04 | *rv2972c* | Possible conserved membrane or exported protein | -3.4 |
| 4.7E-03 | *rv1582c* | Probable PhiRv1 phage protein | -3.4 |
| 6.9E-04 | *rv2754c* | Probable thymidylate synthase ThyX (ts) (TSase) | -3.4 |
| 4.1E-03 | *rv0614* | Conserved hypothetical protein | -3.4 |
| 1.2E-02 | *rv2647* | Hypothetical protein | -3.5 |
| 2.3E-02 | *rv1765A* | Putative transposase (fragment) | -3.5 |
| 1.2E-03 | *rv2009* | Antitoxin VapB15 | -3.5 |
| 1.5E-02 | *rv1586c* | Probable PhiRv1 integrase | -3.5 |
| 1.2E-03 | *rv0717* | 30S ribosomal protein S14 RpsN1 | -3.6 |
| 2.0E-02 | *rv0329c* | Conserved hypothetical protein | -3.6 |
| 2.3E-03 | *rv0789c* | Hypothetical protein | -3.6 |
| 9.5E-03 | *rv1081c* | Probable conserved membrane protein | -3.7 |
| 2.0E-03 | *rv2873* | Cell surface lipoprotein Mpt83 (lipoprotein P23) | -3.7 |
| 3.5E-02 | *rv2666* | Probable transposase for insertion sequence element IS1081 (fragment) | -3.7 |
| 1.4E-03 | *rv1580c* | Probable PhiRv1 phage protein | -3.7 |
| 1.3E-03 | *rv0001* | Chromosomal replication initiator protein DnaA | -3.8 |
| 1.0E-03 | *rv2016* | Hypothetical protein | -3.8 |
| 1.2E-03 | *rv2989* | Probable transcriptional regulatory protein | -3.8 |
| 1.9E-04 | *rv2911* | Probable penicillin-binding protein DacB2 (D-alanyl-D-alanine carboxypeptidase) (DD-peptidase) (DD-carboxypeptidase) (PBP) (DD-transpeptidase) (serine-type D-ala-D-ala carboxypeptidase) (D-amino acid hydrolase) | -3.8 |
| 3.8E-02 | *rv3839* | Conserved hypothetical protein | -3.8 |
| 3.1E-05 | *rv3911* | Possible alternative RNA polymerase sigma factor SigM | -3.9 |
| 5.4E-05 | *rv0165c* | Probable transcriptional regulatory protein Mce1R (probably GntR-family) | -3.9 |
| 8.7E-02 | *rv0327c* | Possible cytochrome P450 135A1 Cyp135A1 | -3.9 |
| 1.2E-02 | *rv0330c* | Hypothetical protein | -4.0 |
| 9.7E-02 | *rv0793* | Possible monooxygenase | -4.1 |
| 7.2E-02 | *rv1954c* | Hypothetical protein | -4.1 |
| 2.3E-02 | *rv1519* | Conserved hypothetical protein | -4.1 |
| 1.6E-04 | *rv0104* | Conserved hypothetical protein | -4.2 |
| 4.5E-05 | *rv1632c* | Hypothetical protein | -4.2 |
| 2.2E-02 | *rv0150c* | Conserved hypothetical protein | -4.3 |
| 1.1E-02 | *rv0790c* | Hypothetical protein | -4.3 |
| 2.9E-04 | *rv2912c* | Probable transcriptional regulatory protein (probably TetR-family) | -4.4 |
| 2.0E-02 | *rv1958c* | Hypothetical protein | -4.4 |
| 4.1E-05 | *rv0331* | Possible dehydrogenase/reductase | -4.4 |
| 3.1E-03 | *rv1584c* | Possible PhiRv1 phage protein | -4.4 |
| 4.7E-03 | *rv1620c* | Probable 'component linked with the assembly of cytochrome' transport transmembrane ATP-binding protein ABC transporter CydC | -4.5 |
| 1.2E-02 | *rv2662* | Hypothetical protein | -4.6 |
| 4.4E-03 | *rv0791c* | Conserved protein | -4.7 |
| 1.4E-03 | *rv2386c* | Isochorismate synthase MbtI | -4.8 |
| 7.2E-03 | *rv3269* | Conserved protein | -5.0 |
| 8.5E-03 | *rv0792c* | Probable transcriptional regulatory protein (probably GntR-family) | -5.0 |
| 6.0E-05 | *rv3463* | Conserved protein | -5.2 |
| 1.0E-03 | *rv3354* | Conserved hypothetical protein | -5.3 |
| 3.1E-02 | *rv2270* | Probable lipoprotein LppN | -5.3 |
| 1.9E-02 | *rv3108* | Hypothetical protein | -5.4 |
| 1.3E-04 | *rv2034* | ArsR repressor protein | -5.5 |
| 1.2E-03 | *rv3167c* | Probable transcriptional regulatory protein (probably TetR-family) | -5.7 |
| 2.1E-02 | *rv0661c* | Possible toxin VapC7 | -5.7 |
| 8.8E-04 | *rv3012c* | Probable glutamyl-tRNA(GLN) amidotransferase (subunit C) GatC (Glu-ADT subunit C) | -6.0 |
| 3.1E-03 | *rv1583c* | Probable PhiRv1 phage protein | -6.0 |
| 2.8E-02 | *rv1734c* | Conserved hypothetical protein | -6.0 |
| 3.6E-03 | *rv0251c* | Heat shock protein Hsp (heat-stress-induced ribosome-binding protein A) | -6.1 |
| 1.2E-03 | *rv3402c* | Conserved hypothetical protein | -6.2 |
| 1.5E-05 | *rv2913c* | Possible D-amino acid aminohydrolase (D-amino acid hydrolase) | -6.5 |
| 1.0E-04 | *rv3312A* | Secreted protein antigen | -6.6 |
| 6.8E-02 | *rv0745* | Conserved hypothetical protein | -6.6 |
| 1.9E-02 | *rv0662c* | Possible antitoxin VapB7 | -6.6 |
| 9.0E-06 | *rv0846c* | Probable oxidase | -6.8 |
| 2.7E-04 | *rv2963* | Probable integral membrane protein | -7.6 |
| 2.1E-03 | *rv3270* | Probable metal cation-transporting P-type ATPase C CtpC | -7.7 |
| 2.2E-02 | *rv2653c* | Possible PhiRv2 prophage protein | -8.0 |
| 2.0E-03 | *rv3054c* | Conserved hypothetical protein | -8.4 |
| 1.6E-02 | *rv1089A* | Probable cellulase CelA2a (endo-1%2C4-beta-glucanase) (endoglucanase) (carboxymethyl cellulase) | -8.4 |
| 8.5E-05 | *rv0849* | Probable conserved integral membrane transport protein | -10.2 |
| 5.4E-03 | *rv0850* | Putative transposase (fragment) | -12.1 |
| 8.2E-05 | *rv0848* | Possible cysteine synthase a CysK2 (O-acetylserine sulfhydrylase) (O-acetylserine (thiol)-lyase) (CSASE) | -14.2 |
| 8.0E-03 | *rv2123* | PPE family protein PPE37 | -15.5 |
| 3.4E-02 | *rv2660c* | Hypothetical protein | -16.7 |
| 2.7E-02 | *rv1119c* | Hypothetical protein | -17.1 |
| 2.3E-03 | *rv0448c* | Conserved hypothetical protein | -17.9 |
| 5.2E-04 | *rv0186A* | Metallothionein%2C MymT | -18.5 |
| 1.1E-02 | *rv1755c* | Probable phospholipase C 4 (fragment) PlcD | -22.7 |
| 1.4E-04 | *rv0847* | Probable lipoprotein LpqS | -25.5 |
| 1.3E-02 | *rv1037c* | Putative ESAT-6 like protein EsxI (ESAT-6 like protein 1) | -26.0 |
| 1.5E-02 | *rv1041c* | Probable is like-2 transposase | -34.4 |

## **Supplementary Table.5**

| **Supplementary Table.5 Genes that are differentially regulated in the ∆*rv0191* mutant relative to the wild-type (in Sauton’s media)** | | | |
| --- | --- | --- | --- |
| **p_value** | **gene** | **gene product** | **FC** |
| 2.8E-02 | *rv2058c* | 50S ribosomal protein L28 RpmB2 | 7.4 |
| 2.8E-02 | *rv2057c* | 50S ribosomal protein L33 RpmG1 | 6.5 |
| 4.6E-02 | *rv2056c* | 30S ribosomal protein S14 RpsN2 | 5.6 |
| 2.9E-02 | *rv0280* | PPE family protein PPE3 | 5.5 |
| 5.9E-03 | *rv1057* | Conserved hypothetical protein | 5.3 |
| 5.6E-02 | *rv1405c* | Putative methyltransferase | 5.1 |
| 4.2E-02 | *rv2628* | Hypothetical protein | 4.8 |
| 1.7E-02 | *rv0096* | PPE family protein PPE1 | 4.6 |
| 8.5E-03 | *rv1854c* | Probable NADH dehydrogenase Ndh | 4.5 |
| 2.7E-02 | *rv1996* | Universal stress protein family protein | 4.4 |
| 4.6E-02 | *rv2780* | Secreted L-alanine dehydrogenase Ald (40 kDa antigen) (TB43) | 4.4 |
| 3.1E-02 | *rv2055c* | 30S ribosomal protein S18 RpsR2 | 4.2 |
| 4.3E-02 | *rv0106* | Conserved hypothetical protein | 4.2 |
| 7.8E-02 | *rv3746c* | Probable PE family protein PE34 (PE family-related protein) | 4.2 |
| 5.4E-02 | *rv3130c* | Triacylglycerol synthase (diacylglycerol acyltransferase) Tgs1 | 4.1 |
| 1.4E-02 | *rv3503c* | Probable ferredoxin FdxD | 3.9 |
| 3.7E-02 | *rv1403c* | Putative methyltransferase | 3.9 |
| 2.3E-02 | *rv3139* | Probable acyl-CoA dehydrogenase FadE24 | 3.9 |
| 2.5E-02 | *rv3574* | Transcriptional regulatory protein KstR (probably TetR-family) | 3.8 |
| 7.1E-02 | *rv2627c* | Conserved protein | 3.7 |
| 8.1E-02 | *rv0575c* | Possible oxidoreductase | 3.6 |
| 4.0E-02 | *rv0285* | PE family protein PE5 | 3.5 |
| 5.0E-02 | *rv0282* | ESX conserved component EccA3. ESX-3 type VII secretion system protein. | 3.5 |
| 5.0E-02 | *rv3188* | Conserved hypothetical protein | 3.5 |
| 5.3E-02 | *rv0288* | Low molecular weight protein antigen 7 EsxH (10 kDa antigen) (CFP-7) (protein TB10.4) | 3.3 |
| 8.3E-02 | *rv1460* | Probable transcriptional regulatory protein | 3.2 |
| 8.8E-02 | *rv2623* | Universal stress protein family protein TB31.7 | 3.2 |
| 4.5E-02 | *rv0274* | Conserved protein | 3.2 |
| 5.3E-02 | *rv2990c* | Hypothetical protein | 3.1 |
| 4.0E-02 | *rv0286* | PPE family protein PPE4 | 3.1 |
| 5.6E-02 | *rv1578c* | Probable PhiRv1 phage protein | 3.1 |
| 2.8E-02 | *rv2729c* | Probable conserved integral membrane alanine valine and leucine rich protein | 3.1 |
| 4.6E-02 | *rv0283* | ESX conserved component EccB3. ESX-3 type VII secretion system protein. Possible membrane protein. | 3.1 |
| 3.2E-02 | *rv3140* | Probable acyl-CoA dehydrogenase FadE23 | 3.0 |
| 5.0E-02 | *rv0289* | ESX-3 secretion-associated protein EspG3 | 3.0 |
| 2.7E-02 | *rv0677c* | Possible conserved membrane protein MmpS5 | 3.0 |
| 6.6E-02 | *rv0287* | ESAT-6 like protein EsxG (conserved protein TB9.8) | 3.0 |
| 2.2E-02 | *rv0067c* | Possible transcriptional regulatory protein (possibly TetR-family) | 3.0 |
| 6.6E-02 | *rv0678* | Conserved protein | 2.9 |
| 5.9E-03 | *rv0099* | Possible fatty-acid-CoA ligase FadD10 (fatty-acid-CoA synthetase) (fatty-acid-CoA synthase) | 2.8 |
| 5.1E-02 | *rv0290* | ESX conserved component EccD3. ESX-3 type VII secretion system protein. Probable transmembrane protein. | 2.8 |
| 1.5E-02 | *rv2927c* | Conserved hypothetical protein | 2.8 |
| 6.4E-02 | *rv0281* | Possible S-adenosylmethionine-dependent methyltransferase | 2.8 |
| 3.1E-02 | *rv0996* | Probable conserved transmembrane protein | 2.8 |
| 1.0E-02 | *rv2549c* | Possible toxin VapC20 | 2.8 |
| 9.0E-03 | *rv2550c* | Possible antitoxin VapB20 | 2.7 |
| 3.3E-02 | *rv2557* | Conserved protein | 2.7 |
| 6.8E-02 | *rv0284* | ESX conserved component EccC3. ESX-3 type VII secretion system protein. Possible membrane protein. | 2.7 |
| 1.2E-02 | *rv1159A* | Unknown protein | 2.7 |
| 1.1E-02 | *rv0098* | Probable fatty acyl CoA thioesterase type III FcoT | 2.6 |
| 2.3E-02 | *rv1419* | Unknown protein | 2.6 |
| 5.6E-02 | *rv1577c* | Probable PhiRv1 phage protein | 2.6 |
| 3.7E-02 | *rv1670* | Conserved hypothetical protein | 2.5 |
| 3.1E-02 | *rv0097* | Possible oxidoreductase | 2.5 |
| 3.0E-02 | *rv3016* | Probable lipoprotein LpqA | 2.5 |
| 8.1E-02 | *rv2063* | Antitoxin MazE7 | 2.4 |
| 1.6E-02 | *rv0116c* | Probable L%2CD-transpeptidase LdtA | 2.4 |
| 6.6E-02 | *rv0291* | Probable membrane-anchored mycosin MycP3 (serine protease) (subtilisin-like protease) (subtilase-like) (mycosin-3) | 2.4 |
| 7.4E-02 | *rv1076* | Possible lipase LipU | 2.4 |
| 6.0E-02 | *rv1888c* | Possible transmembrane protein | 2.4 |
| 7.3E-02 | *rv1461* | Conserved protein | 2.4 |
| 9.8E-03 | *rv3122* | Hypothetical protein | 2.4 |
| 1.0E-01 | *rv1593c* | Conserved protein | 2.4 |
| 4.7E-02 | *rv0810c* | Conserved hypothetical protein | 2.3 |
| 7.6E-02 | *rv0188* | Probable conserved transmembrane protein | 2.3 |
| 9.3E-02 | *rv2830c* | Possible antitoxin VapB22 | 2.3 |
| 4.2E-03 | *rv0101* | Probable peptide synthetase Nrp (peptide synthase) | 2.3 |
| 9.4E-02 | *rv1088* | PE family protein PE9 | 2.3 |
| 6.2E-02 | *rv3841* | Bacterioferritin BfrB | 2.3 |
| 7.2E-02 | *rv1894c* | Conserved hypothetical protein | 2.3 |
| 6.9E-02 | *rv1404* | Probable transcriptional regulatory protein | 2.2 |
| 6.8E-02 | *rv1063c* | Conserved hypothetical protein | 2.2 |
| 7.6E-02 | *rv2059* | Conserved hypothetical protein | 2.2 |
| 4.6E-02 | *rv1152* | Probable transcriptional regulatory protein | 2.2 |
| 3.6E-02 | *rv0898c* | Conserved hypothetical protein | 2.1 |
| 1.4E-03 | *rv0100* | Conserved hypothetical protein | 2.1 |
| 4.9E-03 | *rv1972* | Probable conserved Mce associated membrane protein | 2.1 |
| 5.6E-02 | *rv3760* | Possible conserved membrane protein | 2.1 |
| 6.5E-02 | *rv0757* | Possible two component system response transcriptional positive regulator PhoP | 2.1 |
| 8.7E-02 | *rv2650c* | Possible PhiRv2 prophage protein | 2.1 |
| 4.6E-02 | *rv1332* | Probable transcriptional regulatory protein | 2.1 |
| 8.0E-02 | *rv3288c* | Putative protein UsfY | 2.1 |
| 3.9E-02 | *rv1539* | Probable lipoprotein signal peptidase LspA | 2.1 |
| 4.9E-02 | *rv2327* | Conserved protein | 2.1 |
| 4.6E-02 | *rv1779c* | Possible integral membrane protein | 2.1 |
| 3.1E-02 | *rv3010c* | Probable 6-phosphofructokinase PfkA (phosphohexokinase) (phosphofructokinase) | 2.1 |
| 4.0E-02 | *rv1065* | Conserved hypothetical protein | 2.1 |
| 7.6E-02 | *rv1594* | Probable quinolinate synthetase NadA | 2.0 |
| 8.6E-02 | *rv1599* | Probable histidinol dehydrogenase HisD (HDH) | 2.0 |
| 7.0E-02 | *rv2558* | Conserved protein | 2.0 |
| 3.2E-02 | *rv0397A* | Conserved protein | 2.0 |
| 9.9E-02 | *rv2651c* | Possible PhiRv2 prophage protease | 2.0 |
| 8.2E-02 | *rv2497c* | Probable branched-chain keto acid dehydrogenase E1 component%2C alpha subunit BkdA | 2.0 |
| 4.6E-02 | *rv0311* | Unknown protein | 2.0 |
| 6.6E-02 | *rv3246c* | Two component sensory transduction transcriptional regulatory protein MtrA | 2.0 |
| 3.9E-02 | *rv2473* | Possible alanine and proline rich membrane protein | -2.0 |
| 4.1E-02 | *rv0960* | Possible toxin VapC9 | -2.0 |
| 3.5E-02 | *rv2203* | Possible conserved membrane protein | -2.0 |
| 4.6E-03 | *rv1096* | Possible glycosyl hydrolase | -2.0 |
| 6.0E-03 | *rv1860* | Alanine and proline rich secreted protein Apa (fibronectin attachment protein) (immunogenic protein MPT32) (antigen MPT-32) (45-kDa glycoprotein) (45/47 kDa antigen) | -2.0 |
| 8.3E-03 | *rv1230c* | Possible membrane protein | -2.0 |
| 1.2E-02 | *rv0765c* | Probable oxidoreductase | -2.0 |
| 7.8E-02 | *rv2643* | Probable arsenic-transport integral membrane protein ArsC | -2.0 |
| 5.9E-02 | *rv1255c* | Probable transcriptional regulatory protein | -2.0 |
| 8.1E-02 | *rv2377c* | Putative conserved protein MbtH | -2.0 |
| 8.3E-02 | *rv0614* | Conserved hypothetical protein | -2.0 |
| 1.9E-02 | *rv3145* | Probable NADH dehydrogenase I (chain A) NuoA (NADH-ubiquinone oxidoreductase chain A) | -2.0 |
| 2.2E-02 | *rv2012* | Conserved hypothetical protein | -2.1 |
| 5.0E-02 | *rv1203c* | Hypothetical protein | -2.1 |
| 2.0E-02 | *rv1453* | Possible transcriptional activator protein | -2.1 |
| 3.9E-02 | *rv0322* | Probable UDP-glucose 6-dehydrogenase UdgA (UDP-GLC dehydrogenase) (UDP-GLCDH) (UDPGDH) | -2.1 |
| 1.9E-03 | *rv2412* | 30S ribosomal protein S20 RpsT | -2.1 |
| 1.3E-02 | *rv2582* | Probable peptidyl-prolyl cis-trans isomerase B PpiB (cyclophilin) (PPIase) (rotamase) (peptidylprolyl isomerase) | -2.1 |
| 1.6E-02 | *rv3911* | Possible alternative RNA polymerase sigma factor SigM | -2.1 |
| 2.7E-02 | *rv1518* | Conserved hypothetical protein | -2.1 |
| 1.7E-02 | *rv1244* | Probable lipoprotein LpqZ | -2.1 |
| 1.8E-03 | *rv3924c* | 50S ribosomal protein L34 RpmH | -2.1 |
| 3.7E-03 | *rv1956* | Possible antitoxin HigA | -2.1 |
| 3.4E-03 | *rv1957* | Hypothetical protein | -2.1 |
| 3.6E-02 | *rv2874* | Possible integral membrane C-type cytochrome biogenesis protein DipZ | -2.1 |
| 5.7E-02 | *rv0324* | Possible transcriptional regulatory protein (possibly ArsR-family) | -2.2 |
| 8.1E-02 | *rv3135* | PPE family protein PPE50 | -2.2 |
| 2.5E-02 | *rv0653c* | Possible transcriptional regulatory protein (probably TetR-family) | -2.2 |
| 1.3E-02 | *rv2723* | Probable conserved integral membrane protein | -2.2 |
| 1.1E-02 | *rv0822c* | Conserved protein | -2.2 |
| 3.0E-02 | *rv2972c* | Possible conserved membrane or exported protein | -2.2 |
| 2.5E-02 | *rv3143* | Probable response regulator | -2.2 |
| 8.9E-03 | *rv1495* | Possible toxin MazF4 | -2.2 |
| 3.4E-02 | *rv0959A* | Possible antitoxin VapB9 | -2.2 |
| 5.3E-02 | *rv1585c* | Possible phage PhiRv1 protein | -2.2 |
| 3.0E-02 | *rv0449c* | Conserved hypothetical protein | -2.2 |
| 4.6E-02 | *rv1921c* | Probable conserved lipoprotein LppF | -2.2 |
| 1.7E-02 | *rv0749A* | Hypothetical protein (fragment) | -2.2 |
| 6.5E-02 | *rv1271c* | Conserved hypothetical secreted protein | -2.2 |
| 6.0E-03 | *rv1520* | Probable sugar transferase | -2.2 |
| 1.4E-03 | *rv2017* | Transcriptional regulatory protein | -2.2 |
| 3.3E-02 | *rv3327* | Probable transposase fusion protein | -2.2 |
| 2.5E-02 | *rv1471* | Probable thioredoxin TrxB1 | -2.2 |
| 3.0E-02 | *rv0542c* | Possible O-succinylbenzoic acid--CoA ligase MenE (OSB-CoA synthetase) (O-succinylbenzoate-CoA synthase) | -2.2 |
| 2.1E-02 | *rv1579c* | Probable PhiRv1 phage protein | -2.2 |
| 2.0E-02 | *rv3830c* | Transcriptional regulatory protein (probably TetR-family) | -2.2 |
| 4.4E-02 | *rv0789c* | Hypothetical protein | -2.3 |
| 8.7E-02 | *rv2647* | Hypothetical protein | -2.3 |
| 1.4E-02 | *rv2370c* | Conserved hypothetical protein | -2.3 |
| 5.9E-03 | *rv1549* | Possible fatty-acid-CoA ligase FadD11.1 (fatty-acid-CoA synthetase) (fatty-acid-CoA synthase) | -2.3 |
| 8.0E-02 | *rv1788* | PE family protein PE18 | -2.3 |
| 2.9E-02 | *rv2875* | Major secreted immunogenic protein Mpt70 | -2.3 |
| 7.5E-03 | *rv0447c* | Probable cyclopropane-fatty-acyl-phospholipid synthase UfaA1 (cyclopropane fatty acid synthase) (CFA synthase) | -2.3 |
| 1.9E-02 | *rv0402c* | Probable conserved transmembrane transport protein MmpL1 | -2.4 |
| 4.0E-02 | *rv0521* | Possible methyltransferase/methylase (fragment) | -2.4 |
| 6.7E-03 | *rv1516c* | Probable sugar transferase | -2.4 |
| 2.8E-02 | *rv3463* | Conserved protein | -2.4 |
| 6.2E-02 | *rv0815c* | Probable thiosulfate sulfurtransferase CysA2 (rhodanese-like protein) (thiosulfate cyanide transsulfurase) (thiosulfate thiotransferase) | -2.4 |
| 4.0E-04 | *rv3094c* | Conserved hypothetical protein | -2.4 |
| 1.4E-02 | *rv2754c* | Probable thymidylate synthase ThyX (ts) (TSase) | -2.4 |
| 7.1E-03 | *rv0250c* | Conserved protein | -2.4 |
| 1.4E-05 | *rv1176c* | Conserved hypothetical protein | -2.5 |
| 4.8E-04 | *rv1169c* | PE family protein. Possible lipase LipX. | -2.5 |
| 2.4E-02 | *rv2331* | Hypothetical protein | -2.5 |
| 6.0E-03 | *rv2917* | Conserved hypothetical alanine and arginine rich protein | -2.5 |
| 1.7E-02 | *rv1347c* | Lysine N-acetyltransferase MbtK | -2.5 |
| 2.8E-02 | *rv3833* | Transcriptional regulatory protein (probably AraC-family) | -2.5 |
| 1.3E-02 | *rv3789* | GTRA family protein | -2.5 |
| 8.5E-03 | *rv1632c* | Hypothetical protein | -2.5 |
| 9.0E-02 | *rv2489c* | Hypothetical alanine rich protein | -2.5 |
| 6.9E-02 | *rv1586c* | Probable PhiRv1 integrase | -2.5 |
| 5.4E-02 | *rv1999c* | Probable conserved integral membrane protein | -2.6 |
| 6.2E-02 | *rv3751* | Probable integrase (fragment) | -2.6 |
| 2.2E-03 | *rv2957* | Possible glycosyl transferase | -2.6 |
| 1.8E-03 | *rv2964* | Probable formyltetrahydrofolate deformylase PurU (formyl-FH(4) hydrolase) | -2.7 |
| 1.1E-02 | *rv0717* | 30S ribosomal protein S14 RpsN1 | -2.7 |
| 5.7E-04 | *rv1895* | Possible dehydrogenase | -2.7 |
| 4.5E-02 | *rv1621c* | Probable 'component linked with the assembly of cytochrome' transport transmembrane ATP-binding protein ABC transporter CydD | -2.7 |
| 7.9E-02 | *rv2662* | Hypothetical protein | -2.7 |
| 9.0E-03 | *rv0303* | Probable dehydrogenase/reductase | -2.7 |
| 4.7E-03 | *rv0970* | Probable conserved integral membrane protein | -2.7 |
| 1.1E-02 | *rv2016* | Hypothetical protein | -2.8 |
| 3.8E-03 | *rv2911* | Probable penicillin-binding protein DacB2 (D-alanyl-D-alanine carboxypeptidase) (DD-peptidase) (DD-carboxypeptidase) (PBP) (DD-transpeptidase) (serine-type D-ala-D-ala carboxypeptidase) (D-amino acid hydrolase) | -2.8 |
| 9.0E-03 | *rv2988c* | Probable 3-isopropylmalate dehydratase (large subunit) LeuC (isopropylmalate isomerase) (alpha-IPM isomerase) (IPMI) | -2.8 |
| 6.9E-03 | *rv2641* | Cadmium inducible protein CadI | -2.8 |
| 9.1E-02 | *rv2543* | Probable conserved lipoprotein LppA | -2.8 |
| 1.8E-03 | *rv0165c* | Probable transcriptional regulatory protein Mce1R (probably GntR-family) | -2.8 |
| 2.3E-02 | *rv2384* | Bifunctional enzyme MbtA: salicyl-AMP ligase (SAL-AMP ligase) + salicyl-S-ArCP synthetase | -2.9 |
| 1.1E-02 | *rv2034* | ArsR repressor protein | -2.9 |
| 5.0E-03 | *rv2009* | Antitoxin VapB15 | -2.9 |
| 3.1E-02 | *rv1081c* | Probable conserved membrane protein | -2.9 |
| 7.1E-02 | *rv0967* | Copper-sensitive operon repressor CsoR | -2.9 |
| 2.1E-02 | *rv2642* | Possible transcriptional regulatory protein (probably ArsR-family) | -2.9 |
| 5.2E-04 | *rv0366c* | Conserved hypothetical protein | -3.0 |
| 3.5E-02 | *rv1888A* | Conserved hypothetical protein | -3.0 |
| 7.7E-03 | *rv2873* | Cell surface lipoprotein Mpt83 (lipoprotein P23) | -3.0 |
| 2.1E-02 | *rv1581c* | Probable PhiRv1 phage protein | -3.0 |
| 1.7E-03 | *rv2019* | Conserved protein | -3.0 |
| 6.4E-03 | *rv2989* | Probable transcriptional regulatory protein | -3.1 |
| 7.5E-03 | *rv1582c* | Probable PhiRv1 phage protein | -3.1 |
| 2.0E-02 | *rv3012c* | Probable glutamyl-tRNA(GLN) amidotransferase (subunit C) GatC (Glu-ADT subunit C) | -3.1 |
| 4.7E-03 | *rv3425* | PPE family protein PPE57 | -3.2 |
| 1.4E-03 | *rv2253* | Possible secreted unknown protein | -3.2 |
| 6.1E-03 | *rv1482c* | Conserved hypothetical protein | -3.2 |
| 1.9E-02 | *rv1584c* | Possible PhiRv1 phage protein | -3.2 |
| 2.7E-03 | *rv0302* | Probable transcriptional regulatory protein (probably TetR/AcrR-family) | -3.2 |
| 6.0E-02 | *rv3395A* | Probable membrane protein | -3.2 |
| 2.6E-02 | *rv0329c* | Conserved hypothetical protein | -3.2 |
| 1.0E-02 | *rv3312A* | Secreted protein antigen | -3.3 |
| 1.0E-03 | *rv0104* | Conserved hypothetical protein | -3.4 |
| 3.4E-03 | *rv0001* | Chromosomal replication initiator protein DnaA | -3.4 |
| 6.1E-02 | *rv3839* | Conserved hypothetical protein | -3.4 |
| 9.4E-02 | *rv1089A* | Probable cellulase CelA2a (endo-1%2C4-beta-glucanase) (endoglucanase) (carboxymethyl cellulase) | -3.5 |
| 4.2E-04 | *rv0331* | Possible dehydrogenase/reductase | -3.5 |
| 2.6E-02 | *rv2666* | Probable transposase for insertion sequence element IS1081 (fragment) | -3.7 |
| 5.7E-02 | *rv2277c* | Possible glycerolphosphodiesterase | -3.8 |
| 1.1E-02 | *rv0791c* | Conserved protein | -3.9 |
| 4.6E-02 | *rv2491* | Conserved hypothetical protein | -4.0 |
| 9.1E-03 | *rv1620c* | Probable 'component linked with the assembly of cytochrome' transport transmembrane ATP-binding protein ABC transporter CydC | -4.0 |
| 1.8E-02 | *rv1583c* | Probable PhiRv1 phage protein | -4.0 |
| 9.8E-03 | *rv3167c* | Probable transcriptional regulatory protein (probably TetR-family) | -4.2 |
| 1.2E-02 | *rv0790c* | Hypothetical protein | -4.2 |
| 3.7E-03 | *rv3354* | Conserved hypothetical protein | -4.2 |
| 8.4E-02 | *rv0793* | Possible monooxygenase | -4.5 |
| 1.9E-02 | *rv1519* | Conserved hypothetical protein | -4.5 |
| 1.2E-02 | *rv1954c* | Hypothetical protein | -4.8 |
| 1.0E-04 | *rv2912c* | Probable transcriptional regulatory protein (probably TetR-family) | -4.9 |
| 7.0E-02 | *rv0378* | Conserved hypothetical glycine rich protein | -4.9 |
| 7.6E-03 | *rv0251c* | Heat shock protein Hsp (heat-stress-induced ribosome-binding protein A) | -5.2 |
| 6.0E-03 | *rv0792c* | Probable transcriptional regulatory protein (probably GntR-family) | -5.4 |
| 2.1E-03 | *rv0849* | Probable conserved integral membrane transport protein | -5.8 |
| 3.5E-04 | *rv2386c* | Isochorismate synthase MbtI | -5.9 |
| 6.4E-03 | *rv2546* | Possible toxin VapC18 | -6.2 |
| 2.5E-02 | *rv0448c* | Conserved hypothetical protein | -6.4 |
| 1.4E-05 | *rv2913c* | Possible D-amino acid aminohydrolase (D-amino acid hydrolase) | -6.6 |
| 5.3E-06 | *rv0846c* | Probable oxidase | -7.0 |
| 8.5E-04 | *rv3269* | Conserved protein | -7.7 |
| 2.0E-03 | *rv3270* | Probable metal cation-transporting P-type ATPase C CtpC | -7.8 |
| 2.1E-04 | *rv2963* | Probable integral membrane protein | -7.9 |
| 2.0E-03 | *rv0150c* | Conserved hypothetical protein | -8.0 |
| 1.9E-02 | *rv0850* | Putative transposase (fragment) | -8.2 |
| 1.7E-04 | *rv3402c* | Conserved hypothetical protein | -8.8 |
| 8.6E-04 | *rv3054c* | Conserved hypothetical protein | -9.7 |
| 9.1E-05 | *rv0848* | Possible cysteine synthase a CysK2 (O-acetylserine sulfhydrylase) (O-acetylserine (thiol)-lyase) (CSASE) | -13.9 |
| 9.6E-04 | *rv0186A* | Metallothionein%2C MymT | -15.5 |
| 7.7E-04 | *rv0847* | Probable lipoprotein LpqS | -15.7 |
| 6.7E-03 | *rv1755c* | Probable phospholipase C 4 (fragment) PlcD | -22.9 |
| 4.3E-03 | *rv0962c* | Possible lipoprotein LprP | -36.9 |

## **Supplementary Figure.1**


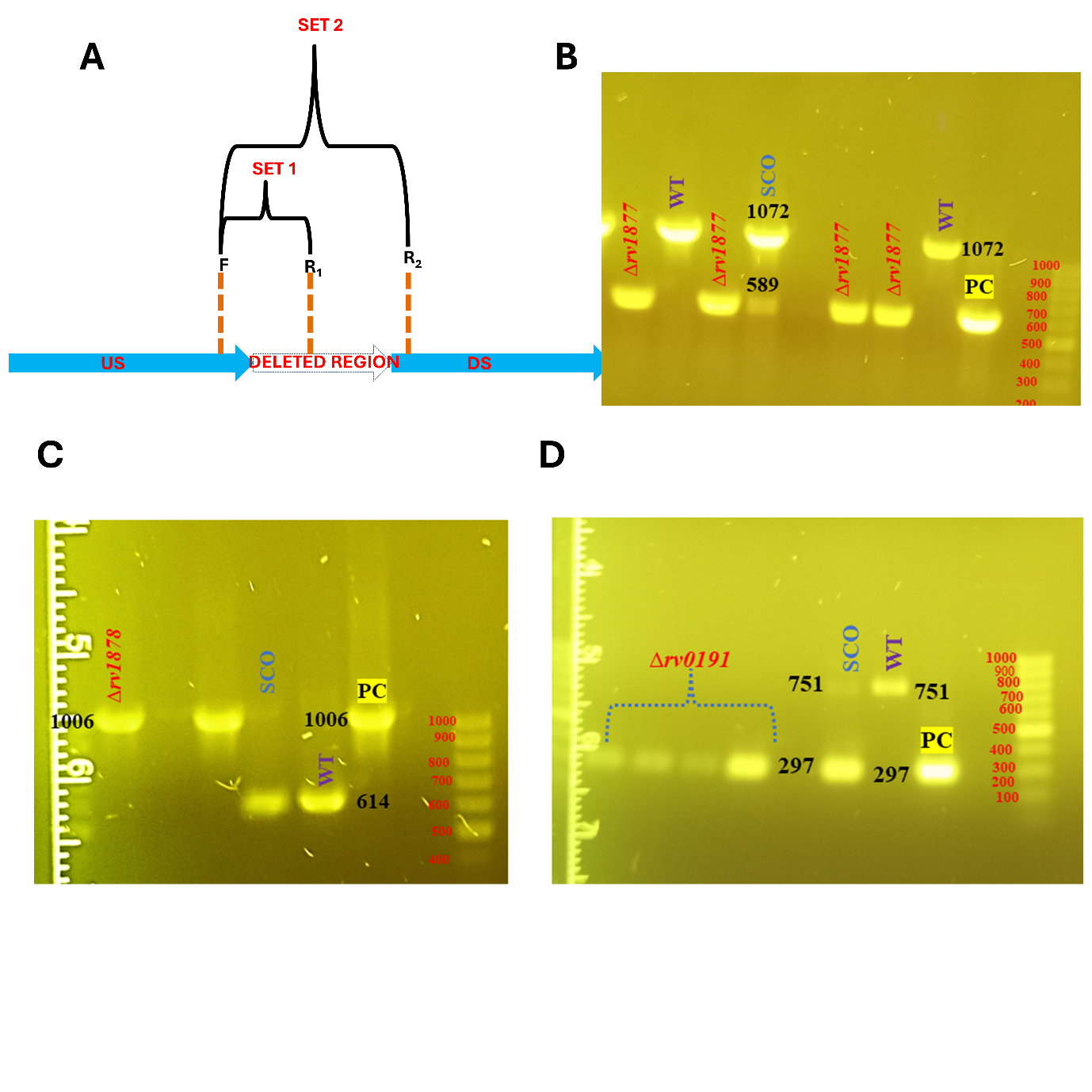


Supplementary Figure.1 **Screening of M.tb mutants by PCR. 1a** Design of screening PCR primers**.** Two reverse complement primers were designed, one binding the deletion region R1 and the second binding downstream the deleted region R2. The mutant should not have the fragment generated by primers F+R1, but the wild-type and the single cross over (SCO) colonies should.**1b Screening of the ∆*rv1877* mutant**, Wild-type (WT): F+R1 = 1072bp, F+R2 = 2593bp, but when both sets are used, only the smaller band is amplified (1072bp). Mutant: F+R1=0 bp, F+R2 =2593-2004= 589bp. Single cross over (SCO): WT + Mutant bands =1072bp + 589bp. PC: positive control, is the deletion construct that was used to generate the mutant, and therefore should display the mutant band (589bp). **1c Screening of the ∆*rv1878* mutant,** WT: F+R1 = 614bp, F+R2 = 2251bp, But when both sets are used, only the smaller band is amplified (614bp), Mutant: F+R1=0 bp, F+R2 =2593-2004= 1006bp, SCO: WT+ Mutant bands= 614bp + 1006bp. **1d Screening of the ∆*rv0191* mutant,** WT: F+R1 = 751bp, F+R2 = 1395bp, But when both sets are used, only the smaller band is amplified (751bp), Mutant: F+R1=0 bp, F+R2 =2593-2004= 297bp, Single cross over (SCO): WT + Mutant= 751bp + 297bp.

## **Supplementary Figure.2**


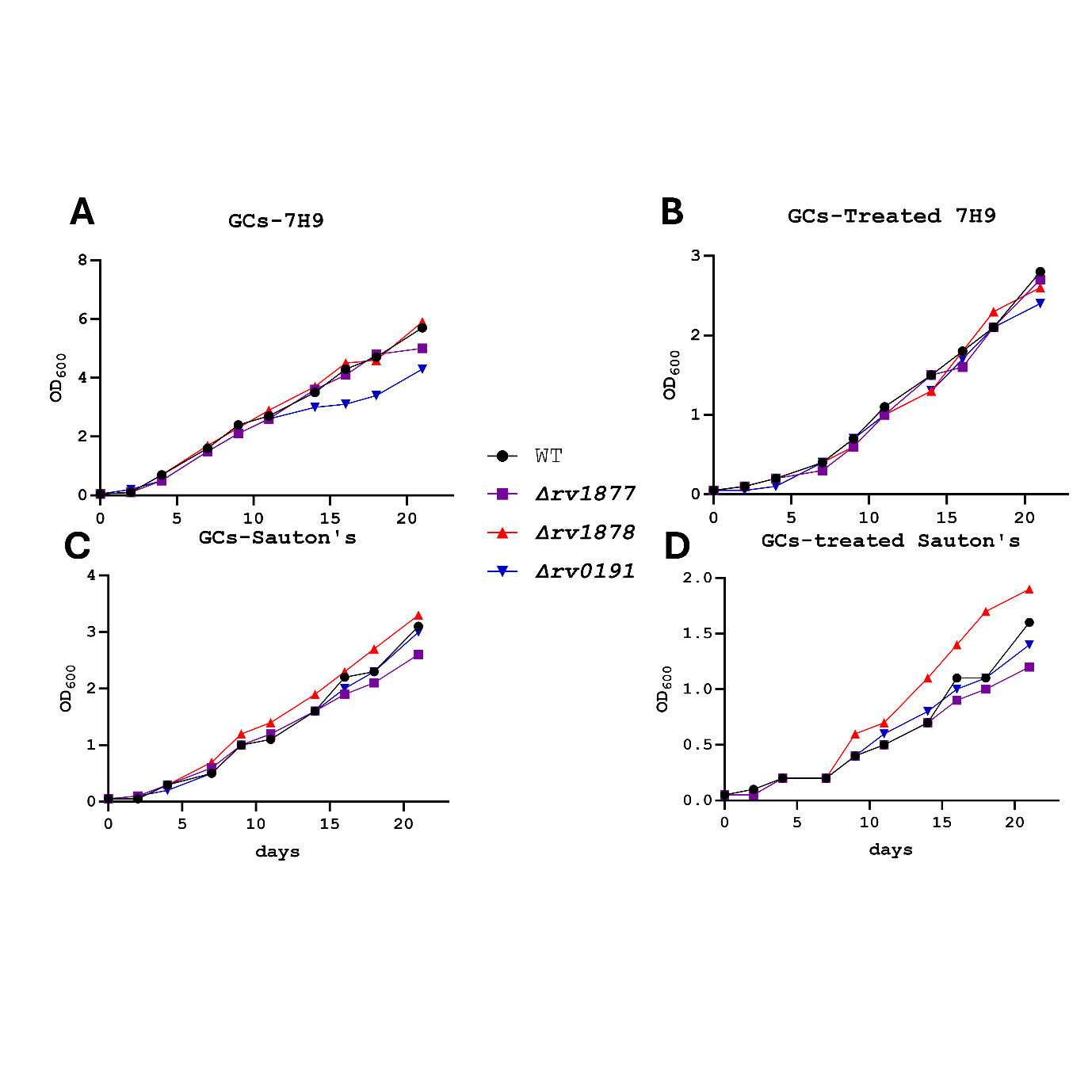


Supplementary Figure.2 **Growth curves (GCs) of the mutants. 2a.** The growth curve of the mutants was compared to the wild-type’s in 7H9-ADS, and there was no difference. **2b.** The growth curve of the same mutants was compared to that of the wild-type in the same media containing 3mM spermine, yet there was no difference. **2c.** The growth curve of the mutants was compared to that of the wild-type in Sauton’s media, and there was no difference. **2d.** The growth of the mutants was finally compared to that of the wild-type in Sauton’s media containing a final concentration of 80µM spermine. And the ∆*rv1877* mutant appeared to have a slight growth defect while the ∆*rv187*8 mutant appeared to have an enhanced growth in this case.

## **Supplementary Figure.3**


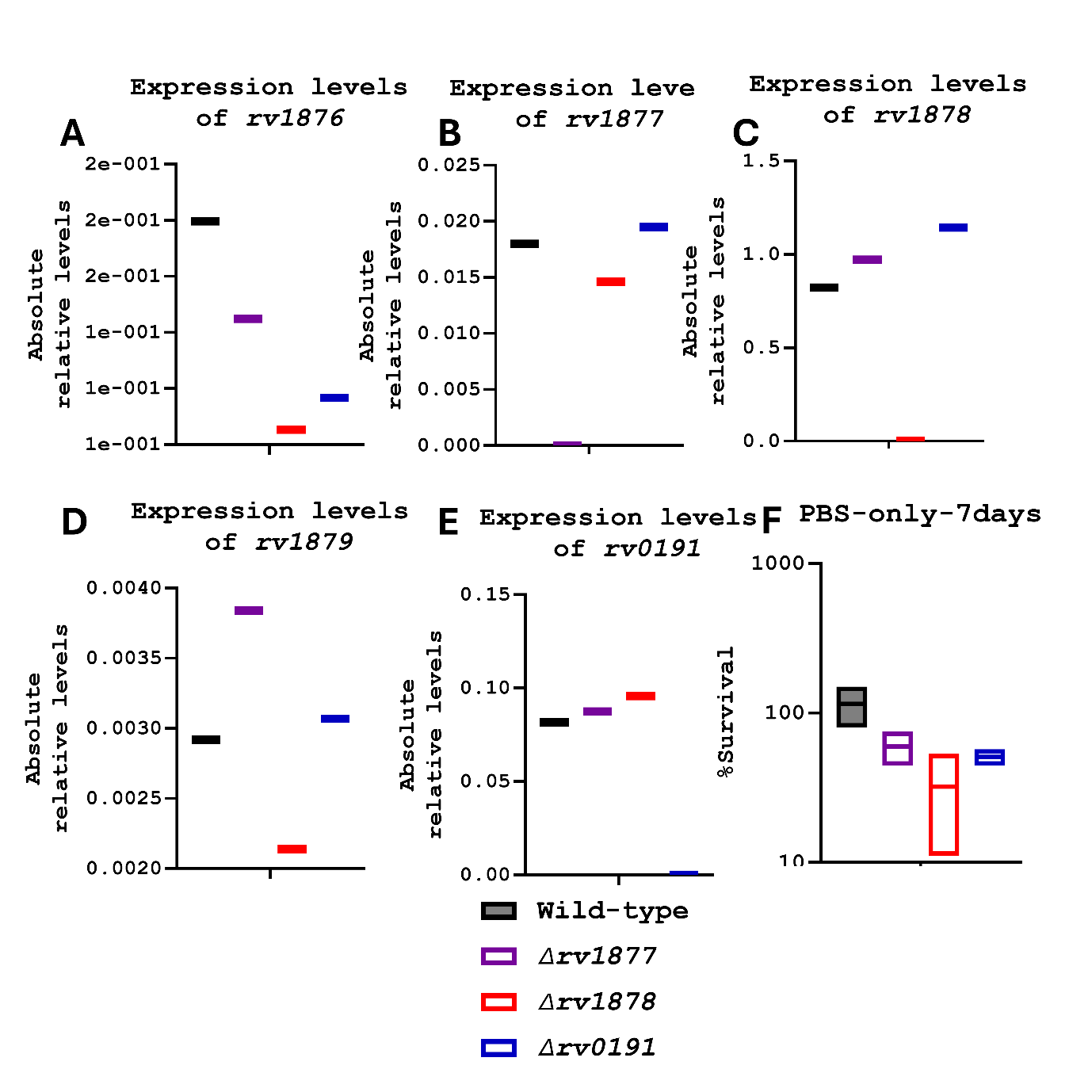


Supplementary Figure.3 **Expression levels of specific genes in the wild-type and mutants generated in this study and evaluation of their survival during nutrient starvation (PBS).** **3a.** the gene *rv1876* was expressed in every strain, but appeared downregulated in all mutants. **3b.** the gene *rv1877* was expressed in every strain, except in the ∆*rv1877* mutant. **3c.** the gene *rv1878* was expressed in every strain, except in the ∆*rv1878* mutant. **3d.** the gene *rv1879* was expressed in every strain. **3e.** the gene *rv0191* was expressed in every strain except in the ∆*rv0191* mutant. **3g** All strains were exposed to nutrient starvation for 7 days. All tested mutants displayed a marginal (P˃0.05) growth defect relative to the wild-type.

## **Supplementary Figure.4**


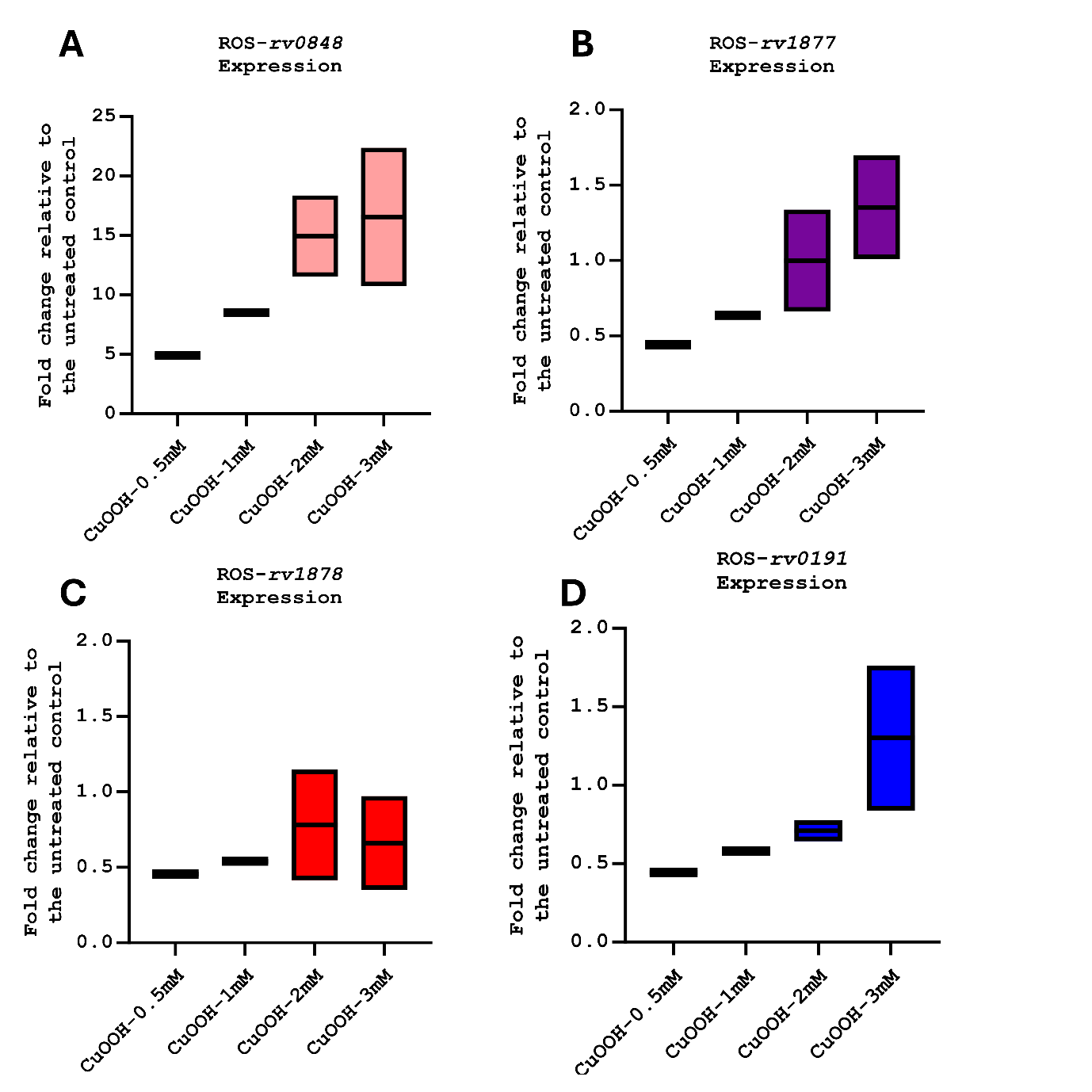


Supplementary Figure.4. **Expression level of specific genes under oxidative stress.** Mycobacteria cultured in 7H9-ADS-Tyl were exposed to a range of concentration of the ROS-donor cumene hydroperoxide (CuOOH) for 3 hours. Then RNA was extracted, and the expression levels were determined and normalized to the untreated controls to derive the fold change. **4a.** The expression of *cysK_2_* (*rv0848*) increased as the concentration of the level of ROS increased, but seemed to be saturated at higher concentrations (around 20-fold change). **4b.** Though they seem a gradual increase in the expression of *rv1877* as the concentration of CuOOH increased, the expression level of *rv1877* was not higher than the untreated control (fold-change ≤1)**. 4c.** There was no change at all in the expression level of *rv1878*. **4d.** The expression level of *rv0191* increased as the concentration of CuOOH increased, but it was not higher than the untreated control (fold-change ≤1).

## **Supplementary Figure.5**


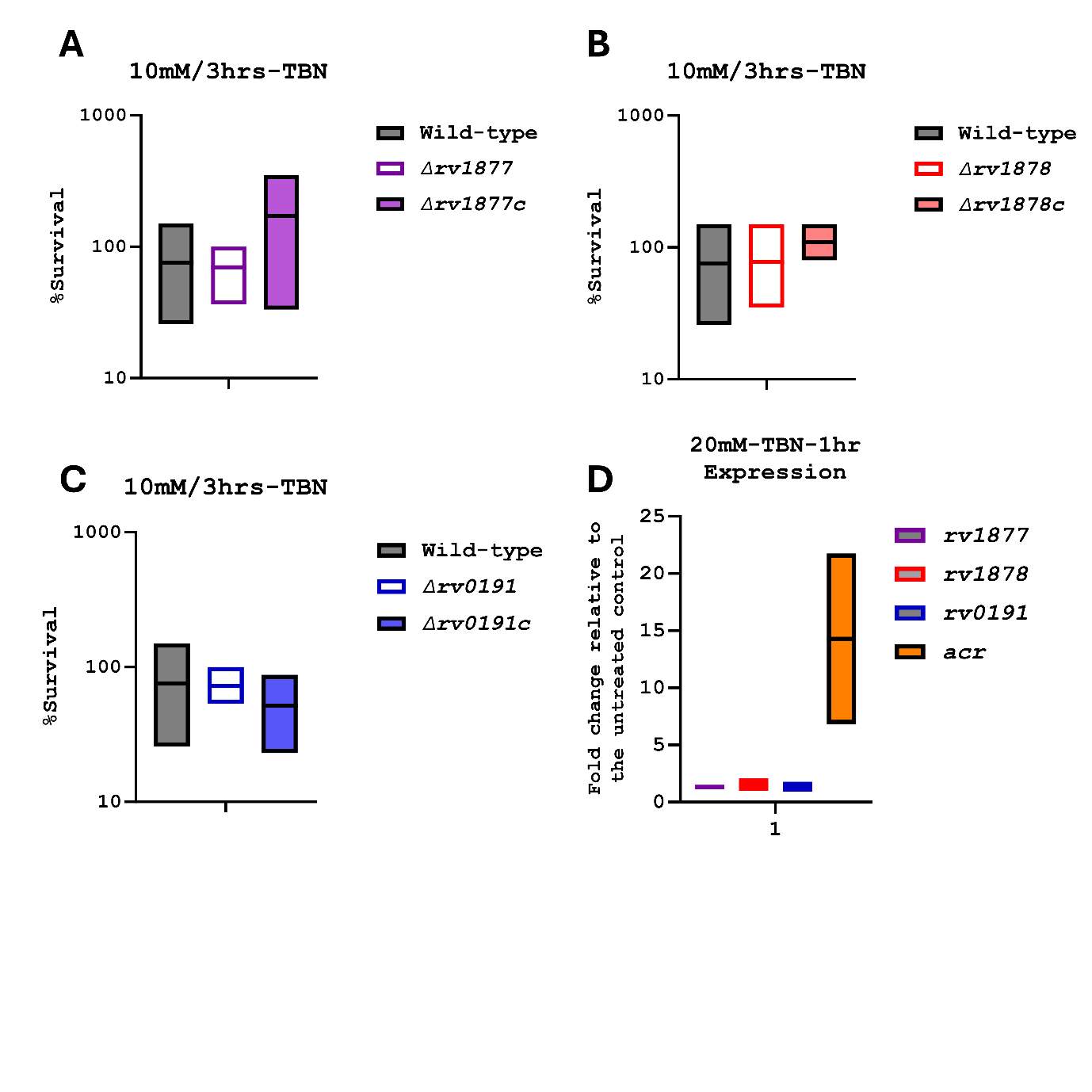


Supplementary Figure 5. **Survival of the strains and the expression level of related genes under nitrosative stress generated by tert-butyl nitrite (TBN). 5a.** Survival of the ∆*rv1877* mutant when exposed to 10mM TBN for 3 hours. It was not different to the wild-type **5b.** The ∆*rv1878* mutant was also not different to the wild-type. **5c.** The ∆*rv0191* mutant was not either. **5d.** Their expression levels after exposure to 20mM TBN for 1 hour, was unchanged, the *acr* gene however, was up-regulated

## **Supplementary Figure.6**


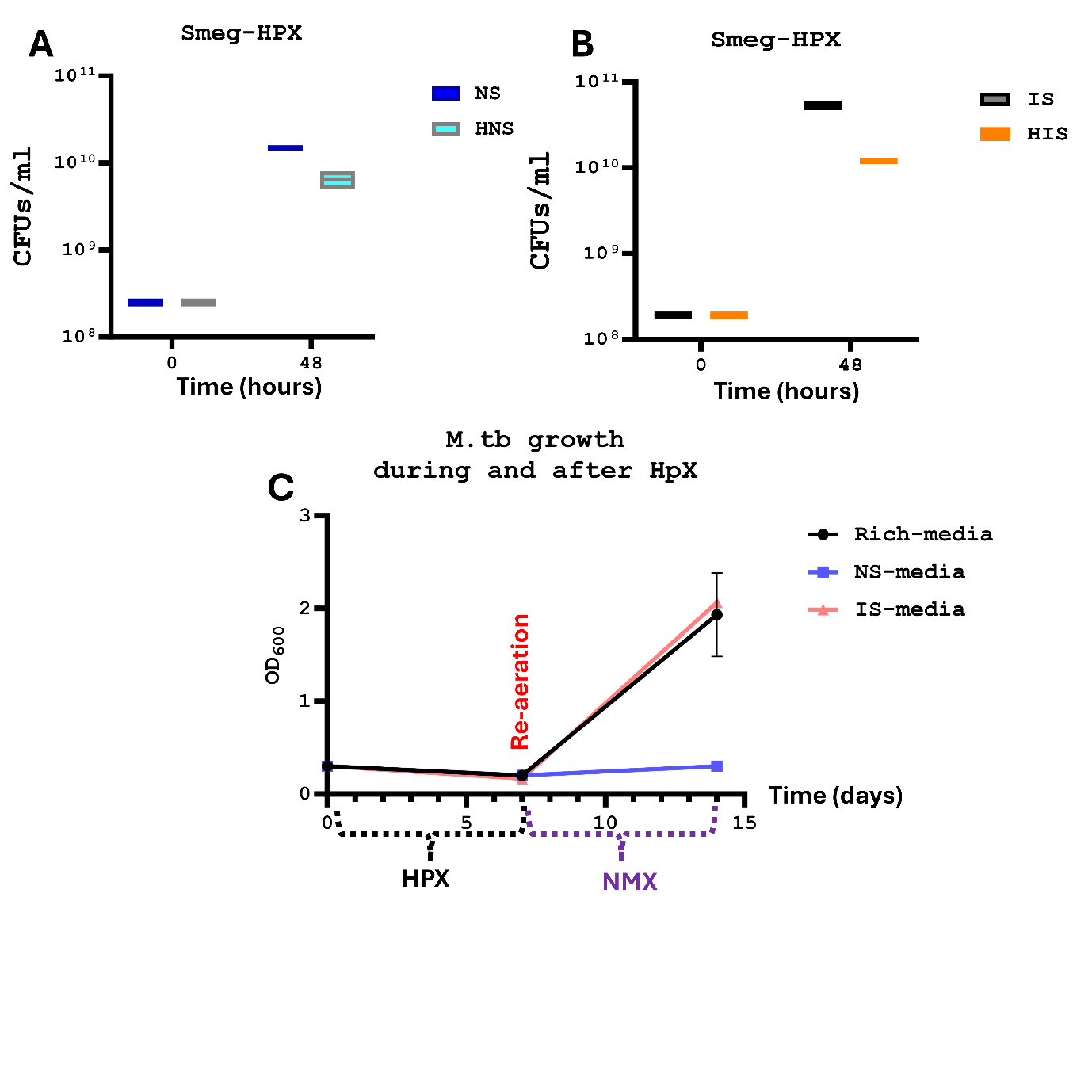


Supplementary Figure 6. **Evaluation of the effect the hypoxia system on the viability of the fast-growing mycobacteria *M. smegmatis* and the slow-growing mycobacteria M.tb. 6a** *M. smegmatis* cultures were washed several times and resuspended (OD_600_~ 0.6), in the nutrient starvation media (NS, PBS) and incubated in aerobic/normoxic (NS) and hypoxic conditions (HNS). CFUs were determined before incubation and 48 hours later in both the hypoxic (HNS) and normoxic (NS) conditions. The bacteria could still multiply but to a lesser extend in the hypoxic condition (HNS). **6b** *M. smegmatis* cultures were treated similarly, but this time in the iron starvation media (IS). The bacteria seemed to grow better (compared to the NS condition), but to a lesser extend in hypoxia (HIS) compared to the normoxic condition (IS). **6c** A logarithmic M.tb culture, washed in PBS was resuspended in triplicate to an OD_600_~ 0.2, in either the IS media, or the NS media, or the 7H9-ADS-Tyl (rich media). They were incubated for 7 days in the hypoxia system (HPX). The OD_600_ measured 7 days later did not change. However, when it was incubated for another 7 days in an aerated (normoxia, NMX) environment, the OD_600_ increased in the IS and rich media, but not in the NS media.

## **Supplementary Figure.7**


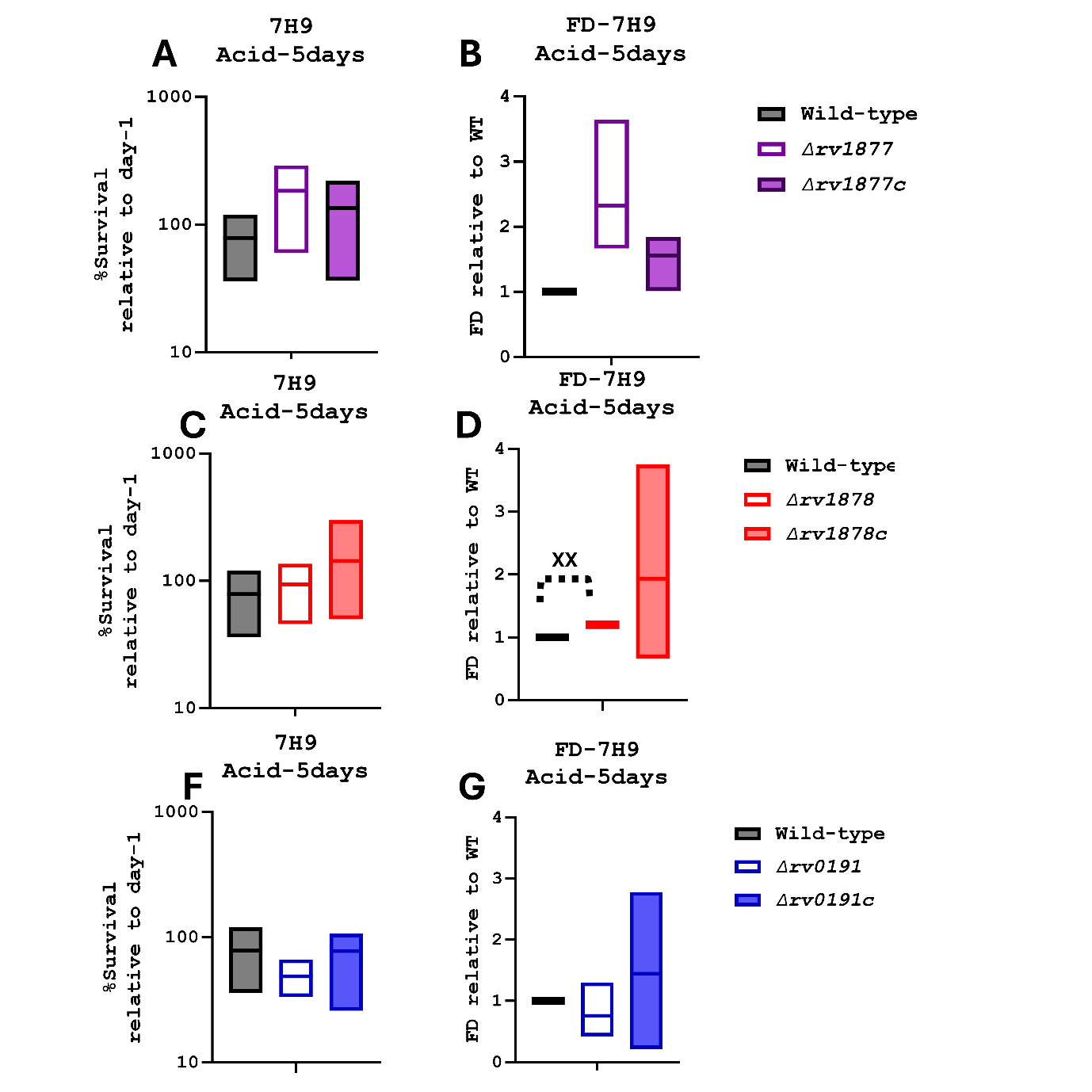


Supplementary Figure 7**. Evaluation of the survival of the mutants to an extended (5 days) exposure to acidified 7H9-ADS-Tyl.** The survival percentage of each mutant was compared to the wild-type’s (7a, c and f) and there was not a difference. But when the percentage of each mutant was normalized to the wild-type’s (7b, d, g), the ∆*rv1878* mutant appeared to be significantly (P=0.006) resistant relative to the wild-type. Alpha was set to 0.05, *P<0.05, **P<0.01, ***P< 0.001, ****P<0.0001

## **Supplementary Figure.8**


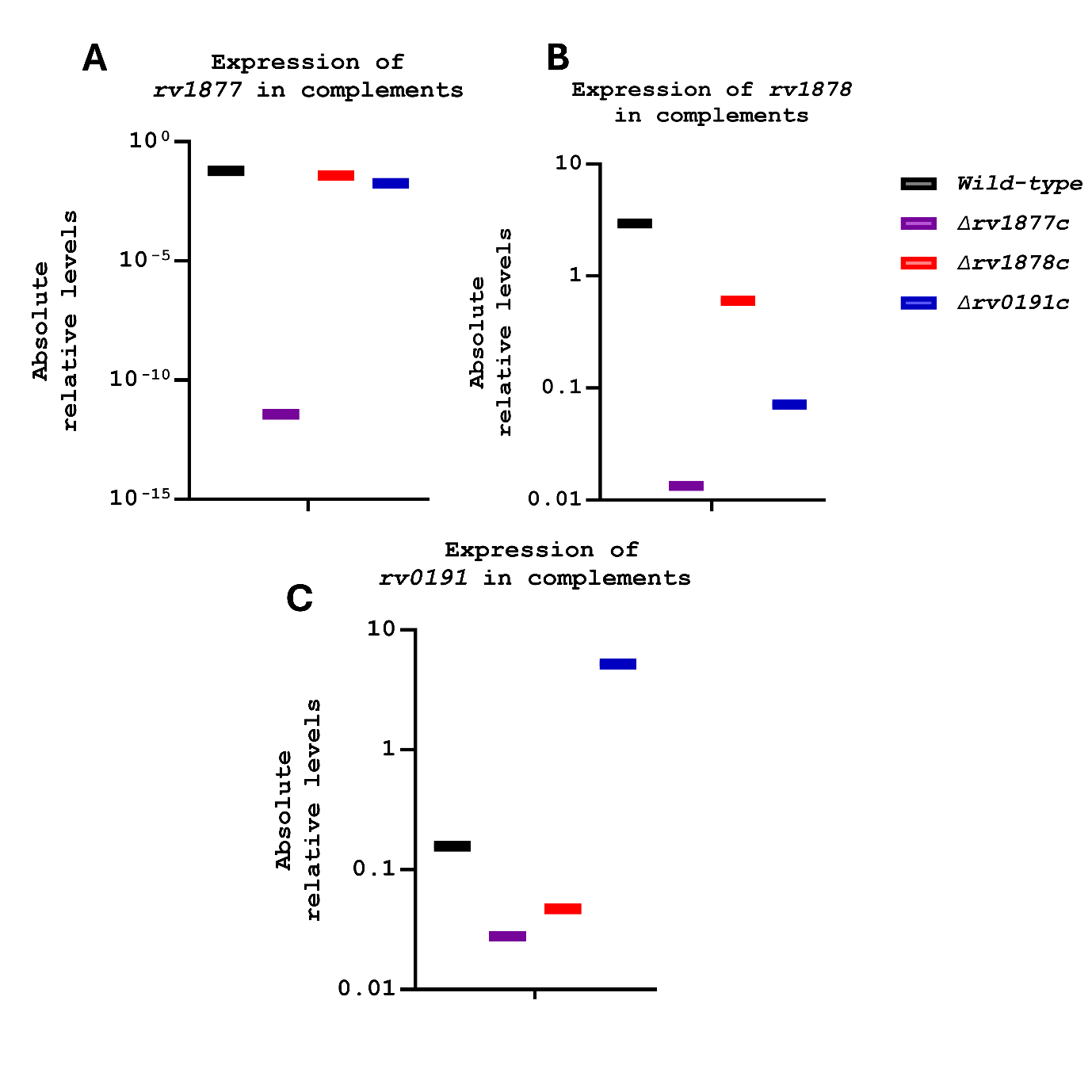


Supplementary Figure.8 **Expression level of specific genes in the wild-type and the complemented strains of the mutant in Sauton’s media. 8a.** the expression of *rv1877* was only marginally restored in the complement of the ∆*rv1877* mutant. **8b.** the expression of *rv1878* was only marginally restored in the complement of the ∆*rv1878* mutant. **8c**. the expression of *rv0191* was over-expressed (~50-fold) in the complement of the ∆*rv0191* mutant.

# **References**

1. Krysenko, S. *et al.* GlnA3Mt is able to glutamylate spermine but it is not essential for the detoxification of spermine in Mycobacterium tuberculosis. *bioRxiv* 2023.12.14.571729 (2023) doi:10.1101/2023.12.14.571729.

2. Parish, T. & Stoker, N. G. Use of a flexible cassette method to generate a double unmarked Mycobacterium tuberculosis tlyA plcABC mutant by gene replacement. *Microbiology* **146 ( Pt 8**, 1969–1975 (2000).

3. Andreu, N. *et al.* Optimisation of bioluminescent reporters for use with mycobacteria. *PLoS One* **5**, e10777 (2010).

4. Carroll, P., Muwanguzi-Karugaba, J. & Parish, T. Codon-optimized DsRed fluorescent protein for use in Mycobacterium tuberculosis. *BMC Res. Notes* **11**, 1–6 (2018).

5. Sao Emani, Carine; Reiling, Norbert (2023). The Mycobacterium tuberculosis efflux pumps Rv1877 and Rv0191 play differential roles in the protection of the pathogen against chemical stress. figshare. Collection. https://figshare.com/collections/_/7001352
